# Supplementary material for: Juxtaposition of heterozygous and homozygous regions causes reciprocal crossover remodelling via interference during Arabidopsis meiosis
Source: eLife. 2015 Mar 27;4:e03708. doi: 10.7554/eLife.03708 (PMC4407271; doi:10.7554/eLife.03708)
Supplement: Figure 2—source data 1. — DOI: http://dx.doi.org/10.7554/eLife.03708.008 [file elife03708s001.docx]

**Figure 2 – Source Data 1. *420* crossover frequency measured via manual or automated scoring of seed fluorescence.** For the formula used for cM calculation please see Materials and Methods.

| Method | Genotype | Green alone | Red alone | Both | None | Total | cM |
| --- | --- | --- | --- | --- | --- | --- | --- |
| Manual | Col/Col | 72 | 86 | 680 | 163 | 1001 | 17.28 |
| Manual | Col/Col | 96 | 107 | 734 | 178 | 1115 | 20.26 |
| Manual | Col/Col | 89 | 100 | 727 | 182 | 1098 | 19.02 |
| Manual | Col/Ler | 74 | 79 | 799 | 204 | 1156 | 14.25 |
| Manual | Col/Ler | 69 | 64 | 705 | 224 | 1062 | 13.42 |
| Manual | Col/Ler | 60 | 68 | 717 | 164 | 1009 | 13.61 |
| Manual | Col/Sha | 76 | 61 | 1540 | 475 | 2152 | 6.58 |
| Manual | Col/Sha | 65 | 80 | 1663 | 466 | 2274 | 6.59 |
| Manual | Col/Sha | 83 | 89 | 1655 | 527 | 2354 | 7.6 |
| Auto | Col/Col | 190 | 242 | 1658 | 365 | 2455 | 19.5 |
| Auto | Col/Col | 234 | 253 | 1984 | 512 | 2983 | 17.93 |
| Auto | Col/Col | 217 | 240 | 1904 | 490 | 2851 | 17.57 |
| Auto | Col/Ler | 200 | 226 | 2154 | 603 | 3183 | 14.42 |
| Auto | Col/Ler | 196 | 197 | 2084 | 547 | 3024 | 13.97 |
| Auto | Col/Ler | 181 | 206 | 2066 | 599 | 3052 | 13.61 |
| Auto | Col/Sha | 82 | 72 | 1691 | 525 | 2370 | 6.72 |
| Auto | Col/Sha | 89 | 87 | 1791 | 512 | 2479 | 7.37 |
| Auto | Col/Sha | 79 | 83 | 1737 | 450 | 2349 | 7.15 |
