# Supplementary material for: Juxtaposition of heterozygous and homozygous regions causes reciprocal crossover remodelling via interference during Arabidopsis meiosis
Source: eLife. 2015 Mar 27;4:e03708. doi: 10.7554/eLife.03708 (PMC4407271; doi:10.7554/eLife.03708)
Supplement: Figure 3—source data 4. — DOI: http://dx.doi.org/10.7554/eLife.03708.014 [file elife03708s005.docx]

**Figure 3 – Source Data 4. *I1b* F_1_ flow cytometry count data.** cM were calculated as 100 x R5/(R3+R5).

| Cross | Replicate | Total pollen | Red alone (Gate R2) | Red and green (Gate R3) | Neither  (Gate R4) | Green alone (Gate R5) | cM |
| --- | --- | --- | --- | --- | --- | --- | --- |
| Bay-0 | 1 | 27787 | 1535 | 11663 | 13522 | 1067 | 8.38 |
| Bay-0 | 2 | 27704 | 1406 | 11684 | 13510 | 1104 | 8.63 |
| Bay-0 | 3 | 27570 | 1417 | 11550 | 13488 | 1115 | 8.8 |
| Bay-0 | 4 | 30544 | 2153 | 9178 | 18331 | 882 | 8.77 |
| Bur-0 | 1 | 27563 | 1382 | 11333 | 14011 | 837 | 6.88 |
| Bur-0 | 2 | 28010 | 1351 | 12041 | 13778 | 840 | 6.52 |
| Bur-0 | 3 | 27591 | 1249 | 12141 | 13313 | 888 | 6.82 |
| Bur-0 | 4 | 29853 | 1398 | 12136 | 15487 | 832 | 6.42 |
| Bur-0 | 5 | 25688 | 1261 | 10544 | 13085 | 798 | 7.04 |
| C24 | 1 | 26781 | 1386 | 11157 | 13180 | 1058 | 8.66 |
| C24 | 2 | 26916 | 1355 | 11004 | 13484 | 1073 | 8.88 |
| C24 | 3 | 25459 | 1332 | 10300 | 12783 | 1044 | 9.2 |
| C24 | 4 | 27046 | 1290 | 11590 | 13117 | 1049 | 8.3 |
| Can-0 | 1 | 5186 | 350 | 2309 | 2326 | 201 | 8.01 |
| Can-0 | 2 | 30568 | 3272 | 10498 | 15944 | 854 | 7.52 |
| Can-0 | 3 | 13856 | 1172 | 6091 | 6094 | 499 | 7.57 |
| Can-0 | 4 | 12186 | 1117 | 5338 | 5296 | 435 | 7.54 |
| Can-0 | 5 | 28869 | 1998 | 10003 | 15950 | 918 | 8.41 |
| Can-0 | 6 | 28189 | 2342 | 10457 | 14511 | 879 | 7.75 |
| Can-0 | 7 | 29655 | 2445 | 10618 | 15671 | 921 | 7.98 |
| Col-0 | 1 | 26848 | 1418 | 11493 | 12984 | 953 | 7.66 |
| Col-0 | 2 | 27537 | 1376 | 11935 | 13217 | 1009 | 7.8 |
| Col-0 | 3 | 27809 | 1478 | 11713 | 13528 | 1090 | 8.51 |
| Col-0 | 4 | 26081 | 1242 | 11664 | 12148 | 1027 | 8.09 |
| Col-0 | 5 | 25191 | 1470 | 10145 | 12690 | 886 | 8.03 |
| Col-0 | 6 | 26417 | 1280 | 11492 | 12621 | 1024 | 8.18 |
| Col-0 | 7 | 26806 | 1260 | 11571 | 13050 | 925 | 7.4 |
| Col-0 | 8 | 29753 | 1465 | 12622 | 14641 | 1025 | 7.51 |
| Col-0 | 9 | 29722 | 1600 | 12330 | 14690 | 1102 | 8.2 |
| Col-0 | 10 | 29807 | 1536 | 12918 | 14269 | 1084 | 7.74 |
| Col-0 | 11 | 30732 | 2935 | 10183 | 16752 | 862 | 7.8 |
| Col-0 | 12 | 31074 | 3405 | 9306 | 17509 | 854 | 8.41 |
| Col-0 | 13 | 29713 | 2760 | 9806 | 16253 | 894 | 8.36 |
| Ct-1 | 1 | 26912 | 1719 | 9973 | 14330 | 890 | 8.19 |
| Ct-1 | 2 | 26910 | 1846 | 9762 | 14460 | 842 | 7.94 |
| Ct-1 | 3 | 27992 | 1782 | 10585 | 14754 | 871 | 7.6 |
| Ct-1 | 4 | 26051 | 1845 | 9938 | 13462 | 806 | 7.5 |
| Cvi-0 | 1 | 27802 | 1697 | 11313 | 13694 | 1098 | 8.85 |
| Cvi-0 | 2 | 27266 | 1915 | 10379 | 13821 | 1151 | 9.98 |
| Cvi-0 | 3 | 28034 | 1732 | 11510 | 13643 | 1149 | 9.08 |
| Cvi-0 | 4 | 27529 | 1692 | 11099 | 13693 | 1045 | 8.61 |
| Edi-0 | 1 | 32614 | 2160 | 11740 | 17706 | 1008 | 7.91 |
| Edi-0 | 2 | 31219 | 2101 | 11836 | 16227 | 1055 | 8.18 |
| Edi-0 | 3 | 31497 | 2060 | 11690 | 16722 | 1025 | 8.06 |
| Edi-0 | 4 | 31196 | 2351 | 10096 | 17909 | 840 | 7.68 |
| Hi-0 | 1 | 26724 | 1289 | 11055 | 13580 | 800 | 6.75 |
| Hi-0 | 2 | 25835 | 1334 | 10509 | 13253 | 739 | 6.57 |
| Hi-0 | 3 | 25384 | 1247 | 10343 | 13024 | 770 | 6.93 |
| Ws-0 | 1 | 16174 | 1356 | 5628 | 8789 | 401 | 6.65 |
| Ws-0 | 2 | 17263 | 1270 | 6261 | 9272 | 460 | 6.84 |
| Ws-0 | 3 | 23025 | 1779 | 8852 | 11774 | 620 | 6.55 |
| Ws-0 | 4 | 16632 | 1380 | 5856 | 8977 | 419 | 6.68 |
| Zu-0 | 1 | 29395 | 1268 | 10578 | 16710 | 839 | 7.35 |
| Zu-0 | 2 | 22171 | 799 | 7566 | 13129 | 677 | 8.21 |
| Zu-0 | 3 | 29165 | 1283 | 9973 | 17152 | 757 | 7.05 |
| Zu-0 | 4 | 29342 | 1212 | 10231 | 17097 | 802 | 7.27 |
| Kas | 1 | 28007 | 1766 | 11110 | 14328 | 803 | 6.74 |
| Kas | 2 | 27317 | 1525 | 10647 | 14368 | 777 | 6.8 |
| Kas | 3 | 27665 | 1471 | 10865 | 14474 | 855 | 7.3 |
| Kn-0 | 1 | 30159 | 1887 | 12678 | 14563 | 1031 | 7.52 |
| Kn-0 | 2 | 30197 | 1867 | 12311 | 15039 | 980 | 7.37 |
| Kn-0 | 3 | 30150 | 1819 | 12400 | 14936 | 995 | 7.43 |
| Kond | 1 | 26732 | 1184 | 11074 | 13616 | 858 | 7.19 |
| Kond | 2 | 26201 | 1282 | 11074 | 13001 | 844 | 7.08 |
| Kond | 3 | 27620 | 1333 | 11557 | 13855 | 875 | 7.04 |
| Ler-0 | 1 | 27902 | 1262 | 12773 | 13006 | 861 | 6.32 |
| Ler-0 | 2 | 28098 | 1322 | 12605 | 13251 | 920 | 6.8 |
| Ler-0 | 3 | 32256 | 1877 | 14664 | 14689 | 1026 | 6.54 |
| Mt-0 | 1 | 30516 | 1832 | 12594 | 14804 | 1286 | 9.27 |
| Mt-0 | 2 | 49858 | 3042 | 19480 | 25224 | 2112 | 9.78 |
| Mt-0 | 3 | 49876 | 2642 | 17768 | 27562 | 1904 | 9.68 |
| No-0 | 1 | 30256 | 4433 | 2662 | 22927 | 234 | 8.08 |
| No-0 | 2 | 30069 | 4633 | 3065 | 22122 | 249 | 7.51 |
| No-0 | 3 | 30107 | 4935 | 2814 | 22159 | 199 | 6.6 |
| Oy-0 | 1 | 29791 | 2239 | 11896 | 14664 | 992 | 7.7 |
| Oy-0 | 2 | 29675 | 2385 | 11543 | 14849 | 898 | 7.22 |
| Oy-0 | 3 | 29598 | 2424 | 10474 | 15755 | 945 | 8.28 |
| Oy-0 | 4 | 29844 | 3105 | 9593 | 16348 | 798 | 7.68 |
| Po-0 | 1 | 29833 | 3267 | 5459 | 20643 | 464 | 7.83 |
| Po-0 | 2 | 29837 | 3743 | 5022 | 20680 | 392 | 7.24 |
| Po-0 | 3 | 29875 | 4034 | 3722 | 21846 | 273 | 6.83 |
| Po-0 | 4 | 29606 | 3833 | 4300 | 21146 | 327 | 7.07 |
| Rsch-4 | 1 | 28830 | 3245 | 10074 | 14657 | 854 | 7.81 |
| Rsch-4 | 2 | 29178 | 2780 | 9969 | 15517 | 912 | 8.38 |
| Rsch-4 | 3 | 28984 | 2656 | 10605 | 14845 | 878 | 7.65 |
| Sf-2 | 1 | 30036 | 2260 | 11284 | 15528 | 964 | 7.87 |
| Sf-2 | 2 | 31235 | 2186 | 11939 | 15990 | 1120 | 8.58 |
| Sf-2 | 3 | 30253 | 2216 | 10549 | 16568 | 920 | 8.02 |
| Sha | 1 | 27066 | 1551 | 10554 | 14068 | 893 | 7.8 |
| Sha | 2 | 27222 | 1664 | 10858 | 13812 | 888 | 7.56 |
| Sha | 3 | 27903 | 1588 | 10875 | 14574 | 866 | 7.38 |
| Sha | 4 | 25258 | 1505 | 10311 | 12541 | 901 | 8.04 |
| Sha | 5 | 24634 | 1429 | 10113 | 12206 | 886 | 8.06 |
| Sha | 6 | 24239 | 1662 | 9385 | 12399 | 793 | 7.79 |
| Tsu-0 | 1 | 26376 | 1050 | 11209 | 13324 | 793 | 6.61 |
| Tsu-0 | 2 | 26560 | 1088 | 11028 | 13658 | 786 | 6.65 |
| Tsu-0 | 3 | 26797 | 1111 | 11672 | 13194 | 820 | 6.56 |
| Wil-2 | 1 | 40253 | 4745 | 12313 | 22393 | 802 | 6.12 |
| Wil-2 | 2 | 40302 | 4966 | 12567 | 21949 | 820 | 6.13 |
| Wil-2 | 3 | 40291 | 4589 | 13694 | 21150 | 858 | 5.9 |
| Wil-2 | 4 | 40278 | 5065 | 12241 | 22145 | 827 | 6.33 |
| Wu-0 | 1 | 26999 | 1643 | 11032 | 13489 | 835 | 7.04 |
| Wu-0 | 2 | 27616 | 1517 | 11196 | 13984 | 919 | 7.59 |
| Wu-0 | 3 | 27176 | 2650 | 8239 | 15606 | 681 | 7.63 |
| Wu-0 | 4 | 27150 | 2727 | 7667 | 16099 | 657 | 7.89 |
| Wu-0 | 5 | 27534 | 3330 | 7324 | 16247 | 633 | 7.96 |
| Wu-0 | 6 | 28161 | 3355 | 6225 | 18098 | 483 | 7.2 |

**Figure 3 – Source Data 2. *I1fg* F_1_ flow cytometry count data.** cM were calculated as 100 x R5/(R3+R5).

| Cross | Replicate | Total pollen | Red alone (Gate R2) | Red and Green (Gate R3) | Neither (Gate R4) | Green alone (Gate R5) | cM |
| --- | --- | --- | --- | --- | --- | --- | --- |
| Bay-0 | 1 | 41373 | 3364 | 14996 | 21672 | 1341 | 8.21 |
| Bay-0 | 2 | 41360 | 2866 | 16218 | 20793 | 1483 | 8.38 |
| Bay-0 | 3 | 40912 | 2894 | 16307 | 20238 | 1473 | 8.28 |
| Bur-0 | 1 | 52132 | 3228 | 15782 | 31486 | 1636 | 9.39 |
| Bur-0 | 2 | 51777 | 3164 | 17349 | 29517 | 1747 | 9.15 |
| Bur-0 | 3 | 52197 | 3461 | 15648 | 31578 | 1510 | 8.8 |
| Bur-0 | 4 | 52870 | 4221 | 14324 | 32925 | 1400 | 8.9 |
| C24 | 1 | 30376 | 1918 | 11262 | 16068 | 1128 | 9.1 |
| C24 | 2 | 15208 | 1150 | 5306 | 8273 | 479 | 8.28 |
| C24 | 3 | 30438 | 2250 | 10904 | 16302 | 982 | 8.26 |
| C24 | 4 | 30349 | 2172 | 11274 | 15898 | 1005 | 8.18 |
| Can-0 | 1 | 30380 | 1908 | 12265 | 15055 | 1152 | 8.59 |
| Can-0 | 2 | 30192 | 1585 | 13040 | 14346 | 1221 | 8.56 |
| Can-0 | 3 | 30238 | 1812 | 12684 | 14627 | 1115 | 8.08 |
| Can-0 | 4 | 30256 | 1743 | 12322 | 15036 | 1155 | 8.57 |
| Col-0 | 1 | 31886 | 1848 | 12052 | 16928 | 1058 | 8.07 |
| Col-0 | 2 | 39246 | 2194 | 15293 | 20410 | 1349 | 8.11 |
| Col-0 | 3 | 40488 | 2388 | 16145 | 20518 | 1437 | 8.17 |
| Col-0 | 4 | 30170 | 1550 | 12571 | 14919 | 1130 | 8.25 |
| Col-0 | 5 | 30177 | 1601 | 13343 | 14041 | 1192 | 8.2 |
| Col-0 | 6 | 30231 | 1605 | 13158 | 14296 | 1172 | 8.18 |
| Ct-1 | 1 | 40484 | 4011 | 12927 | 22282 | 1264 | 8.91 |
| Ct-1 | 2 | 40534 | 3788 | 13007 | 22513 | 1226 | 8.61 |
| Ct-1 | 3 | 40374 | 3945 | 12686 | 22536 | 1207 | 8.69 |
| Ct-1 | 4 | 40569 | 3867 | 12879 | 22616 | 1207 | 8.57 |
| Cvi-0 | 1 | 42948 | 3334 | 17495 | 20194 | 1925 | 9.91 |
| Cvi-0 | 2 | 42179 | 2996 | 17308 | 19922 | 1953 | 10.14 |
| Cvi-0 | 3 | 42383 | 2983 | 17416 | 20060 | 1924 | 9.95 |
| Cvi-0 | 4 | 41199 | 2899 | 17088 | 19313 | 1899 | 10 |
| Edi-0 | 1 | 21123 | 1406 | 6872 | 12279 | 566 | 7.61 |
| Edi-0 | 2 | 20989 | 1244 | 7048 | 12099 | 598 | 7.82 |
| Edi-0 | 3 | 29371 | 2235 | 7648 | 18785 | 703 | 8.42 |
| Edi-0 | 4 | 29469 | 2085 | 8374 | 18271 | 739 | 8.11 |
| Edi-0 | 5 | 29510 | 2018 | 8511 | 18254 | 727 | 7.87 |
| Hi-0 | 1 | 40409 | 3484 | 14092 | 21743 | 1090 | 7.18 |
| Hi-0 | 2 | 39526 | 3092 | 14279 | 21097 | 1058 | 6.9 |
| Hi-0 | 3 | 39567 | 3029 | 14410 | 21083 | 1045 | 6.76 |
| Kas | 1 | 29917 | 607 | 4214 | 24715 | 381 | 8.29 |
| Kas | 2 | 29934 | 1116 | 10279 | 17584 | 955 | 8.5 |
| Kas | 3 | 29926 | 1195 | 11211 | 16432 | 1088 | 8.85 |
| Kas | 4 | 29900 | 1470 | 12104 | 15179 | 1147 | 8.66 |
| Kn-0 | 1 | 19981 | 1434 | 8628 | 9320 | 599 | 6.49 |
| Kn-0 | 2 | 29755 | 2012 | 12921 | 13893 | 929 | 6.71 |
| Kn-0 | 3 | 29845 | 2211 | 12921 | 13807 | 906 | 6.55 |
| Kn-0 | 4 | 29887 | 2137 | 13197 | 13629 | 924 | 6.54 |
| Kond | 1 | 29977 | 1535 | 13352 | 13882 | 1208 | 8.3 |
| Kond | 2 | 29997 | 1462 | 12967 | 14425 | 1143 | 8.1 |
| Kond | 3 | 29978 | 1487 | 12986 | 14407 | 1098 | 7.8 |
| Kond | 4 | 30011 | 1431 | 13423 | 13967 | 1190 | 8.14 |
| Ler-0 | 1 | 42460 | 2365 | 18709 | 19681 | 1705 | 8.35 |
| Ler-0 | 2 | 41191 | 2176 | 18127 | 19312 | 1576 | 8 |
| Ler-0 | 3 | 41599 | 2153 | 17783 | 20042 | 1621 | 8.35 |
| Ler-0 | 4 | 41421 | 2388 | 18052 | 19364 | 1617 | 8.22 |
| Mt-0 | 1 | 31450 | 2143 | 10550 | 17838 | 919 | 8.01 |
| Mt-0 | 2 | 31182 | 2164 | 11541 | 16512 | 965 | 7.72 |
| Mt-0 | 3 | 30978 | 2143 | 10911 | 17003 | 921 | 7.78 |
| No-0 | 1 | 30153 | 2002 | 11848 | 15310 | 993 | 7.73 |
| No-0 | 2 | 30124 | 1886 | 12179 | 15003 | 1056 | 7.98 |
| No-0 | 3 | 30100 | 1931 | 11659 | 15502 | 1008 | 7.96 |
| No-0 | 4 | 30193 | 2093 | 11337 | 15810 | 953 | 7.75 |
| Oy-0 | 1 | 30090 | 2075 | 11577 | 15345 | 1093 | 8.63 |
| Oy-0 | 2 | 30072 | 1824 | 12103 | 15060 | 1085 | 8.23 |
| Oy-0 | 3 | 30127 | 2360 | 11153 | 15576 | 1038 | 8.51 |
| Oy-0 | 4 | 30146 | 2277 | 11103 | 15792 | 974 | 8.06 |
| Po-0 | 1 | 42448 | 3239 | 15364 | 22489 | 1356 | 8.11 |
| Po-0 | 2 | 41685 | 3089 | 17327 | 19735 | 1534 | 8.13 |
| Po-0 | 3 | 41844 | 3021 | 17654 | 19767 | 1402 | 7.36 |
| Po-0 | 4 | 42850 | 2922 | 12332 | 26503 | 1093 | 8.14 |
| Rsch-4 | 1 | 32610 | 1743 | 13485 | 16366 | 1016 | 7.01 |
| Rsch-4 | 2 | 32688 | 1663 | 13785 | 16236 | 1004 | 6.79 |
| Rsch-4 | 3 | 50531 | 2780 | 20757 | 25492 | 1502 | 6.75 |
| Rsch-4 | 4 | 32078 | 1888 | 12414 | 16900 | 876 | 6.59 |
| Sf-2 | 1 | 30025 | 2057 | 10913 | 15997 | 1058 | 8.84 |
| Sf-2 | 2 | 20256 | 1463 | 7312 | 10757 | 724 | 9.01 |
| Sf-2 | 3 | 30091 | 2074 | 11492 | 15421 | 1104 | 8.76 |
| Sf-2 | 4 | 24889 | 1896 | 8941 | 13197 | 855 | 8.73 |
| Sha | 1 | 30384 | 1623 | 11412 | 16485 | 864 | 7.04 |
| Sha | 2 | 30453 | 1684 | 11674 | 16141 | 954 | 7.55 |
| Sha | 3 | 30345 | 1794 | 10942 | 16697 | 912 | 7.69 |
| Sha | 4 | 30335 | 1970 | 10159 | 17340 | 866 | 7.85 |
| Tsu-0 | 1 | 29882 | 1188 | 12807 | 15051 | 836 | 6.13 |
| Tsu-0 | 2 | 29939 | 1112 | 13027 | 14882 | 918 | 6.58 |
| Tsu-0 | 3 | 29911 | 1294 | 12290 | 15488 | 839 | 6.39 |
| Tsu-0 | 4 | 29914 | 1218 | 12381 | 15536 | 779 | 5.92 |
| Wil-2 | 1 | 30211 | 1701 | 11736 | 15905 | 869 | 6.89 |
| Wil-2 | 2 | 30213 | 1571 | 12594 | 15111 | 937 | 6.92 |
| Wil-2 | 3 | 30255 | 1640 | 11275 | 16526 | 814 | 6.73 |
| Wil-2 | 4 | 30156 | 1668 | 12359 | 15215 | 914 | 6.89 |
| Ws-0 | 1 | 16977 | 1100 | 6167 | 9192 | 518 | 7.75 |
| Ws-0 | 2 | 15384 | 1019 | 5864 | 8001 | 500 | 7.86 |
| Ws-0 | 3 | 29567 | 2871 | 7290 | 18788 | 618 | 7.81 |
| Ws-0 | 4 | 29787 | 2926 | 8003 | 18191 | 667 | 7.69 |
| Ws-0 | 5 | 28900 | 2504 | 7814 | 17938 | 644 | 7.61 |
| Wu-0 | 1 | 30157 | 1689 | 11509 | 16175 | 784 | 6.38 |
| Wu-0 | 2 | 30230 | 1713 | 11976 | 15752 | 789 | 6.18 |
| Wu-0 | 3 | 30325 | 1831 | 11509 | 16185 | 800 | 6.5 |
| Wu-0 | 4 | 30335 | 1791 | 10945 | 16874 | 725 | 6.21 |
| Zu-0 | 1 | 29213 | 1585 | 7366 | 19675 | 587 | 7.38 |
| Zu-0 | 2 | 29283 | 1638 | 8082 | 18923 | 640 | 7.34 |
| Zu-0 | 3 | 24548 | 1325 | 6549 | 16186 | 488 | 6.93 |
| Zu-0 | 4 | 4947 | 255 | 1416 | 3174 | 102 | 6.72 |

**Figure 3 – Source Data 3. *I2f* F_1_ flow cytometry count data.** cM were calculated as 100xR5/(R3+R5).

| Cross | Replicate | Total pollen | Red alone (Gate R2) | Red and Green (Gate R3) | Neither (Gate R4) | Green alone (Gate R5) | cM |
| --- | --- | --- | --- | --- | --- | --- | --- |
| Bay-0 | 1 | 40306 | 4455 | 15868 | 17996 | 1987 | 11.13 |
| Bay-0 | 2 | 39902 | 3803 | 16500 | 17623 | 1976 | 10.69 |
| Bay-0 | 3 | 39915 | 4604 | 14857 | 18426 | 2028 | 12.01 |
| Bur-0 | 1 | 50150 | 3696 | 12899 | 29841 | 3714 | 22.36 |
| Bur-0 | 2 | 50308 | 3604 | 13362 | 29721 | 3621 | 21.32 |
| Bur-0 | 3 | 50187 | 3568 | 10714 | 32111 | 3794 | 26.15 |
| Bur-0 | 4 | 50360 | 4066 | 14481 | 28642 | 3171 | 17.96 |
| C24 | 1 | 30043 | 1853 | 9237 | 16750 | 2203 | 19.26 |
| C24 | 2 | 30122 | 2616 | 9248 | 16399 | 1859 | 16.74 |
| C24 | 3 | 29843 | 1992 | 8504 | 16922 | 2425 | 22.19 |
| C24 | 4 | 30020 | 2211 | 10115 | 15806 | 1888 | 15.73 |
| Can-0 | 1 | 29636 | 1462 | 7753 | 18128 | 2293 | 22.83 |
| Can-0 | 2 | 29837 | 1709 | 8442 | 17380 | 2306 | 21.46 |
| Can-0 | 3 | 29883 | 1236 | 7513 | 19050 | 2084 | 21.72 |
| Can-0 | 4 | 29776 | 1505 | 8060 | 17872 | 2339 | 22.49 |
| Col-0 | 1 | 40668 | 2800 | 15893 | 20311 | 1664 | 9.48 |
| Col-0 | 2 | 40727 | 3044 | 16129 | 20073 | 1481 | 8.41 |
| Col-0 | 3 | 40469 | 2930 | 15877 | 20225 | 1437 | 8.3 |
| Col-0 | 4 | 29992 | 2071 | 12983 | 13702 | 1236 | 8.69 |
| Col-0 | 5 | 29961 | 1943 | 13364 | 13369 | 1285 | 8.77 |
| Col-0 | 6 | 30076 | 2119 | 12977 | 13670 | 1310 | 9.17 |
| Ct-1 | 1 | 30009 | 4353 | 10546 | 14273 | 837 | 7.35 |
| Ct-1 | 2 | 30073 | 4553 | 10220 | 14526 | 774 | 7.04 |
| Ct-1 | 3 | 40103 | 6102 | 13764 | 19140 | 1097 | 7.38 |
| Ct-1 | 4 | 40247 | 6784 | 12954 | 19530 | 979 | 7.03 |
| Cvi-0 | 1 | 41724 | 4272 | 16304 | 19261 | 1887 | 10.37 |
| Cvi-0 | 2 | 40844 | 3697 | 15132 | 20251 | 1764 | 10.44 |
| Cvi-0 | 3 | 40856 | 3808 | 14829 | 20258 | 1961 | 11.68 |
| Cvi-0 | 4 | 40981 | 3419 | 14610 | 20790 | 2162 | 12.89 |
| Edi-0 | 1 | 31509 | 2405 | 9848 | 17665 | 1591 | 13.91 |
| Edi-0 | 2 | 32767 | 3089 | 10003 | 18174 | 1501 | 13.05 |
| Edi-0 | 3 | 36687 | 3806 | 10554 | 20704 | 1623 | 13.33 |
| Edi-0 | 4 | 30636 | 2912 | 9048 | 17269 | 1407 | 13.46 |
| Hi-0 | 1 | 40392 | 6651 | 12128 | 20662 | 951 | 7.27 |
| Hi-0 | 2 | 40416 | 5472 | 14030 | 19850 | 1064 | 7.05 |
| Hi-0 | 3 | 40585 | 5797 | 14383 | 19423 | 982 | 6.39 |
| Kas | 1 | 29187 | 1930 | 9516 | 16329 | 1412 | 12.92 |
| Kas | 2 | 29137 | 1795 | 9455 | 16500 | 1387 | 12.79 |
| Kas | 3 | 29065 | 1994 | 7861 | 17805 | 1405 | 15.16 |
| Kas | 4 | 29688 | 2239 | 9054 | 16973 | 1422 | 13.57 |
| Kas | 5 | 39447 | 2740 | 11003 | 23765 | 1939 | 14.98 |
| Kas | 6 | 39667 | 2725 | 14254 | 20967 | 1721 | 10.77 |
| Kas | 7 | 40532 | 2844 | 13757 | 22024 | 1907 | 12.17 |
| Kn-0 | 1 | 28762 | 2701 | 12301 | 12620 | 1140 | 8.48 |
| Kn-0 | 2 | 29663 | 3210 | 11888 | 13571 | 994 | 7.72 |
| Kn-0 | 3 | 25980 | 2489 | 11782 | 10714 | 995 | 7.79 |
| Kond | 1 | 29473 | 1666 | 11126 | 14871 | 1810 | 13.99 |
| Kond | 2 | 29202 | 1647 | 9331 | 16189 | 2035 | 17.9 |
| Kond | 3 | 29386 | 1788 | 10183 | 15502 | 1913 | 15.82 |
| Kond | 4 | 29430 | 1939 | 9982 | 15653 | 1856 | 15.68 |
| Ler-0 | 1 | 40143 | 3119 | 18074 | 17487 | 1463 | 7.49 |
| Ler-0 | 2 | 40388 | 2982 | 17614 | 18258 | 1534 | 8.01 |
| Ler-0 | 3 | 40100 | 3524 | 17967 | 17188 | 1421 | 7.33 |
| Ler-0 | 4 | 39841 | 3487 | 17531 | 17379 | 1444 | 7.61 |
| Mt-0 | 1 | 30381 | 3157 | 9741 | 16071 | 1412 | 12.66 |
| Mt-0 | 2 | 30552 | 2968 | 10253 | 15651 | 1680 | 14.08 |
| Mt-0 | 3 | 40575 | 4349 | 13708 | 20510 | 2008 | 12.78 |
| No-0 | 1 | 30204 | 2220 | 12532 | 14572 | 880 | 6.56 |
| No-0 | 2 | 30235 | 2197 | 12906 | 14165 | 967 | 6.97 |
| No-0 | 3 | 30155 | 2361 | 12380 | 14533 | 881 | 6.64 |
| No-0 | 4 | 30417 | 3167 | 11815 | 14609 | 826 | 6.53 |
| Oy-0 | 1 | 40565 | 5070 | 14256 | 19989 | 1250 | 8.06 |
| Oy-0 | 2 | 40478 | 5743 | 13661 | 19800 | 1274 | 8.53 |
| Oy-0 | 3 | 30268 | 3713 | 11166 | 14318 | 1071 | 8.75 |
| Oy-0 | 4 | 30423 | 4314 | 9472 | 15732 | 905 | 8.72 |
| Po-0 | 1 | 31422 | 2646 | 12552 | 15025 | 1199 | 8.72 |
| Po-0 | 2 | 49618 | 4010 | 20163 | 23392 | 2053 | 9.24 |
| Po-0 | 3 | 30785 | 2379 | 12491 | 14619 | 1296 | 9.4 |
| Rsch-4 | 1 | 31104 | 2773 | 11273 | 15706 | 1352 | 10.71 |
| Rsch-4 | 2 | 30418 | 3124 | 10572 | 15246 | 1476 | 12.25 |
| Rsch-4 | 3 | 32833 | 3090 | 12523 | 15756 | 1464 | 10.47 |
| Rsch-4 | 4 | 50048 | 5174 | 18800 | 24148 | 1926 | 9.29 |
| Sf-2 | 1 | 29806 | 3731 | 10049 | 15212 | 814 | 7.49 |
| Sf-2 | 2 | 29860 | 3898 | 10163 | 14965 | 834 | 7.58 |
| Sf-2 | 3 | 23893 | 2935 | 8298 | 12053 | 607 | 6.82 |
| Sf-2 | 4 | 34836 | 4144 | 11724 | 17979 | 989 | 7.78 |
| Sha | 1 | 30287 | 2019 | 4677 | 22421 | 1170 | 20.01 |
| Sha | 2 | 30189 | 1748 | 3565 | 23850 | 1026 | 22.35 |
| Sha | 3 | 30341 | 2294 | 4280 | 22750 | 1017 | 19.2 |
| Sha | 4 | 30496 | 2416 | 4463 | 22606 | 1011 | 18.47 |
| Tsu-0 | 1 | 29704 | 2150 | 11911 | 14792 | 851 | 6.67 |
| Tsu-0 | 2 | 30166 | 2305 | 12381 | 14560 | 920 | 6.92 |
| Tsu-0 | 3 | 30022 | 2023 | 12919 | 14095 | 985 | 7.08 |
| Tsu-0 | 4 | 30044 | 2186 | 12754 | 14140 | 964 | 7.03 |
| Wil-2 | 1 | 30075 | 2485 | 12044 | 14712 | 834 | 6.48 |
| Wil-2 | 2 | 30068 | 2485 | 11984 | 14729 | 870 | 6.77 |
| Wil-2 | 3 | 29830 | 2712 | 11898 | 14533 | 687 | 5.46 |
| Wil-2 | 4 | 29982 | 2592 | 11843 | 14853 | 694 | 5.54 |
| Ws-0 | 1 | 19946 | 1948 | 7505 | 9643 | 850 | 10.17 |
| Ws-0 | 2 | 21733 | 2053 | 8053 | 10679 | 948 | 10.53 |
| Ws-0 | 3 | 21625 | 2240 | 7846 | 10674 | 865 | 9.93 |
| Ws-0 | 4 | 18210 | 2150 | 6368 | 8967 | 725 | 10.22 |
| Wu-0 | 1 | 30370 | 2628 | 10548 | 16136 | 1058 | 9.12 |
| Wu-0 | 2 | 30470 | 2675 | 10787 | 15843 | 1165 | 9.75 |
| Wu-0 | 3 | 30241 | 2627 | 11004 | 15475 | 1135 | 9.35 |
| Wu-0 | 4 | 30568 | 2812 | 10182 | 16476 | 1098 | 9.73 |
| Zu-0 | 1 | 28348 | 2690 | 6748 | 17794 | 1116 | 14.19 |
| Zu-0 | 2 | 28417 | 2790 | 7024 | 17562 | 1041 | 12.91 |
| Zu-0 | 3 | 28678 | 2832 | 7015 | 17798 | 1033 | 12.84 |
| Zu-0 | 4 | 28589 | 2784 | 6852 | 17875 | 1078 | 13.59 |

**Figure 3 – Source Data 4. *420* F_1_ fluorescent seed count data.** For the formula used for cM calculation please see Materials and Methods.

| Cross | Replicate | Green alone | Red alone | Red and Green | Neither | Total seed | cM | Green:Non-green | Red:  Non-red | Green alone:  Red alone |
| --- | --- | --- | --- | --- | --- | --- | --- | --- | --- | --- |
| Col-0 | 1 | 72 | 86 | 680 | 163 | 1001 | 17.28 | 3.02 | 3.26 | 0.84 |
| Col-0 | 2 | 96 | 107 | 734 | 178 | 1115 | 20.26 | 2.91 | 3.07 | 0.9 |
| Col-0 | 3 | 89 | 100 | 727 | 182 | 1098 | 19.02 | 2.89 | 3.05 | 0.89 |
| Col-0 | 4 | 173 | 223 | 1677 | 405 | 2478 | 17.51 | 2.95 | 3.29 | 0.78 |
| Col-0 | 5 | 190 | 242 | 1658 | 365 | 2455 | 19.5 | 3.04 | 3.42 | 0.79 |
| Col-0 | 6 | 138 | 166 | 1345 | 342 | 1991 | 16.66 | 2.92 | 3.15 | 0.83 |
| Col-0 | 7 | 234 | 253 | 1984 | 512 | 2983 | 17.93 | 2.9 | 3 | 0.92 |
| Col-0 | 8 | 217 | 240 | 1904 | 490 | 2851 | 17.57 | 2.91 | 3.03 | 0.9 |
| Col-0 | 9 | 232 | 220 | 1999 | 517 | 2968 | 16.61 | 3.03 | 2.96 | 1.05 |
| Ler-0 | 1 | 74 | 79 | 799 | 204 | 1156 | 14.25 | 3.08 | 3.16 | 0.94 |
| Ler-0 | 1 | 69 | 64 | 705 | 224 | 1062 | 13.42 | 2.69 | 2.62 | 1.08 |
| Ler-0 | 2 | 60 | 68 | 717 | 164 | 1009 | 13.61 | 3.35 | 3.5 | 0.88 |
| Ler-0 | 3 | 133 | 124 | 1806 | 484 | 2547 | 10.66 | 3.19 | 3.13 | 1.07 |
| Ler-0 | 4 | 122 | 133 | 1706 | 458 | 2419 | 11.16 | 3.09 | 3.17 | 0.92 |
| Ler-0 | 5 | 139 | 148 | 1845 | 535 | 2667 | 11.41 | 2.9 | 2.96 | 0.94 |
| Ler-0 | 6 | 133 | 136 | 1573 | 443 | 2285 | 12.56 | 2.95 | 2.97 | 0.98 |
| Ler-0 | 7 | 131 | 127 | 1688 | 485 | 2431 | 11.25 | 2.97 | 2.95 | 1.03 |
| Tsu-0 | 1 | 72 | 68 | 761 | 205 | 1106 | 13.58 | 3.05 | 2.99 | 1.06 |
| Tsu-0 | 2 | 96 | 101 | 936 | 294 | 1427 | 14.92 | 2.61 | 2.66 | 0.95 |
| Tsu-0 | 3 | 78 | 70 | 838 | 221 | 1207 | 13.12 | 3.15 | 3.04 | 1.11 |
| Tsu-0 | 4 | 209 | 208 | 1894 | 533 | 2844 | 15.93 | 2.84 | 2.83 | 1 |
| Tsu-0 | 5 | 202 | 153 | 1800 | 493 | 2648 | 14.45 | 3.1 | 2.81 | 1.32 |
| Tsu-0 | 6 | 165 | 168 | 1787 | 486 | 2606 | 13.72 | 2.98 | 3 | 0.98 |
| Tsu-0 | 7 | 187 | 176 | 1676 | 461 | 2500 | 15.76 | 2.92 | 2.86 | 1.06 |
| Tsu-0 | 8 | 179 | 161 | 1706 | 494 | 2540 | 14.43 | 2.88 | 2.77 | 1.11 |
| Ct-1 | 1 | 108 | 108 | 882 | 231 | 1329 | 17.85 | 2.92 | 2.92 | 1 |
| Ct-1 | 2 | 93 | 101 | 819 | 217 | 1230 | 17.26 | 2.87 | 2.97 | 0.92 |
| Ct-1 | 3 | 85 | 74 | 671 | 202 | 1032 | 16.82 | 2.74 | 2.6 | 1.15 |
| Ct-1 | 4 | 152 | 152 | 1483 | 405 | 2192 | 14.99 | 2.94 | 2.94 | 1 |
| Ct-1 | 5 | 174 | 146 | 1713 | 479 | 2512 | 13.67 | 3.02 | 2.85 | 1.19 |
| Ct-1 | 6 | 182 | 224 | 1670 | 436 | 2512 | 17.74 | 2.81 | 3.06 | 0.81 |
| Ct-1 | 7 | 181 | 218 | 1649 | 419 | 2467 | 17.75 | 2.87 | 3.11 | 0.83 |
| Ct-1 | 8 | 157 | 185 | 1563 | 447 | 2352 | 15.79 | 2.72 | 2.89 | 0.85 |
| Ct-1 | 9 | 134 | 163 | 1626 | 436 | 2359 | 13.5 | 2.94 | 3.14 | 0.82 |
| Ct-1 | 10 | 146 | 162 | 1685 | 459 | 2452 | 13.47 | 2.95 | 3.05 | 0.9 |
| Cvi-0 | 1 | 62 | 58 | 899 | 150 | 1169 | 10.85 | 4.62 | 4.51 | 1.07 |
| Cvi-0 | 2 | 58 | 61 | 949 | 253 | 1321 | 9.46 | 3.21 | 3.25 | 0.95 |
| Cvi-0 | 3 | 70 | 71 | 895 | 217 | 1253 | 11.97 | 3.35 | 3.37 | 0.99 |
| Cvi-0 | 4 | 141 | 165 | 1382 | 293 | 1981 | 16.87 | 3.33 | 3.56 | 0.85 |
| Cvi-0 | 5 | 162 | 155 | 1662 | 457 | 2436 | 13.99 | 2.98 | 2.94 | 1.05 |
| No-0 | 1 | 86 | 79 | 830 | 236 | 1231 | 14.45 | 2.91 | 2.82 | 1.09 |
| No-0 | 2 | 66 | 69 | 765 | 180 | 1080 | 13.4 | 3.34 | 3.39 | 0.96 |
| No-0 | 3 | 84 | 85 | 763 | 248 | 1180 | 15.53 | 2.54 | 2.55 | 0.99 |
| No-0 | 4 | 171 | 140 | 1743 | 474 | 2528 | 13.17 | 3.12 | 2.92 | 1.22 |
| No-0 | 5 | 161 | 153 | 1724 | 501 | 2539 | 13.24 | 2.88 | 2.84 | 1.05 |
| No-0 | 6 | 172 | 179 | 1841 | 505 | 2697 | 13.99 | 2.94 | 2.98 | 0.96 |
| No-0 | 7 | 165 | 158 | 1594 | 441 | 2358 | 14.79 | 2.94 | 2.89 | 1.04 |
| Po-0 | 1 | 78 | 73 | 844 | 227 | 1222 | 13.23 | 3.07 | 3.01 | 1.07 |
| Po-0 | 2 | 82 | 79 | 846 | 220 | 1227 | 14.12 | 3.1 | 3.06 | 1.04 |
| Po-0 | 3 | 166 | 157 | 1648 | 466 | 2437 | 14.27 | 2.91 | 2.86 | 1.06 |
| Po-0 | 4 | 191 | 197 | 1785 | 468 | 2641 | 15.97 | 2.97 | 3.01 | 0.97 |
| Po-0 | 5 | 198 | 189 | 1735 | 413 | 2535 | 16.65 | 3.21 | 3.15 | 1.05 |
| Po-0 | 6 | 219 | 188 | 1785 | 449 | 2641 | 16.83 | 3.15 | 2.95 | 1.16 |
| Po-0 | 7 | 215 | 229 | 1836 | 456 | 2736 | 17.81 | 2.99 | 3.08 | 0.94 |
| Po-0 | 8 | 201 | 192 | 1661 | 431 | 2485 | 17.31 | 2.99 | 2.93 | 1.05 |
| Rsch-4 | 1 | 83 | 66 | 800 | 195 | 1144 | 14.01 | 3.38 | 3.12 | 1.26 |
| Rsch-4 | 2 | 81 | 87 | 739 | 189 | 1096 | 16.73 | 2.97 | 3.06 | 0.93 |
| Rsch-4 | 3 | 89 | 92 | 839 | 218 | 1238 | 15.88 | 2.99 | 3.03 | 0.97 |
| Rsch-4 | 4 | 143 | 175 | 1625 | 442 | 2385 | 14.37 | 2.87 | 3.08 | 0.82 |
| Rsch-4 | 5 | 181 | 168 | 1734 | 434 | 2517 | 14.99 | 3.18 | 3.09 | 1.08 |
| Bur-0 | 1 | 66 | 73 | 798 | 186 | 1123 | 13.26 | 3.34 | 3.46 | 0.9 |
| Bur-0 | 2 | 59 | 60 | 736 | 185 | 1040 | 12.18 | 3.24 | 3.26 | 0.98 |
| Bur-0 | 3 | 68 | 59 | 793 | 196 | 1116 | 12.11 | 3.38 | 3.23 | 1.15 |
| Bur-0 | 4 | 160 | 152 | 1305 | 327 | 1944 | 17.6 | 3.06 | 2.99 | 1.05 |
| Bur-0 | 5 | 154 | 165 | 1527 | 365 | 2211 | 15.65 | 3.17 | 3.26 | 0.93 |
| Bur-0 | 6 | 158 | 203 | 1588 | 424 | 2373 | 16.59 | 2.78 | 3.08 | 0.78 |
| Bur-0 | 7 | 135 | 157 | 1568 | 376 | 2236 | 14.05 | 3.2 | 3.38 | 0.86 |
| Bur-0 | 8 | 163 | 154 | 1536 | 331 | 2184 | 15.76 | 3.5 | 3.42 | 1.06 |
| Wu-0 | 1 | 62 | 76 | 691 | 186 | 1015 | 14.67 | 2.87 | 3.09 | 0.82 |
| Wu-0 | 2 | 86 | 73 | 692 | 215 | 1066 | 16.23 | 2.7 | 2.54 | 1.18 |
| Wu-0 | 3 | 79 | 68 | 861 | 237 | 1245 | 12.6 | 3.08 | 2.94 | 1.16 |
| Wu-0 | 4 | 175 | 166 | 1659 | 449 | 2449 | 15.06 | 2.98 | 2.92 | 1.05 |
| Wu-0 | 5 | 170 | 150 | 1665 | 469 | 2454 | 14.02 | 2.96 | 2.84 | 1.13 |
| Wu-0 | 6 | 139 | 131 | 1779 | 531 | 2580 | 11.08 | 2.9 | 2.85 | 1.06 |
| Wu-0 | 7 | 160 | 160 | 1588 | 461 | 2369 | 14.57 | 2.81 | 2.81 | 1 |
| Hi-0 | 1 | 79 | 87 | 729 | 222 | 1117 | 16.17 | 2.61 | 2.71 | 0.91 |
| Hi-0 | 2 | 77 | 77 | 703 | 174 | 1031 | 16.26 | 3.11 | 3.11 | 1 |
| Hi-0 | 3 | 75 | 67 | 811 | 271 | 1224 | 12.37 | 2.62 | 2.54 | 1.12 |
| Hi-0 | 4 | 144 | 143 | 1716 | 422 | 2425 | 12.63 | 3.29 | 3.28 | 1.01 |
| Hi-0 | 5 | 163 | 140 | 1830 | 496 | 2629 | 12.28 | 3.13 | 2.99 | 1.16 |
| Hi-0 | 6 | 138 | 128 | 1668 | 470 | 2404 | 11.76 | 3.02 | 2.95 | 1.08 |
| Oy-0 | 1 | 98 | 81 | 875 | 204 | 1258 | 15.42 | 3.41 | 3.17 | 1.21 |
| Oy-0 | 2 | 97 | 78 | 790 | 210 | 1175 | 16.21 | 3.08 | 2.83 | 1.24 |
| Oy-0 | 3 | 108 | 109 | 956 | 261 | 1434 | 16.49 | 2.88 | 2.89 | 0.99 |
| Oy-0 | 4 | 171 | 181 | 1662 | 424 | 2438 | 15.67 | 3.03 | 3.1 | 0.94 |
| Oy-0 | 5 | 178 | 150 | 1643 | 455 | 2426 | 14.58 | 3.01 | 2.83 | 1.19 |
| Oy-0 | 6 | 167 | 188 | 1606 | 397 | 2358 | 16.4 | 3.03 | 3.18 | 0.89 |
| Oy-0 | 7 | 179 | 167 | 1600 | 401 | 2347 | 16.03 | 3.13 | 3.05 | 1.07 |
| Oy-0 | 8 | 167 | 165 | 1646 | 448 | 2426 | 14.78 | 2.96 | 2.94 | 1.01 |
| Kn-0 | 1 | 86 | 66 | 771 | 200 | 1123 | 14.6 | 3.22 | 2.93 | 1.3 |
| Kn-0 | 2 | 60 | 86 | 721 | 191 | 1058 | 14.91 | 2.82 | 3.22 | 0.7 |
| Kn-0 | 3 | 65 | 53 | 734 | 188 | 1040 | 12.08 | 3.32 | 3.11 | 1.23 |
| Kn-0 | 4 | 216 | 222 | 1938 | 531 | 2907 | 16.41 | 2.86 | 2.89 | 0.97 |
| Kn-0 | 5 | 194 | 266 | 1971 | 528 | 2959 | 16.99 | 2.73 | 3.1 | 0.73 |
| Kn-0 | 6 | 208 | 238 | 2011 | 529 | 2986 | 16.26 | 2.89 | 3.05 | 0.87 |
| Kn-0 | 7 | 214 | 181 | 1837 | 469 | 2701 | 15.89 | 3.16 | 2.95 | 1.18 |
| Kn-0 | 8 | 178 | 194 | 1730 | 438 | 2540 | 15.91 | 3.02 | 3.12 | 0.92 |
| Kn-0 | 9 | 192 | 227 | 1924 | 503 | 2846 | 16 | 2.9 | 3.09 | 0.85 |
| Sha | 1 | 76 | 61 | 1540 | 475 | 2152 | 6.58 | 3.01 | 2.91 | 1.25 |
| Sha | 2 | 70 | 65 | 1498 | 447 | 2080 | 6.19 | 3.06 | 3.02 | 1.08 |
| Sha | 3 | 65 | 80 | 1663 | 466 | 2274 | 6.59 | 3.16 | 3.28 | 0.81 |
| Sha | 4 | 64 | 78 | 1708 | 493 | 2343 | 6.26 | 3.1 | 3.21 | 0.82 |
| Sha | 5 | 83 | 89 | 1655 | 527 | 2354 | 7.6 | 2.82 | 2.86 | 0.93 |
| Sha | 6 | 83 | 94 | 1721 | 490 | 2388 | 7.71 | 3.09 | 3.17 | 0.88 |
| Sha | 7 | 82 | 72 | 1691 | 525 | 2370 | 6.72 | 2.97 | 2.9 | 1.14 |
| Sha | 8 | 89 | 87 | 1791 | 512 | 2479 | 7.37 | 3.14 | 3.12 | 1.02 |
| Sha | 9 | 79 | 83 | 1737 | 450 | 2349 | 7.15 | 3.41 | 3.44 | 0.95 |
| Sha | 10 | 75 | 90 | 1648 | 483 | 2296 | 7.47 | 3.01 | 3.11 | 0.83 |
| Sha | 11 | 79 | 88 | 1750 | 487 | 2404 | 7.21 | 3.18 | 3.25 | 0.9 |
| Bay-0 | 1 | 225 | 212 | 1682 | 418 | 2537 | 19.04 | 3.03 | 2.95 | 1.06 |
| Bay-0 | 2 | 155 | 163 | 1724 | 495 | 2537 | 13.44 | 2.86 | 2.9 | 0.95 |
| Bay-0 | 3 | 212 | 205 | 1717 | 416 | 2550 | 17.97 | 3.11 | 3.06 | 1.03 |
| Bay-0 | 4 | 156 | 160 | 1626 | 453 | 2395 | 14.2 | 2.91 | 2.93 | 0.98 |
| Bay-0 | 5 | 205 | 235 | 1568 | 416 | 2424 | 20.19 | 2.72 | 2.9 | 0.87 |
| Bay-0 | 6 | 139 | 126 | 777 | 201 | 1243 | 24.26 | 2.8 | 2.66 | 1.1 |
| Bay-0 | 7 | 147 | 159 | 1677 | 507 | 2490 | 13.15 | 2.74 | 2.81 | 0.92 |
| Bay-0 | 8 | 225 | 204 | 1680 | 421 | 2530 | 18.71 | 3.05 | 2.92 | 1.1 |
| Bay-0 | 9 | 270 | 242 | 1496 | 342 | 2350 | 24.88 | 3.02 | 2.84 | 1.12 |
| Bay-0 | 10 | 220 | 212 | 1757 | 409 | 2598 | 18.3 | 3.18 | 3.13 | 1.04 |
| Bay-0 | 11 | 198 | 189 | 1501 | 342 | 2230 | 19.2 | 3.2 | 3.13 | 1.05 |
| Bay-0 | 12 | 215 | 217 | 1640 | 433 | 2505 | 19.06 | 2.85 | 2.87 | 0.99 |
| Bay-0 | 13 | 187 | 204 | 1395 | 379 | 2165 | 19.2 | 2.71 | 2.83 | 0.92 |
| Kond | 1 | 155 | 133 | 1614 | 411 | 2313 | 13.34 | 3.25 | 3.09 | 1.17 |
| Kond | 2 | 137 | 163 | 1705 | 488 | 2493 | 12.86 | 2.83 | 2.99 | 0.84 |
| Kond | 3 | 138 | 167 | 1669 | 467 | 2441 | 13.39 | 2.85 | 3.03 | 0.83 |
| Kond | 4 | 136 | 147 | 1671 | 445 | 2399 | 12.59 | 3.05 | 3.13 | 0.93 |
| Kond | 5 | 202 | 164 | 1877 | 508 | 2751 | 14.33 | 3.09 | 2.87 | 1.23 |
| Kond | 6 | 149 | 187 | 1811 | 432 | 2579 | 14.01 | 3.17 | 3.44 | 0.8 |
| Kond | 7 | 199 | 146 | 1785 | 454 | 2584 | 14.39 | 3.31 | 2.96 | 1.36 |
| Kond | 8 | 165 | 194 | 1768 | 511 | 2638 | 14.69 | 2.74 | 2.9 | 0.85 |
| C24 | 1 | 134 | 160 | 1744 | 512 | 2550 | 12.28 | 2.79 | 2.95 | 0.84 |
| C24 | 2 | 136 | 124 | 1633 | 414 | 2307 | 11.99 | 3.29 | 3.19 | 1.1 |
| C24 | 3 | 157 | 138 | 1707 | 448 | 2450 | 12.87 | 3.18 | 3.05 | 1.14 |
| C24 | 4 | 149 | 147 | 1816 | 490 | 2602 | 12.11 | 3.08 | 3.07 | 1.01 |
| C24 | 5 | 127 | 127 | 1579 | 479 | 2312 | 11.67 | 2.82 | 2.82 | 1 |
| C24 | 6 | 132 | 105 | 1525 | 429 | 2191 | 11.48 | 3.1 | 2.91 | 1.26 |
| Kas | 1 | 162 | 132 | 1574 | 367 | 2235 | 14.16 | 3.48 | 3.22 | 1.23 |
| Kas | 2 | 133 | 141 | 1546 | 381 | 2201 | 13.34 | 3.22 | 3.28 | 0.94 |
| Kas | 3 | 107 | 142 | 1563 | 423 | 2235 | 11.84 | 2.96 | 3.22 | 0.75 |
| Kas | 4 | 174 | 121 | 1538 | 362 | 2195 | 14.49 | 3.54 | 3.1 | 1.44 |
| Kas | 5 | 150 | 140 | 1601 | 381 | 2272 | 13.7 | 3.36 | 3.28 | 1.07 |
| Kas | 6 | 115 | 100 | 1206 | 288 | 1709 | 13.49 | 3.4 | 3.24 | 1.15 |
| Kas | 7 | 119 | 135 | 1573 | 389 | 2216 | 12.21 | 3.23 | 3.36 | 0.88 |
| Kas | 8 | 54 | 64 | 526 | 122 | 766 | 16.82 | 3.12 | 3.35 | 0.84 |
| Co | 1 | 116 | 114 | 1447 | 395 | 2072 | 11.8 | 3.07 | 3.05 | 1.02 |
| Co | 2 | 115 | 99 | 1643 | 466 | 2323 | 9.68 | 3.11 | 3 | 1.16 |
| Co | 3 | 127 | 147 | 1693 | 462 | 2429 | 12 | 2.99 | 3.12 | 0.86 |
| Co | 4 | 138 | 123 | 1741 | 494 | 2496 | 11.07 | 3.05 | 2.95 | 1.12 |
| Can-0 | 1 | 119 | 181 | 1756 | 464 | 2520 | 12.71 | 2.91 | 3.32 | 0.66 |
| Can-0 | 2 | 130 | 163 | 1696 | 464 | 2453 | 12.76 | 2.91 | 3.13 | 0.8 |
| Can-0 | 3 | 158 | 154 | 1897 | 524 | 2733 | 12.15 | 3.03 | 3.01 | 1.03 |
| Can-0 | 4 | 159 | 146 | 1686 | 457 | 2448 | 13.35 | 3.06 | 2.97 | 1.09 |
| Can-0 | 5 | 116 | 123 | 1486 | 460 | 2185 | 11.61 | 2.75 | 2.79 | 0.94 |
| Can-0 | 6 | 129 | 153 | 1701 | 466 | 2449 | 12.27 | 2.96 | 3.12 | 0.84 |
| Can-0 | 7 | 181 | 113 | 1776 | 480 | 2550 | 12.28 | 3.3 | 2.86 | 1.6 |
| Can-0 | 8 | 154 | 124 | 1553 | 390 | 2221 | 13.42 | 3.32 | 3.08 | 1.24 |
| Can-0 | 9 | 133 | 145 | 1808 | 562 | 2648 | 11.12 | 2.75 | 2.81 | 0.92 |
| Sf-2 | 1 | 169 | 164 | 1539 | 333 | 2205 | 16.46 | 3.44 | 3.39 | 1.03 |
| Sf-2 | 2 | 195 | 195 | 1551 | 393 | 2334 | 18.4 | 2.97 | 2.97 | 1 |
| Sf-2 | 3 | 221 | 194 | 1441 | 372 | 2228 | 20.79 | 2.94 | 2.76 | 1.14 |
| Sf-2 | 4 | 187 | 193 | 1523 | 354 | 2257 | 18.56 | 3.13 | 3.17 | 0.97 |
| Sf-2 | 5 | 205 | 194 | 1551 | 393 | 2343 | 18.8 | 2.99 | 2.92 | 1.06 |
| Wil-2 | 1 | 173 | 156 | 1398 | 354 | 2081 | 17.31 | 3.08 | 2.95 | 1.11 |
| Wil-2 | 2 | 101 | 113 | 1128 | 292 | 1634 | 14.09 | 3.03 | 3.16 | 0.89 |
| Wil-2 | 3 | 191 | 166 | 1600 | 411 | 2368 | 16.42 | 3.1 | 2.93 | 1.15 |
| Wil-2 | 4 | 169 | 153 | 1510 | 443 | 2275 | 15.33 | 2.82 | 2.72 | 1.1 |
| Wil-2 | 5 | 139 | 148 | 1276 | 327 | 1890 | 16.56 | 2.98 | 3.06 | 0.94 |
| Mh-0 | 1 | 197 | 156 | 1767 | 472 | 2592 | 14.7 | 3.13 | 2.87 | 1.26 |
| Mh-0 | 2 | 194 | 174 | 1777 | 444 | 2589 | 15.4 | 3.19 | 3.06 | 1.11 |
| Mh-0 | 3 | 189 | 174 | 1743 | 503 | 2609 | 15.05 | 2.85 | 2.77 | 1.09 |
| Mh-0 | 4 | 194 | 187 | 1775 | 496 | 2652 | 15.58 | 2.88 | 2.84 | 1.04 |
| Mh-0 | 5 | 174 | 159 | 1760 | 509 | 2602 | 13.74 | 2.9 | 2.81 | 1.09 |
| Nw-0 | 1 | 176 | 149 | 1711 | 490 | 2526 | 13.82 | 2.95 | 2.79 | 1.18 |
| Nw-0 | 2 | 169 | 186 | 1792 | 471 | 2618 | 14.63 | 2.98 | 3.09 | 0.91 |
| Nw-0 | 3 | 169 | 158 | 1758 | 469 | 2554 | 13.75 | 3.07 | 3 | 1.07 |
| Nw-0 | 4 | 203 | 182 | 1723 | 454 | 2562 | 16.37 | 3.03 | 2.9 | 1.12 |
| Nw-0 | 5 | 198 | 170 | 1765 | 521 | 2654 | 14.99 | 2.84 | 2.69 | 1.16 |
| RRS7 | 1 | 153 | 150 | 1415 | 353 | 2071 | 15.89 | 3.12 | 3.09 | 1.02 |
| RRS7 | 2 | 215 | 147 | 1679 | 344 | 2385 | 16.55 | 3.86 | 3.27 | 1.46 |
| RRS7 | 3 | 245 | 145 | 1654 | 365 | 2409 | 17.77 | 3.72 | 2.95 | 1.69 |
| RRS7 | 4 | 237 | 164 | 1597 | 354 | 2352 | 18.82 | 3.54 | 2.98 | 1.45 |
| RRS7 | 5 | 214 | 162 | 1669 | 355 | 2400 | 17.13 | 3.64 | 3.22 | 1.32 |
| CIBC5 | 1 | 138 | 123 | 1748 | 473 | 2482 | 11.14 | 3.16 | 3.06 | 1.12 |
| CIBC5 | 2 | 178 | 155 | 1630 | 426 | 2389 | 15.08 | 3.11 | 2.96 | 1.15 |
| CIBC5 | 3 | 183 | 146 | 1710 | 473 | 2512 | 14.09 | 3.06 | 2.83 | 1.25 |
| CIBC5 | 4 | 128 | 124 | 1442 | 342 | 2036 | 13.26 | 3.37 | 3.33 | 1.03 |
| CIBC5 | 5 | 140 | 127 | 1630 | 436 | 2333 | 12.19 | 3.14 | 3.05 | 1.1 |
| Wl-0 | 1 | 192 | 202 | 1742 | 456 | 2592 | 16.57 | 2.94 | 3 | 0.95 |
| Wl-0 | 2 | 228 | 197 | 1711 | 418 | 2554 | 18.32 | 3.15 | 2.95 | 1.16 |
| Wl-0 | 3 | 178 | 183 | 1651 | 424 | 2436 | 16.12 | 3.01 | 3.05 | 0.97 |
| Wl-0 | 4 | 208 | 168 | 1589 | 413 | 2378 | 17.31 | 3.09 | 2.83 | 1.24 |
| Wl-0 | 5 | 188 | 191 | 1626 | 420 | 2425 | 17.09 | 2.97 | 2.99 | 0.98 |
| Wl-0 | 6 | 167 | 204 | 1634 | 437 | 2442 | 16.56 | 2.81 | 3.04 | 0.82 |
| Ws-0 | 1 | 149 | 151 | 1701 | 421 | 2422 | 13.27 | 3.23 | 3.25 | 0.99 |
| Ws-0 | 2 | 152 | 162 | 1843 | 450 | 2607 | 12.87 | 3.26 | 3.33 | 0.94 |
| Ws-0 | 3 | 160 | 141 | 1717 | 451 | 2469 | 13.04 | 3.17 | 3.04 | 1.13 |
| Ws-0 | 4 | 170 | 133 | 1833 | 477 | 2613 | 12.36 | 3.28 | 3.04 | 1.28 |
| Ws-0 | 5 | 140 | 134 | 1675 | 439 | 2388 | 12.22 | 3.17 | 3.12 | 1.04 |
| Ws-0 | 6 | 157 | 151 | 1639 | 444 | 2391 | 13.84 | 3.02 | 2.98 | 1.04 |
| Ws-0 | 7 | 159 | 170 | 1766 | 499 | 2594 | 13.61 | 2.88 | 2.94 | 0.94 |
| Bu-0 | 1 | 188 | 211 | 981 | 204 | 1584 | 29.56 | 2.82 | 3.04 | 0.89 |
| Bu-0 | 2 | 219 | 183 | 865 | 245 | 1512 | 31.57 | 2.53 | 2.26 | 1.2 |
| Bu-0 | 3 | 217 | 141 | 867 | 218 | 1443 | 29.02 | 3.02 | 2.32 | 1.54 |
| Bu-0 | 4 | 223 | 157 | 870 | 291 | 1541 | 28.81 | 2.44 | 2 | 1.42 |
| Bu-0 | 5 | 176 | 209 | 909 | 343 | 1637 | 27.22 | 1.97 | 2.15 | 0.84 |
| Bu-0 | 6 | 187 | 193 | 927 | 299 | 1606 | 27.42 | 2.26 | 2.3 | 0.97 |
| Bu-0 | 7 | 184 | 195 | 870 | 284 | 1533 | 28.9 | 2.2 | 2.28 | 0.94 |
| Mt-0 | 1 | 259 | 265 | 1710 | 465 | 2699 | 21.79 | 2.7 | 2.73 | 0.98 |
| Mt-0 | 2 | 213 | 248 | 1739 | 436 | 2636 | 19.36 | 2.85 | 3.06 | 0.86 |
| Mt-0 | 3 | 236 | 256 | 1678 | 417 | 2587 | 21.28 | 2.84 | 2.96 | 0.92 |
| Mt-0 | 4 | 214 | 253 | 1591 | 449 | 2507 | 20.79 | 2.57 | 2.78 | 0.85 |
| Mt-0 | 5 | 239 | 197 | 1683 | 456 | 2575 | 18.68 | 2.94 | 2.71 | 1.21 |
| Mt-0 | 6 | 224 | 230 | 1511 | 419 | 2384 | 21.32 | 2.67 | 2.71 | 0.97 |
| Mt-0 | 7 | 240 | 254 | 1680 | 440 | 2614 | 21.13 | 2.77 | 2.84 | 0.94 |
| Edi-0 | 1 | 152 | 150 | 1650 | 494 | 2446 | 13.22 | 2.8 | 2.79 | 1.01 |
| Edi-0 | 2 | 120 | 154 | 1420 | 398 | 2092 | 14.09 | 2.79 | 3.04 | 0.78 |
| Edi-0 | 3 | 124 | 136 | 1621 | 460 | 2341 | 11.8 | 2.93 | 3.01 | 0.91 |
| Edi-0 | 4 | 155 | 156 | 1587 | 408 | 2306 | 14.54 | 3.09 | 3.1 | 0.99 |
| Edi-0 | 5 | 145 | 145 | 1691 | 456 | 2437 | 12.71 | 3.05 | 3.05 | 1 |
| Zu-0 | 1 | 152 | 142 | 1626 | 483 | 2403 | 13.09 | 2.84 | 2.78 | 1.07 |
| Zu-0 | 2 | 164 | 129 | 1585 | 472 | 2350 | 13.36 | 2.91 | 2.69 | 1.27 |
| Zu-0 | 3 | 137 | 104 | 1661 | 499 | 2401 | 10.6 | 2.98 | 2.78 | 1.32 |
| Zu-0 | 4 | 156 | 141 | 1813 | 501 | 2611 | 12.11 | 3.07 | 2.97 | 1.11 |
| Zu-0 | 5 | 159 | 123 | 1737 | 507 | 2526 | 11.87 | 3.01 | 2.79 | 1.29 |

**Figure 4 – Source Data 5. *CEN3* F_1_ flow cytometry count data.** cM were calculated as 100 x R5/(R3+R5).

| Cross | Replicate | Total pollen | Red alone (Gate R2) | Red and green (Gate R3) | Neither  (Gate R4) | Green alone (Gate R5) | cM |
| --- | --- | --- | --- | --- | --- | --- | --- |
| Cvi-0 | 1 | 30066 | 3493 | 9235 | 13811 | 3527 | 27.64 |
| Cvi-0 | 2 | 30064 | 3100 | 9350 | 13529 | 4085 | 30.41 |
| Cvi-0 | 3 | 30086 | 3632 | 10192 | 12947 | 3315 | 24.54 |
| Cvi-0 | 4 | 29039 | 4232 | 7228 | 14942 | 2637 | 26.73 |
| Cvi-0 | 5 | 29128 | 4306 | 7034 | 15104 | 2684 | 27.62 |
| Cvi-0 | 6 | 28931 | 4511 | 7275 | 14763 | 2382 | 24.67 |
| Cvi-0 | 7 | 29137 | 4338 | 6842 | 14841 | 3116 | 31.29 |
| Wu-0 | 1 | 30095 | 3232 | 9742 | 15920 | 1201 | 10.98 |
| Wu-0 | 2 | 30101 | 3255 | 9312 | 16327 | 1207 | 11.47 |
| Wu-0 | 3 | 30078 | 3211 | 10567 | 14886 | 1414 | 11.8 |
| Hi-0 | 1 | 30455 | 2704 | 11262 | 15223 | 1266 | 10.11 |
| Hi-0 | 2 | 30255 | 2768 | 11118 | 15202 | 1167 | 9.5 |
| Hi-0 | 3 | 30229 | 2767 | 11156 | 15173 | 1133 | 9.22 |
| Ct-1 | 1 | 30281 | 3996 | 9627 | 15265 | 1393 | 12.64 |
| Ct-1 | 2 | 30199 | 3746 | 9215 | 16041 | 1197 | 11.5 |
| Ct-1 | 3 | 30246 | 3645 | 9398 | 15898 | 1305 | 12.19 |
| Bay-0 | 1 | 30162 | 3042 | 11195 | 14492 | 1433 | 11.35 |
| Bay-0 | 2 | 30214 | 3515 | 10640 | 14698 | 1361 | 11.34 |
| Bay-0 | 3 | 23855 | 1918 | 9514 | 11156 | 1267 | 11.75 |
| Ws-0 | 1 | 25135 | 2364 | 9052 | 12908 | 811 | 8.22 |
| Ws-0 | 2 | 31658 | 4003 | 8788 | 18032 | 835 | 8.68 |
| Ws-0 | 3 | 29812 | 4981 | 5291 | 18954 | 586 | 9.97 |
| Ler-0 | 1 | 30930 | 3312 | 10592 | 15505 | 1521 | 12.56 |
| Ler-0 | 2 | 30594 | 3062 | 11202 | 14843 | 1487 | 11.72 |
| Ler-0 | 3 | 30751 | 3014 | 11564 | 14673 | 1500 | 11.48 |
| Rsch-4 | 1 | 29606 | 3053 | 9662 | 15584 | 1307 | 11.92 |
| Rsch-4 | 2 | 29274 | 2847 | 9752 | 15296 | 1379 | 12.39 |
| Rsch-4 | 3 | 29603 | 2861 | 10320 | 14877 | 1545 | 13.02 |
| Tsu-0 | 1 | 29515 | 2765 | 10066 | 15653 | 1031 | 9.29 |
| Tsu-0 | 2 | 29375 | 3294 | 8445 | 16787 | 849 | 9.13 |
| Tsu-0 | 3 | 29919 | 3041 | 9331 | 16524 | 1023 | 9.88 |
| Col-0 | 1 | 29755 | 2126 | 12073 | 13978 | 1578 | 11.56 |
| Col-0 | 2 | 29791 | 2091 | 12163 | 13847 | 1690 | 12.2 |
| Col-0 | 3 | 29628 | 2056 | 12475 | 13509 | 1588 | 11.29 |
| Col-0 | 4 | 30316 | 2685 | 12687 | 13459 | 1485 | 10.48 |
| Col-0 | 5 | 30175 | 2277 | 13039 | 13200 | 1659 | 11.29 |
| Col-0 | 6 | 30350 | 2841 | 11557 | 14339 | 1613 | 12.25 |
| C24 | 1 | 30581 | 2772 | 11446 | 14462 | 1901 | 14.24 |
| C24 | 2 | 29127 | 2474 | 10992 | 13937 | 1724 | 13.56 |
| C24 | 3 | 29218 | 2737 | 10575 | 14101 | 1805 | 14.58 |
| Co | 1 | 29227 | 2943 | 10437 | 14238 | 1609 | 13.36 |
| Co | 2 | 29094 | 3486 | 9647 | 14396 | 1565 | 13.96 |
| Co | 3 | 29124 | 3868 | 8813 | 15016 | 1427 | 13.94 |
| Bur-0 | 1 | 30597 | 3065 | 9874 | 15606 | 2052 | 17.21 |
| Bur-0 | 2 | 30641 | 3404 | 9941 | 15373 | 1923 | 16.21 |
| Bur-0 | 3 | 31051 | 4124 | 8464 | 16534 | 1929 | 18.56 |
| Bur-0 | 4 | 29129 | 3193 | 7844 | 16216 | 1876 | 19.3 |
| Po-0 | 1 | 29418 | 3302 | 8464 | 16562 | 1090 | 11.41 |
| Po-0 | 2 | 29412 | 3166 | 8890 | 16452 | 904 | 9.23 |
| Po-0 | 3 | 29175 | 3340 | 8780 | 15835 | 1220 | 12.2 |
| Wil-2 | 1 | 29602 | 3296 | 9852 | 15441 | 1013 | 9.32 |
| Wil-2 | 2 | 29512 | 3502 | 8493 | 16593 | 924 | 9.81 |
| Wil-2 | 3 | 29346 | 3188 | 8661 | 16639 | 858 | 9.01 |
| Wil-2 | 4 | 29359 | 3564 | 8516 | 16103 | 1176 | 12.13 |
| No-0 | 1 | 29872 | 5567 | 3241 | 20643 | 421 | 11.5 |
| No-0 | 2 | 29840 | 5448 | 2866 | 21173 | 353 | 10.97 |
| No-0 | 3 | 29852 | 5555 | 3753 | 20047 | 497 | 11.69 |
| Sha | 1 | 29516 | 5133 | 2336 | 21498 | 549 | 19.03 |
| Sha | 2 | 29739 | 5079 | 2833 | 21211 | 616 | 17.86 |
| Sha | 3 | 29651 | 4821 | 2607 | 21620 | 603 | 18.79 |
| Kond | 1 | 30076 | 3140 | 11148 | 14431 | 1357 | 10.85 |
| Kond | 2 | 30090 | 3536 | 10194 | 14914 | 1446 | 12.42 |
| Kond | 3 | 30077 | 5773 | 8325 | 14950 | 1029 | 11 |
| Kn-0 | 1 | 30438 | 3052 | 10418 | 16032 | 936 | 8.24 |
| Kn-0 | 2 | 30138 | 2903 | 10844 | 15360 | 1031 | 8.68 |
| Kn-0 | 3 | 30233 | 3005 | 10769 | 15361 | 1098 | 9.25 |
| Can-0 | 1 | 41370 | 4211 | 10604 | 22362 | 4193 | 28.34 |
| Can-0 | 2 | 41137 | 3709 | 10624 | 22105 | 4699 | 30.67 |
| Can-0 | 3 | 41294 | 3707 | 8724 | 24144 | 4719 | 35.1 |
| Mt-0 | 1 | 49834 | 3875 | 20260 | 23504 | 2195 | 9.78 |
| Mt-0 | 2 | 30742 | 2684 | 12081 | 14786 | 1191 | 8.97 |
| Mt-0 | 3 | 49841 | 4259 | 19334 | 24207 | 2041 | 9.55 |
| Mt-0 | 4 | 49758 | 4306 | 18556 | 24836 | 2060 | 9.99 |
| Edi-0 | 1 | 30982 | 3029 | 12161 | 13898 | 1894 | 13.48 |
| Edi-0 | 2 | 30181 | 3128 | 10949 | 14328 | 1776 | 13.96 |
| Edi-0 | 3 | 32225 | 3211 | 12155 | 14951 | 1908 | 13.57 |
| Edi-0 | 4 | 35467 | 3925 | 12434 | 17157 | 1951 | 13.56 |
| Oy-0 | 1 | 30405 | 2914 | 12033 | 13817 | 1641 | 12 |
| Oy-0 | 2 | 30579 | 2930 | 11893 | 14010 | 1746 | 12.8 |
| Oy-0 | 3 | 30608 | 2740 | 12191 | 13914 | 1763 | 12.63 |
| Bu-0 | 1 | 21103 | 2977 | 4449 | 13251 | 426 | 8.74 |
| Bu-0 | 2 | 21101 | 3150 | 4040 | 13539 | 372 | 8.43 |
| Bu-0 | 3 | 21054 | 2852 | 4200 | 13572 | 430 | 9.29 |
| Bu-0 | 4 | 29852 | 4197 | 5619 | 19486 | 550 | 8.92 |
| CIBC5 | 1 | 29489 | 3090 | 11048 | 13995 | 1356 | 10.93 |
| CIBC5 | 2 | 29850 | 2557 | 11675 | 14119 | 1499 | 11.38 |
| CIBC5 | 3 | 29702 | 2727 | 10758 | 14778 | 1439 | 11.8 |
| CIBC5 | 4 | 29709 | 2748 | 11362 | 14180 | 1419 | 11.1 |
| Mh-0 | 1 | 30063 | 2541 | 11952 | 14275 | 1295 | 9.78 |
| Mh-0 | 2 | 30051 | 2478 | 12209 | 13990 | 1374 | 10.12 |
| Mh-0 | 3 | 30006 | 2582 | 11576 | 14568 | 1280 | 9.96 |
| Mh-0 | 4 | 30054 | 2524 | 11896 | 14241 | 1393 | 10.48 |
| Nw-0 | 1 | 30136 | 3543 | 11576 | 13096 | 1921 | 14.23 |
| Nw-0 | 2 | 30015 | 3384 | 11141 | 13454 | 2036 | 15.45 |
| Nw-0 | 3 | 29947 | 3023 | 11560 | 13504 | 1860 | 13.86 |
| Nw-0 | 4 | 30057 | 3100 | 11657 | 13377 | 1923 | 14.16 |
| Wl-0 | 1 | 30094 | 2605 | 11622 | 14722 | 1145 | 8.97 |
| Wl-0 | 2 | 30038 | 2630 | 11387 | 14843 | 1178 | 9.38 |
| Wl-0 | 3 | 30060 | 2584 | 11710 | 14473 | 1293 | 9.94 |
| Wl-0 | 4 | 30009 | 2653 | 11433 | 14717 | 1206 | 9.54 |
| RRS7 | 1 | 30093 | 2705 | 10975 | 14857 | 1556 | 12.42 |
| RRS7 | 2 | 29975 | 2171 | 12289 | 14036 | 1479 | 10.74 |
| RRS7 | 3 | 30079 | 2183 | 11025 | 15278 | 1593 | 12.62 |
| RRS7 | 4 | 29962 | 2122 | 11379 | 15058 | 1403 | 10.98 |
| Zu-0 | 1 | 29918 | 2560 | 9742 | 16490 | 1126 | 10.36 |
| Zu-0 | 2 | 29834 | 2652 | 8634 | 17586 | 962 | 10.03 |
| Zu-0 | 3 | 29763 | 2725 | 8892 | 17227 | 919 | 9.37 |
| Kas | 1 | 20462 | 1561 | 8143 | 9469 | 1289 | 13.67 |
| Kas | 2 | 40581 | 3077 | 3967 | 32920 | 617 | 13.46 |
| Kas | 3 | 50469 | 3478 | 4862 | 41363 | 766 | 13.61 |
| Kas | 4 | 50350 | 3781 | 8513 | 36836 | 1220 | 12.53 |
| Sf-2 | 1 | 31325 | 4464 | 9642 | 15766 | 1453 | 13.1 |
| Sf-2 | 2 | 29774 | 3674 | 9671 | 15126 | 1303 | 11.87 |
| Sf-2 | 3 | 30689 | 3822 | 9891 | 15636 | 1340 | 11.93 |
