# Supplementary material for: Juxtaposition of heterozygous and homozygous regions causes reciprocal crossover remodelling via interference during Arabidopsis meiosis
Source: eLife. 2015 Mar 27;4:e03708. doi: 10.7554/eLife.03708 (PMC4407271; doi:10.7554/eLife.03708)
Supplement: Figure 3—source data 5. — DOI: http://dx.doi.org/10.7554/eLife.03708.015 [file elife03708s006.docx]

**Figure 3 – Source Data 5. *CEN3* F_1_ flow cytometry count data.** cM were calculated as 100 x R5/(R3+R5).

| Cross | Replicate | Total pollen | Red alone (Gate R2) | Red and green (Gate R3) | Neither  (Gate R4) | Green alone (Gate R5) | cM |
| --- | --- | --- | --- | --- | --- | --- | --- |
| Cvi-0 | 1 | 30066 | 3493 | 9235 | 13811 | 3527 | 27.64 |
| Cvi-0 | 2 | 30064 | 3100 | 9350 | 13529 | 4085 | 30.41 |
| Cvi-0 | 3 | 30086 | 3632 | 10192 | 12947 | 3315 | 24.54 |
| Cvi-0 | 4 | 29039 | 4232 | 7228 | 14942 | 2637 | 26.73 |
| Cvi-0 | 5 | 29128 | 4306 | 7034 | 15104 | 2684 | 27.62 |
| Cvi-0 | 6 | 28931 | 4511 | 7275 | 14763 | 2382 | 24.67 |
| Cvi-0 | 7 | 29137 | 4338 | 6842 | 14841 | 3116 | 31.29 |
| Wu-0 | 1 | 30095 | 3232 | 9742 | 15920 | 1201 | 10.98 |
| Wu-0 | 2 | 30101 | 3255 | 9312 | 16327 | 1207 | 11.47 |
| Wu-0 | 3 | 30078 | 3211 | 10567 | 14886 | 1414 | 11.8 |
| Hi-0 | 1 | 30455 | 2704 | 11262 | 15223 | 1266 | 10.11 |
| Hi-0 | 2 | 30255 | 2768 | 11118 | 15202 | 1167 | 9.5 |
| Hi-0 | 3 | 30229 | 2767 | 11156 | 15173 | 1133 | 9.22 |
| Ct-1 | 1 | 30281 | 3996 | 9627 | 15265 | 1393 | 12.64 |
| Ct-1 | 2 | 30199 | 3746 | 9215 | 16041 | 1197 | 11.5 |
| Ct-1 | 3 | 30246 | 3645 | 9398 | 15898 | 1305 | 12.19 |
| Bay-0 | 1 | 30162 | 3042 | 11195 | 14492 | 1433 | 11.35 |
| Bay-0 | 2 | 30214 | 3515 | 10640 | 14698 | 1361 | 11.34 |
| Bay-0 | 3 | 23855 | 1918 | 9514 | 11156 | 1267 | 11.75 |
| Ws-0 | 1 | 25135 | 2364 | 9052 | 12908 | 811 | 8.22 |
| Ws-0 | 2 | 31658 | 4003 | 8788 | 18032 | 835 | 8.68 |
| Ws-0 | 3 | 29812 | 4981 | 5291 | 18954 | 586 | 9.97 |
| Ler-0 | 1 | 30930 | 3312 | 10592 | 15505 | 1521 | 12.56 |
| Ler-0 | 2 | 30594 | 3062 | 11202 | 14843 | 1487 | 11.72 |
| Ler-0 | 3 | 30751 | 3014 | 11564 | 14673 | 1500 | 11.48 |
| Rsch-4 | 1 | 29606 | 3053 | 9662 | 15584 | 1307 | 11.92 |
| Rsch-4 | 2 | 29274 | 2847 | 9752 | 15296 | 1379 | 12.39 |
| Rsch-4 | 3 | 29603 | 2861 | 10320 | 14877 | 1545 | 13.02 |
| Tsu-0 | 1 | 29515 | 2765 | 10066 | 15653 | 1031 | 9.29 |
| Tsu-0 | 2 | 29375 | 3294 | 8445 | 16787 | 849 | 9.13 |
| Tsu-0 | 3 | 29919 | 3041 | 9331 | 16524 | 1023 | 9.88 |
| Col-0 | 1 | 29755 | 2126 | 12073 | 13978 | 1578 | 11.56 |
| Col-0 | 2 | 29791 | 2091 | 12163 | 13847 | 1690 | 12.2 |
| Col-0 | 3 | 29628 | 2056 | 12475 | 13509 | 1588 | 11.29 |
| Col-0 | 4 | 30316 | 2685 | 12687 | 13459 | 1485 | 10.48 |
| Col-0 | 5 | 30175 | 2277 | 13039 | 13200 | 1659 | 11.29 |
| Col-0 | 6 | 30350 | 2841 | 11557 | 14339 | 1613 | 12.25 |
| C24 | 1 | 30581 | 2772 | 11446 | 14462 | 1901 | 14.24 |
| C24 | 2 | 29127 | 2474 | 10992 | 13937 | 1724 | 13.56 |
| C24 | 3 | 29218 | 2737 | 10575 | 14101 | 1805 | 14.58 |
| Co | 1 | 29227 | 2943 | 10437 | 14238 | 1609 | 13.36 |
| Co | 2 | 29094 | 3486 | 9647 | 14396 | 1565 | 13.96 |
| Co | 3 | 29124 | 3868 | 8813 | 15016 | 1427 | 13.94 |
| Bur-0 | 1 | 30597 | 3065 | 9874 | 15606 | 2052 | 17.21 |
| Bur-0 | 2 | 30641 | 3404 | 9941 | 15373 | 1923 | 16.21 |
| Bur-0 | 3 | 31051 | 4124 | 8464 | 16534 | 1929 | 18.56 |
| Bur-0 | 4 | 29129 | 3193 | 7844 | 16216 | 1876 | 19.3 |
| Po-0 | 1 | 29418 | 3302 | 8464 | 16562 | 1090 | 11.41 |
| Po-0 | 2 | 29412 | 3166 | 8890 | 16452 | 904 | 9.23 |
| Po-0 | 3 | 29175 | 3340 | 8780 | 15835 | 1220 | 12.2 |
| Wil-2 | 1 | 29602 | 3296 | 9852 | 15441 | 1013 | 9.32 |
| Wil-2 | 2 | 29512 | 3502 | 8493 | 16593 | 924 | 9.81 |
| Wil-2 | 3 | 29346 | 3188 | 8661 | 16639 | 858 | 9.01 |
| Wil-2 | 4 | 29359 | 3564 | 8516 | 16103 | 1176 | 12.13 |
| No-0 | 1 | 29872 | 5567 | 3241 | 20643 | 421 | 11.5 |
| No-0 | 2 | 29840 | 5448 | 2866 | 21173 | 353 | 10.97 |
| No-0 | 3 | 29852 | 5555 | 3753 | 20047 | 497 | 11.69 |
| Sha | 1 | 29516 | 5133 | 2336 | 21498 | 549 | 19.03 |
| Sha | 2 | 29739 | 5079 | 2833 | 21211 | 616 | 17.86 |
| Sha | 3 | 29651 | 4821 | 2607 | 21620 | 603 | 18.79 |
| Kond | 1 | 30076 | 3140 | 11148 | 14431 | 1357 | 10.85 |
| Kond | 2 | 30090 | 3536 | 10194 | 14914 | 1446 | 12.42 |
| Kond | 3 | 30077 | 5773 | 8325 | 14950 | 1029 | 11 |
| Kn-0 | 1 | 30438 | 3052 | 10418 | 16032 | 936 | 8.24 |
| Kn-0 | 2 | 30138 | 2903 | 10844 | 15360 | 1031 | 8.68 |
| Kn-0 | 3 | 30233 | 3005 | 10769 | 15361 | 1098 | 9.25 |
| Can-0 | 1 | 41370 | 4211 | 10604 | 22362 | 4193 | 28.34 |
| Can-0 | 2 | 41137 | 3709 | 10624 | 22105 | 4699 | 30.67 |
| Can-0 | 3 | 41294 | 3707 | 8724 | 24144 | 4719 | 35.1 |
| Mt-0 | 1 | 49834 | 3875 | 20260 | 23504 | 2195 | 9.78 |
| Mt-0 | 2 | 30742 | 2684 | 12081 | 14786 | 1191 | 8.97 |
| Mt-0 | 3 | 49841 | 4259 | 19334 | 24207 | 2041 | 9.55 |
| Mt-0 | 4 | 49758 | 4306 | 18556 | 24836 | 2060 | 9.99 |
| Edi-0 | 1 | 30982 | 3029 | 12161 | 13898 | 1894 | 13.48 |
| Edi-0 | 2 | 30181 | 3128 | 10949 | 14328 | 1776 | 13.96 |
| Edi-0 | 3 | 32225 | 3211 | 12155 | 14951 | 1908 | 13.57 |
| Edi-0 | 4 | 35467 | 3925 | 12434 | 17157 | 1951 | 13.56 |
| Oy-0 | 1 | 30405 | 2914 | 12033 | 13817 | 1641 | 12 |
| Oy-0 | 2 | 30579 | 2930 | 11893 | 14010 | 1746 | 12.8 |
| Oy-0 | 3 | 30608 | 2740 | 12191 | 13914 | 1763 | 12.63 |
| Bu-0 | 1 | 21103 | 2977 | 4449 | 13251 | 426 | 8.74 |
| Bu-0 | 2 | 21101 | 3150 | 4040 | 13539 | 372 | 8.43 |
| Bu-0 | 3 | 21054 | 2852 | 4200 | 13572 | 430 | 9.29 |
| Bu-0 | 4 | 29852 | 4197 | 5619 | 19486 | 550 | 8.92 |
| CIBC5 | 1 | 29489 | 3090 | 11048 | 13995 | 1356 | 10.93 |
| CIBC5 | 2 | 29850 | 2557 | 11675 | 14119 | 1499 | 11.38 |
| CIBC5 | 3 | 29702 | 2727 | 10758 | 14778 | 1439 | 11.8 |
| CIBC5 | 4 | 29709 | 2748 | 11362 | 14180 | 1419 | 11.1 |
| Mh-0 | 1 | 30063 | 2541 | 11952 | 14275 | 1295 | 9.78 |
| Mh-0 | 2 | 30051 | 2478 | 12209 | 13990 | 1374 | 10.12 |
| Mh-0 | 3 | 30006 | 2582 | 11576 | 14568 | 1280 | 9.96 |
| Mh-0 | 4 | 30054 | 2524 | 11896 | 14241 | 1393 | 10.48 |
| Nw-0 | 1 | 30136 | 3543 | 11576 | 13096 | 1921 | 14.23 |
| Nw-0 | 2 | 30015 | 3384 | 11141 | 13454 | 2036 | 15.45 |
| Nw-0 | 3 | 29947 | 3023 | 11560 | 13504 | 1860 | 13.86 |
| Nw-0 | 4 | 30057 | 3100 | 11657 | 13377 | 1923 | 14.16 |
| Wl-0 | 1 | 30094 | 2605 | 11622 | 14722 | 1145 | 8.97 |
| Wl-0 | 2 | 30038 | 2630 | 11387 | 14843 | 1178 | 9.38 |
| Wl-0 | 3 | 30060 | 2584 | 11710 | 14473 | 1293 | 9.94 |
| Wl-0 | 4 | 30009 | 2653 | 11433 | 14717 | 1206 | 9.54 |
| RRS7 | 1 | 30093 | 2705 | 10975 | 14857 | 1556 | 12.42 |
| RRS7 | 2 | 29975 | 2171 | 12289 | 14036 | 1479 | 10.74 |
| RRS7 | 3 | 30079 | 2183 | 11025 | 15278 | 1593 | 12.62 |
| RRS7 | 4 | 29962 | 2122 | 11379 | 15058 | 1403 | 10.98 |
| Zu-0 | 1 | 29918 | 2560 | 9742 | 16490 | 1126 | 10.36 |
| Zu-0 | 2 | 29834 | 2652 | 8634 | 17586 | 962 | 10.03 |
| Zu-0 | 3 | 29763 | 2725 | 8892 | 17227 | 919 | 9.37 |
| Kas | 1 | 20462 | 1561 | 8143 | 9469 | 1289 | 13.67 |
| Kas | 2 | 40581 | 3077 | 3967 | 32920 | 617 | 13.46 |
| Kas | 3 | 50469 | 3478 | 4862 | 41363 | 766 | 13.61 |
| Kas | 4 | 50350 | 3781 | 8513 | 36836 | 1220 | 12.53 |
| Sf-2 | 1 | 31325 | 4464 | 9642 | 15766 | 1453 | 13.1 |
| Sf-2 | 2 | 29774 | 3674 | 9671 | 15126 | 1303 | 11.87 |
| Sf-2 | 3 | 30689 | 3822 | 9891 | 15636 | 1340 | 11.93 |
