# Supplementary material for: Juxtaposition of heterozygous and homozygous regions causes reciprocal crossover remodelling via interference during Arabidopsis meiosis
Source: eLife. 2015 Mar 27;4:e03708. doi: 10.7554/eLife.03708 (PMC4407271; doi:10.7554/eLife.03708)
Supplement: Figure 4—source data 1. — DOI: http://dx.doi.org/10.7554/eLife.03708.018 [file elife03708s007.docx]

**Figure 4 – Source Data 1. *420* Col/Ct F_2_ fluorescent seed count data.** For the formula used for cM calculation please see Materials and Methods.

| Individual | Green alone | Red alone | Red and Green | Neither | Total seed | cM | Green:Non-green | Red:  Non-red | Green alone:Red alone |
| --- | --- | --- | --- | --- | --- | --- | --- | --- | --- |
| 1 | 106 | 113 | 1630 | 497 | 2346 | 9.82 | 2.85 | 2.89 | 0.94 |
| 2 | 158 | 148 | 2334 | 626 | 3266 | 9.85 | 3.22 | 3.17 | 1.07 |
| 3 | 80 | 86 | 1260 | 313 | 1739 | 10.05 | 3.36 | 3.42 | 0.93 |
| 4 | 100 | 88 | 1353 | 421 | 1962 | 10.09 | 2.85 | 2.77 | 1.14 |
| 5 | 117 | 92 | 1479 | 454 | 2142 | 10.29 | 2.92 | 2.75 | 1.27 |
| 6 | 114 | 98 | 1482 | 423 | 2117 | 10.57 | 3.06 | 2.94 | 1.16 |
| 7 | 110 | 99 | 1476 | 389 | 2074 | 10.64 | 3.25 | 3.16 | 1.11 |
| 8 | 130 | 117 | 1708 | 485 | 2440 | 10.69 | 3.05 | 2.97 | 1.11 |
| 9 | 111 | 110 | 1487 | 451 | 2159 | 10.82 | 2.85 | 2.84 | 1.01 |
| 10 | 129 | 143 | 1764 | 595 | 2631 | 10.94 | 2.57 | 2.63 | 0.90 |
| 11 | 46 | 44 | 608 | 172 | 870 | 10.94 | 3.03 | 2.99 | 1.05 |
| 12 | 180 | 158 | 2250 | 669 | 3257 | 10.98 | 2.94 | 2.84 | 1.14 |
| 13 | 120 | 91 | 1395 | 413 | 2019 | 11.06 | 3.01 | 2.79 | 1.32 |
| 14 | 122 | 131 | 1660 | 505 | 2418 | 11.08 | 2.80 | 2.86 | 0.93 |
| 15 | 142 | 164 | 1949 | 621 | 2876 | 11.28 | 2.66 | 2.77 | 0.87 |
| 16 | 129 | 143 | 1782 | 474 | 2528 | 11.41 | 3.10 | 3.19 | 0.90 |
| 17 | 110 | 90 | 1288 | 361 | 1849 | 11.48 | 3.10 | 2.93 | 1.22 |
| 18 | 153 | 133 | 1790 | 542 | 2618 | 11.60 | 2.88 | 2.77 | 1.15 |
| 19 | 117 | 106 | 1454 | 339 | 2016 | 11.75 | 3.53 | 3.42 | 1.10 |
| 20 | 123 | 132 | 1579 | 468 | 2302 | 11.77 | 2.84 | 2.90 | 0.93 |
| 21 | 141 | 152 | 1845 | 500 | 2638 | 11.80 | 3.05 | 3.12 | 0.93 |
| 22 | 57 | 67 | 749 | 213 | 1086 | 12.16 | 2.88 | 3.02 | 0.85 |
| 23 | 136 | 142 | 1705 | 447 | 2430 | 12.18 | 3.13 | 3.17 | 0.96 |
| 24 | 116 | 129 | 1492 | 393 | 2130 | 12.25 | 3.08 | 3.18 | 0.90 |
| 25 | 147 | 155 | 1824 | 479 | 2605 | 12.36 | 3.11 | 3.16 | 0.95 |
| 26 | 198 | 179 | 2291 | 552 | 3220 | 12.49 | 3.40 | 3.29 | 1.11 |
| 27 | 149 | 119 | 1565 | 451 | 2284 | 12.52 | 3.01 | 2.81 | 1.25 |
| 28 | 76 | 67 | 831 | 244 | 1218 | 12.52 | 2.92 | 2.81 | 1.13 |
| 29 | 122 | 139 | 1510 | 449 | 2220 | 12.54 | 2.78 | 2.89 | 0.88 |
| 30 | 128 | 116 | 1426 | 400 | 2070 | 12.58 | 3.01 | 2.92 | 1.10 |
| 31 | 108 | 112 | 1278 | 356 | 1854 | 12.67 | 2.96 | 3.00 | 0.96 |
| 32 | 96 | 88 | 1067 | 292 | 1543 | 12.74 | 3.06 | 2.98 | 1.09 |
| 33 | 129 | 86 | 1260 | 326 | 1801 | 12.75 | 3.37 | 2.96 | 1.50 |
| 34 | 209 | 188 | 2259 | 666 | 3322 | 12.77 | 2.89 | 2.80 | 1.11 |
| 35 | 103 | 118 | 1278 | 345 | 1844 | 12.80 | 2.98 | 3.12 | 0.87 |
| 36 | 133 | 109 | 1356 | 413 | 2011 | 12.86 | 2.85 | 2.68 | 1.22 |
| 37 | 161 | 150 | 1740 | 515 | 2566 | 12.96 | 2.86 | 2.80 | 1.07 |
| 38 | 147 | 152 | 1633 | 508 | 2440 | 13.11 | 2.70 | 2.73 | 0.97 |
| 39 | 144 | 134 | 1548 | 442 | 2268 | 13.12 | 2.94 | 2.87 | 1.07 |
| 40 | 135 | 118 | 1445 | 364 | 2062 | 13.13 | 3.28 | 3.13 | 1.14 |
| 41 | 93 | 74 | 928 | 262 | 1357 | 13.17 | 3.04 | 2.82 | 1.26 |
| 42 | 121 | 108 | 1273 | 347 | 1849 | 13.26 | 3.06 | 2.95 | 1.12 |
| 43 | 130 | 116 | 1358 | 375 | 1979 | 13.32 | 3.03 | 2.92 | 1.12 |
| 44 | 152 | 153 | 1659 | 487 | 2451 | 13.33 | 2.83 | 2.84 | 0.99 |
| 45 | 161 | 155 | 1728 | 483 | 2527 | 13.40 | 2.96 | 2.92 | 1.04 |
| 46 | 105 | 95 | 1082 | 316 | 1598 | 13.42 | 2.89 | 2.80 | 1.11 |
| 47 | 90 | 77 | 914 | 245 | 1326 | 13.51 | 3.12 | 2.96 | 1.17 |
| 48 | 169 | 156 | 1772 | 483 | 2580 | 13.51 | 3.04 | 2.96 | 1.08 |
| 49 | 153 | 139 | 1581 | 441 | 2314 | 13.53 | 2.99 | 2.90 | 1.10 |
| 50 | 111 | 105 | 1190 | 302 | 1708 | 13.57 | 3.20 | 3.14 | 1.06 |
| 51 | 121 | 112 | 1260 | 347 | 1840 | 13.59 | 3.01 | 2.93 | 1.08 |
| 52 | 65 | 120 | 1025 | 248 | 1458 | 13.62 | 2.96 | 3.66 | 0.54 |
| 53 | 132 | 122 | 1392 | 345 | 1991 | 13.70 | 3.26 | 3.17 | 1.08 |
| 54 | 88 | 74 | 845 | 255 | 1262 | 13.79 | 2.84 | 2.68 | 1.19 |
| 55 | 110 | 123 | 1225 | 355 | 1813 | 13.80 | 2.79 | 2.90 | 0.89 |
| 56 | 129 | 116 | 1325 | 336 | 1906 | 13.81 | 3.22 | 3.10 | 1.11 |
| 57 | 167 | 173 | 492 | 1811 | 2643 | 13.82 | 0.33 | 0.34 | 0.97 |
| 58 | 123 | 104 | 1194 | 338 | 1759 | 13.87 | 2.98 | 2.82 | 1.18 |
| 59 | 91 | 81 | 917 | 242 | 1331 | 13.89 | 3.12 | 3.00 | 1.12 |
| 60 | 132 | 139 | 1420 | 397 | 2088 | 13.95 | 2.90 | 2.95 | 0.95 |
| 61 | 153 | 117 | 1416 | 387 | 2073 | 14.01 | 3.11 | 2.84 | 1.31 |
| 62 | 107 | 104 | 1085 | 320 | 1616 | 14.04 | 2.81 | 2.78 | 1.03 |
| 63 | 113 | 91 | 1056 | 293 | 1553 | 14.13 | 3.04 | 2.83 | 1.24 |
| 64 | 130 | 151 | 1436 | 391 | 2108 | 14.36 | 2.89 | 3.05 | 0.86 |
| 65 | 144 | 131 | 1392 | 388 | 2055 | 14.42 | 2.96 | 2.86 | 1.10 |
| 66 | 124 | 110 | 1189 | 320 | 1743 | 14.47 | 3.05 | 2.93 | 1.13 |
| 67 | 140 | 138 | 1397 | 392 | 2067 | 14.50 | 2.90 | 2.89 | 1.01 |
| 68 | 138 | 128 | 1338 | 370 | 1974 | 14.53 | 2.96 | 2.89 | 1.08 |
| 69 | 93 | 101 | 964 | 259 | 1417 | 14.78 | 2.94 | 3.03 | 0.92 |
| 70 | 137 | 145 | 1391 | 381 | 2054 | 14.83 | 2.90 | 2.97 | 0.94 |
| 71 | 175 | 158 | 1705 | 382 | 2420 | 14.87 | 3.48 | 3.34 | 1.11 |
| 72 | 111 | 84 | 977 | 244 | 1416 | 14.88 | 3.32 | 2.99 | 1.32 |
| 73 | 111 | 114 | 1113 | 290 | 1628 | 14.94 | 3.03 | 3.06 | 0.97 |
| 74 | 135 | 130 | 1289 | 354 | 1908 | 15.02 | 2.94 | 2.90 | 1.04 |
| 75 | 145 | 151 | 1424 | 409 | 2129 | 15.03 | 2.80 | 2.84 | 0.96 |
| 76 | 136 | 151 | 1409 | 360 | 2056 | 15.10 | 3.02 | 3.15 | 0.90 |
| 77 | 146 | 138 | 1369 | 379 | 2032 | 15.12 | 2.93 | 2.87 | 1.06 |
| 78 | 189 | 162 | 1700 | 460 | 2511 | 15.12 | 3.04 | 2.87 | 1.17 |
| 79 | 130 | 112 | 1173 | 308 | 1723 | 15.20 | 3.10 | 2.93 | 1.16 |
| 80 | 99 | 95 | 960 | 225 | 1379 | 15.23 | 3.31 | 3.26 | 1.04 |
| 81 | 175 | 163 | 1618 | 436 | 2392 | 15.30 | 2.99 | 2.91 | 1.07 |
| 82 | 95 | 119 | 1023 | 277 | 1514 | 15.31 | 2.82 | 3.07 | 0.80 |
| 83 | 107 | 137 | 1147 | 329 | 1720 | 15.37 | 2.69 | 2.94 | 0.78 |
| 84 | 258 | 233 | 2330 | 638 | 3459 | 15.38 | 2.97 | 2.86 | 1.11 |
| 85 | 145 | 147 | 1362 | 403 | 2057 | 15.38 | 2.74 | 2.75 | 0.99 |
| 86 | 125 | 152 | 1298 | 375 | 1950 | 15.39 | 2.70 | 2.90 | 0.82 |
| 87 | 161 | 174 | 1584 | 422 | 2341 | 15.51 | 2.93 | 3.02 | 0.93 |
| 88 | 134 | 125 | 1208 | 335 | 1802 | 15.59 | 2.92 | 2.84 | 1.07 |
| 89 | 198 | 198 | 1867 | 486 | 2749 | 15.63 | 3.02 | 3.02 | 1.00 |
| 90 | 97 | 83 | 841 | 228 | 1249 | 15.63 | 3.02 | 2.84 | 1.17 |
| 91 | 122 | 118 | 1111 | 307 | 1658 | 15.71 | 2.90 | 2.86 | 1.03 |
| 92 | 74 | 74 | 684 | 187 | 1019 | 15.77 | 2.90 | 2.90 | 1.00 |
| 93 | 113 | 117 | 1080 | 273 | 1583 | 15.77 | 3.06 | 3.10 | 0.97 |
| 94 | 164 | 140 | 1397 | 379 | 2080 | 15.88 | 3.01 | 2.83 | 1.17 |
| 95 | 92 | 117 | 971 | 249 | 1429 | 15.89 | 2.90 | 3.19 | 0.79 |
| 96 | 118 | 136 | 1170 | 307 | 1731 | 15.94 | 2.91 | 3.07 | 0.87 |
| 97 | 82 | 102 | 858 | 209 | 1251 | 15.99 | 3.02 | 3.30 | 0.80 |
| 98 | 94 | 109 | 945 | 231 | 1379 | 16.00 | 3.06 | 3.24 | 0.86 |
| 99 | 106 | 114 | 998 | 272 | 1490 | 16.05 | 2.86 | 2.94 | 0.93 |
| 100 | 183 | 170 | 1610 | 423 | 2386 | 16.09 | 3.02 | 2.94 | 1.08 |
| 101 | 140 | 149 | 1305 | 352 | 1946 | 16.16 | 2.88 | 2.96 | 0.94 |
| 102 | 181 | 193 | 1730 | 409 | 2513 | 16.19 | 3.17 | 3.26 | 0.94 |
| 103 | 60 | 57 | 525 | 141 | 783 | 16.27 | 2.95 | 2.90 | 1.05 |
| 104 | 66 | 51 | 526 | 140 | 783 | 16.27 | 3.10 | 2.80 | 1.29 |
| 105 | 291 | 260 | 2480 | 650 | 3681 | 16.30 | 3.05 | 2.91 | 1.12 |
| 106 | 86 | 87 | 779 | 200 | 1152 | 16.35 | 3.01 | 3.03 | 0.99 |
| 107 | 164 | 178 | 1391 | 536 | 2269 | 16.42 | 2.18 | 2.24 | 0.92 |
| 108 | 156 | 130 | 1270 | 331 | 1887 | 16.52 | 3.09 | 2.87 | 1.20 |
| 109 | 159 | 145 | 1332 | 364 | 2000 | 16.57 | 2.93 | 2.82 | 1.10 |
| 110 | 160 | 166 | 1414 | 390 | 2130 | 16.70 | 2.83 | 2.87 | 0.96 |
| 111 | 193 | 146 | 1426 | 422 | 2187 | 16.93 | 2.85 | 2.56 | 1.32 |
| 112 | 133 | 147 | 1196 | 329 | 1805 | 16.95 | 2.79 | 2.91 | 0.90 |
| 113 | 181 | 213 | 1646 | 476 | 2516 | 17.13 | 2.65 | 2.83 | 0.85 |
| 114 | 107 | 104 | 957 | 172 | 1340 | 17.23 | 3.86 | 3.80 | 1.03 |
| 115 | 179 | 162 | 1446 | 359 | 2146 | 17.40 | 3.12 | 2.99 | 1.10 |
| 116 | 102 | 51 | 652 | 147 | 952 | 17.62 | 3.81 | 2.82 | 2.00 |
| 117 | 223 | 192 | 1730 | 434 | 2579 | 17.65 | 3.12 | 2.93 | 1.16 |
| 118 | 171 | 168 | 1406 | 341 | 2086 | 17.84 | 3.10 | 3.07 | 1.02 |
| 119 | 189 | 201 | 1606 | 380 | 2376 | 18.04 | 3.09 | 3.18 | 0.94 |
| 120 | 214 | 194 | 1666 | 406 | 2480 | 18.09 | 3.13 | 3.00 | 1.10 |
| 121 | 122 | 141 | 1052 | 269 | 1584 | 18.27 | 2.86 | 3.05 | 0.87 |
| 122 | 134 | 133 | 1046 | 258 | 1571 | 18.75 | 3.02 | 3.01 | 1.01 |
| 123 | 231 | 225 | 1767 | 440 | 2663 | 18.91 | 3.00 | 2.97 | 1.03 |
| 124 | 83 | 73 | 605 | 150 | 911 | 18.91 | 3.09 | 2.91 | 1.14 |
| 125 | 89 | 92 | 700 | 170 | 1051 | 19.03 | 3.01 | 3.06 | 0.97 |
| 126 | 317 | 274 | 2212 | 569 | 3372 | 19.41 | 3.00 | 2.81 | 1.16 |
| 127 | 227 | 189 | 1538 | 387 | 2341 | 19.71 | 3.06 | 2.81 | 1.20 |
| 128 | 211 | 202 | 1511 | 380 | 2304 | 19.91 | 2.96 | 2.90 | 1.04 |
| 129 | 271 | 257 | 1938 | 451 | 2917 | 20.13 | 3.12 | 3.04 | 1.05 |
| 130 | 217 | 195 | 1484 | 362 | 2258 | 20.31 | 3.05 | 2.90 | 1.11 |
| 131 | 72 | 91 | 580 | 148 | 891 | 20.37 | 2.73 | 3.05 | 0.79 |
| 132 | 163 | 165 | 1183 | 274 | 1785 | 20.47 | 3.07 | 3.08 | 0.99 |
| 133 | 198 | 220 | 1426 | 366 | 2210 | 21.15 | 2.77 | 2.92 | 0.90 |
| 134 | 181 | 196 | 1297 | 302 | 1976 | 21.36 | 2.97 | 3.09 | 0.92 |
| 135 | 117 | 93 | 723 | 162 | 1095 | 21.49 | 3.29 | 2.92 | 1.26 |
| 136 | 95 | 98 | 638 | 153 | 984 | 22.04 | 2.92 | 2.97 | 0.97 |
| 137 | 100 | 96 | 609 | 144 | 949 | 23.39 | 2.95 | 2.89 | 1.04 |
| 138 | 138 | 128 | 794 | 191 | 1251 | 24.19 | 2.92 | 2.80 | 1.08 |
| 139 | 210 | 181 | 1167 | 279 | 1837 | 24.22 | 2.99 | 2.76 | 1.16 |
