# Supplementary material for: Juxtaposition of heterozygous and homozygous regions causes reciprocal crossover remodelling via interference during Arabidopsis meiosis
Source: eLife. 2015 Mar 27;4:e03708. doi: 10.7554/eLife.03708 (PMC4407271; doi:10.7554/eLife.03708)
Supplement: Figure 4—source data 2. — DOI: http://dx.doi.org/10.7554/eLife.03708.019 [file elife03708s008.docx]

**Figure 4 – Source Data 2. *I2f* Col/Ct F_2_ fluorescent seed count data.** For the formula used for cM calculation please see Materials and Methods. Where samples had replicate measurements their mean was taken for further analysis.

| Replicate | Line | Total | Red | Red+Green | Neither | Green | Both+Yellow | RF ind |
| --- | --- | --- | --- | --- | --- | --- | --- | --- |
| 1 | 31-2 | 11,529 | 966 | 4,137 | 6,070 | 356 | 4,493 | 7.92 |
| 1 | 33-1 | 28,291 | 3,173 | 8,345 | 16,301 | 472 | 8,817 | 5.35 |
| 1 | 34-2 | 20,760 | 1,636 | 8,006 | 10,508 | 610 | 8,616 | 7.08 |
| 1 | 34-3 | 5,223 | 712 | 1,173 | 3,261 | 77 | 1,250 | 6.16 |
| 2 | 34-3 | 14,909 | 1,361 | 5,028 | 8,211 | 309 | 5,337 | 5.79 |
| 1 | 34-4 | 11,403 | 831 | 4,179 | 6,161 | 232 | 4,411 | 5.26 |
| 1 | 35-4 | 15,703 | 1,766 | 4,765 | 8,694 | 478 | 5,243 | 9.12 |
| 1 | 37-2 | 20,851 | 2,333 | 5,976 | 12,200 | 342 | 6,318 | 5.41 |
| 1 | 38-6 | 28,817 | 2,510 | 10,443 | 14,843 | 1,021 | 11,464 | 8.91 |
| 1 | 39-1 | 25,064 | 2,347 | 8,482 | 13,646 | 589 | 9,071 | 6.49 |
| 1 | 40-1 | 6,007 | 516 | 1,935 | 3,395 | 161 | 2,096 | 7.68 |
| 2 | 40-1 | 16,803 | 1,300 | 5,546 | 9,533 | 424 | 5,970 | 7.10 |
| 1 | 41-1 | 9,634 | 1,918 | 1,390 | 6,214 | 112 | 1,502 | 7.46 |
| 2 | 41-1 | 18,773 | 1,628 | 5,982 | 10,608 | 555 | 6,537 | 8.49 |
| 1 | 41-2 | 10,615 | 1,353 | 2,782 | 6,249 | 231 | 3,013 | 7.67 |
| 1 | 41-5 | 9,815 | 1,292 | 2,238 | 6,142 | 143 | 2,381 | 6.01 |
| 2 | 41-5 | 14,121 | 990 | 5,124 | 7,610 | 397 | 5,521 | 7.19 |
| 1 | 44-5 | 21,195 | 2,827 | 4,991 | 12,809 | 568 | 5,559 | 10.22 |
| 1 | 45-1 | 5,457 | 523 | 1,885 | 2,935 | 114 | 1,999 | 5.70 |
| 2 | 45-1 | 10,508 | 588 | 4,023 | 5,615 | 282 | 4,305 | 6.55 |
| 1 | 45-2 | 5,250 | 703 | 1,092 | 3,384 | 71 | 1,163 | 6.10 |
| 2 | 45-2 | 13,787 | 1,289 | 3,477 | 8,775 | 246 | 3,723 | 6.61 |
| 1 | 45-4 | 19,547 | 1,738 | 6,103 | 11,293 | 413 | 6,516 | 6.34 |
| 1 | 46-2 | 22,076 | 1,943 | 7,166 | 12,391 | 576 | 7,742 | 7.44 |
| 1 | 48-2 | 23,655 | 1,842 | 8,144 | 13,161 | 508 | 8,652 | 5.87 |
| 1 | 48-4 | 20,532 | 1,451 | 7,828 | 10,786 | 467 | 8,295 | 5.63 |
| 1 | 15-1 | 9,243 | 1,085 | 2,769 | 5,222 | 167 | 2,936 | 5.69 |
| 2 | 15-1 | 16,851 | 1,273 | 6,058 | 9,138 | 382 | 6,440 | 5.93 |
| 1 | 16-1 | 6,756 | 555 | 2,511 | 3,540 | 150 | 2,661 | 5.64 |
| 2 | 16-1 | 13,179 | 750 | 5,506 | 6,553 | 370 | 5,876 | 6.30 |
| 1 | 20-1 | 11,606 | 1,843 | 2,021 | 7,598 | 144 | 2,165 | 6.65 |
| 2 | 20-1 | 16,383 | 1,249 | 6,238 | 8,574 | 322 | 6,560 | 4.91 |
| 1 | 22-1 | 17,522 | 1,674 | 6,138 | 9,296 | 414 | 6,552 | 6.32 |
| 1 | 24-1 | 18,032 | 1,939 | 6,045 | 9,610 | 438 | 6,483 | 6.76 |
| 1 | 28-1 | 10,617 | 1,069 | 3,163 | 6,120 | 265 | 3,428 | 7.73 |
| 1 | 31-1 | 20,111 | 1,625 | 7,638 | 10,410 | 438 | 8,076 | 5.42 |
| 1 | 2-5 | 5,320 | 519 | 1,508 | 3,170 | 123 | 1,631 | 7.54 |
| 2 | 2-5 | 19,535 | 1,779 | 6,478 | 10,796 | 482 | 6,960 | 6.93 |
| 1 | 2-6 | 7,246 | 657 | 2,362 | 3,989 | 238 | 2,600 | 9.15 |
| 2 | 2-6 | 11,923 | 1,109 | 4,346 | 6,041 | 427 | 4,773 | 8.95 |
| 1 | 15-2 | 8,249 | 863 | 2,544 | 4,607 | 235 | 2,779 | 8.46 |
| 2 | 15-2 | 23,864 | 2,050 | 8,399 | 12,725 | 690 | 9,089 | 7.59 |
| 1 | 18-2 | 25,363 | 2,436 | 9,546 | 12,592 | 789 | 10,335 | 7.63 |
| 1 | 19-2 | 27,925 | 3,240 | 9,157 | 14,908 | 620 | 9,777 | 6.34 |
| 1 | 13-3 | 11,851 | 1,000 | 3,995 | 6,590 | 266 | 4,261 | 6.24 |
| 1 | 25-3 | 17,107 | 1,712 | 6,099 | 8,886 | 410 | 6,509 | 6.30 |
| 1 | 26-3 | 16,772 | 1,497 | 6,053 | 8,767 | 455 | 6,508 | 6.99 |
| 1 | 30-3 | 22,107 | 1,661 | 8,099 | 11,795 | 552 | 8,651 | 6.38 |
| 1 | 4-4 | 7,126 | 499 | 2,775 | 3,676 | 176 | 2,951 | 5.96 |
| 2 | 4-4 | 11,170 | 695 | 4,238 | 5,951 | 286 | 4,524 | 6.32 |
| 1 | 4-6 | 6,628 | 638 | 2,157 | 3,651 | 182 | 2,339 | 7.78 |
| 2 | 4-6 | 19,217 | 1,565 | 6,877 | 10,146 | 629 | 7,506 | 8.38 |
| 1 | 19-4 | 19,552 | 2,406 | 5,977 | 10,765 | 404 | 6,381 | 6.33 |
| 1 | 20-4 | 9,018 | 1,898 | 1,263 | 5,769 | 88 | 1,351 | 6.51 |
| 1 | 21-4 | 24,767 | 2,648 | 7,599 | 14,039 | 481 | 8,080 | 5.95 |
| 1 | 25-4 | 18,701 | 1,822 | 6,561 | 9,861 | 457 | 7,018 | 6.51 |
| 1 | 28-4 | 18,472 | 1,882 | 6,217 | 9,831 | 542 | 6,759 | 8.02 |
| 1 | 17-5 | 9,338 | 1,254 | 2,512 | 5,410 | 162 | 2,674 | 6.06 |
| 2 | 17-5 | 20,750 | 2,030 | 7,101 | 11,201 | 418 | 7,519 | 5.56 |
| 1 | 20-5 | 11,803 | 1,658 | 2,566 | 7,332 | 247 | 2,813 | 8.78 |
| 2 | 20-5 | 19,834 | 1,761 | 7,143 | 10,240 | 690 | 7,833 | 8.81 |
| 1 | 24-5 | 22,393 | 2,095 | 6,940 | 12,697 | 661 | 7,601 | 8.70 |
| 1 | 25-5 | 16,955 | 1,699 | 5,955 | 8,905 | 396 | 6,351 | 6.24 |
| 1 | 31-5 | 23,528 | 2,112 | 7,991 | 12,907 | 518 | 8,509 | 6.09 |
| 1 | 6-4 | 12,787 | 1,136 | 3,959 | 7,323 | 369 | 4,328 | 8.53 |
| 1 | 6-5 | 6,535 | 594 | 1,973 | 3,807 | 161 | 2,134 | 7.54 |
| 2 | 6-5 | 15,948 | 1,681 | 5,557 | 8,309 | 401 | 5,958 | 6.73 |
| 1 | 6-6 | 639 | 35 | 382 | 215 | 7 | 389 | 1.80 |
| 1 | 24-6 | 21,354 | 1,967 | 7,433 | 11,493 | 461 | 7,894 | 5.84 |
| 1 | 26-6 | 18,007 | 1,706 | 6,058 | 9,857 | 386 | 6,444 | 5.99 |
| 1 | 27-6 | 23,329 | 1,961 | 9,018 | 11,717 | 633 | 9,651 | 6.56 |
| 1 | 30-6 | 17,419 | 1,435 | 6,545 | 9,089 | 350 | 6,895 | 5.08 |
| 1 | 7-2 | 9,416 | 1,012 | 2,651 | 5,565 | 188 | 2,839 | 6.62 |
| 2 | 7-2 | 21,684 | 1,864 | 7,110 | 12,209 | 501 | 7,611 | 6.58 |
| 1 | 8-1 | 19,212 | 2,309 | 6,421 | 9,747 | 735 | 7,156 | 10.27 |
| 1 | 8-6 | 7,447 | 852 | 5,475 | 1,112 | 8 | 5,483 | 0.15 |
| 1 | 9-6 | 9,855 | 902 | 3,411 | 5,322 | 220 | 3,631 | 6.06 |
| 1 | 11-1 | 10,133 | 870 | 3,400 | 5,648 | 215 | 3,615 | 5.95 |
| 1 | 11-2 | 6,198 | 443 | 2,110 | 3,517 | 128 | 2,238 | 5.72 |
| 2 | 11-2 | 10,074 | 1,124 | 3,277 | 5,472 | 201 | 3,478 | 5.78 |
| 1 | 11-5 | 7,485 | 730 | 2,532 | 4,001 | 222 | 2,754 | 8.06 |
| 2 | 11-5 | 19,381 | 1,522 | 7,066 | 10,278 | 515 | 7,581 | 6.79 |
| 1 | 12-1 | 8,869 | 960 | 2,625 | 5,123 | 161 | 2,786 | 5.78 |
| 2 | 12-1 | 22,582 | 1,945 | 6,519 | 13,701 | 417 | 6,936 | 6.01 |
| 1 | 10b-2 | 6,131 | 701 | 1,622 | 3,710 | 98 | 1,720 | 5.70 |
| 2 | 10b-2 | 27,141 | 3,853 | 6,382 | 16,506 | 400 | 6,782 | 5.90 |
| 1 | 12b-2 | 9,480 | 851 | 3,053 | 5,369 | 207 | 3,260 | 6.35 |
| 2 | 12b-2 | 42,324 | 3,791 | 13,553 | 24,095 | 885 | 14,438 | 6.13 |
| 1 | 13b-5 | 18,784 | 1,849 | 6,177 | 10,296 | 462 | 6,639 | 6.96 |
| 2 | 13b-5 | 27,408 | 2,510 | 10,210 | 13,940 | 748 | 10,958 | 6.83 |
| 1 | 14b-3 | 14,729 | 1,265 | 5,210 | 7,811 | 443 | 5,653 | 7.84 |
| 2 | 14b-3 | 27,816 | 3,268 | 8,825 | 15,119 | 604 | 9,429 | 6.41 |
| 1 | 15b-5 | 3,309 | 276 | 808 | 2,159 | 66 | 874 | 7.55 |
| 2 | 15b-5 | 34,294 | 3,208 | 11,834 | 18,398 | 854 | 12,688 | 6.73 |
| 1 | 16b-1 | 29,529 | 3,123 | 9,369 | 16,328 | 709 | 10,078 | 7.04 |
| 1 | 16b-4 | 8,044 | 825 | 1,434 | 5,693 | 92 | 1,526 | 6.03 |
| 2 | 16b-4 | 23,940 | 3,495 | 5,430 | 14,731 | 284 | 5,714 | 4.97 |
| 1 | 1b-4 | 8,697 | 795 | 2,660 | 5,049 | 193 | 2,853 | 6.76 |
| 2 | 1b-4 | 13,517 | 1,141 | 5,099 | 6,937 | 340 | 5,439 | 6.25 |
| 1 | 2b-4 | 9,620 | 968 | 3,289 | 5,140 | 223 | 3,512 | 6.35 |
| 2 | 2b-4 | 22,964 | 2,450 | 7,777 | 12,160 | 577 | 8,354 | 6.91 |
| 1 | 2b-5 | 7,889 | 765 | 2,351 | 4,615 | 158 | 2,509 | 6.30 |
| 2 | 2b-5 | 14,130 | 1,214 | 5,406 | 7,183 | 327 | 5,733 | 5.70 |
| 1 | 3b-2 | 4,587 | 448 | 1,246 | 2,832 | 61 | 1,307 | 4.67 |
| 2 | 3b-2 | 16,116 | 2,738 | 3,896 | 9,288 | 194 | 4,090 | 4.74 |
| 1 | 3b-5 | 8,400 | 1,052 | 2,141 | 4,958 | 249 | 2,390 | 10.42 |
| 2 | 3b-5 | 23,769 | 3,259 | 6,693 | 13,174 | 643 | 7,336 | 8.76 |
| 1 | 4b-1 | 7,021 | 912 | 1,806 | 4,150 | 153 | 1,959 | 7.81 |
| 2 | 4b-1 | 20,509 | 2,524 | 6,127 | 11,440 | 418 | 6,545 | 6.39 |
| 1 | 4b-2 | 7,276 | 873 | 1,991 | 4,272 | 140 | 2,131 | 6.57 |
| 2 | 4b-2 | 22,799 | 2,166 | 8,351 | 11,698 | 584 | 8,935 | 6.54 |
| 1 | 4b-4 | 9,084 | 996 | 2,673 | 5,227 | 188 | 2,861 | 6.57 |
| 2 | 4b-4 | 28,009 | 2,668 | 10,233 | 14,382 | 726 | 10,959 | 6.62 |
| 1 | 7b-4 | 5,117 | 500 | 1,720 | 2,793 | 104 | 1,824 | 5.70 |
| 2 | 7b-4 | 25,047 | 2,574 | 9,063 | 12,872 | 538 | 9,601 | 5.60 |
| 1 | 8b-2 | 2,084 | 181 | 563 | 1,294 | 46 | 609 | 7.55 |
| 2 | 8b-2 | 43,916 | 5,425 | 10,957 | 26,785 | 749 | 11,706 | 6.40 |
