# Supplementary material for: Juxtaposition of heterozygous and homozygous regions causes reciprocal crossover remodelling via interference during Arabidopsis meiosis
Source: eLife. 2015 Mar 27;4:e03708. doi: 10.7554/eLife.03708 (PMC4407271; doi:10.7554/eLife.03708)
Supplement: Figure 4—source data 3. — DOI: http://dx.doi.org/10.7554/eLife.03708.020 [file elife03708s009.docx]

**Figure 4 – Source Data 3. *CEN3* Col/Ct F_2_ flow cytometry count data.** cM were calculated as 100 x R5/(R3+R5).

| Line | Total pollen | Red alone (Gate R2) | Red and Green (Gate R3) | Neither (Gate R4) | Green alone (Gate R5) | cM |
| --- | --- | --- | --- | --- | --- | --- |
| 68-5 | 21201 | 3191 | 8184 | 9330 | 496 | 5.71 |
| 57-4 | 42614 | 5083 | 11450 | 25224 | 857 | 6.96 |
| 11-2 | 31113 | 3293 | 5175 | 22253 | 392 | 7.04 |
| 73-3 | 12547 | 2477 | 4114 | 5640 | 316 | 7.13 |
| 35-3 | 31974 | 4345 | 9296 | 17613 | 720 | 7.19 |
| 74-5 | 10648 | 1173 | 4424 | 4706 | 345 | 7.23 |
| 37-2 | 44359 | 3071 | 7562 | 33131 | 595 | 7.29 |
| 63-2 | 34045 | 4396 | 13219 | 15371 | 1059 | 7.42 |
| 22-3 | 39698 | 5405 | 12093 | 21220 | 980 | 7.50 |
| 102-2 | 16086 | 1707 | 4152 | 9876 | 351 | 7.79 |
| 94-1 | 26686 | 3495 | 6872 | 15724 | 595 | 7.97 |
| 29-3 | 31826 | 4021 | 9377 | 17612 | 816 | 8.01 |
| 45-2 | 42196 | 5734 | 9924 | 25646 | 892 | 8.25 |
| 70-3 | 32007 | 5177 | 10803 | 15042 | 985 | 8.36 |
| 62-2 | 51337 | 5845 | 17927 | 25911 | 1654 | 8.45 |
| 96-4 | 20416 | 2660 | 6111 | 11076 | 569 | 8.52 |
| 70-5 | 11648 | 1604 | 4753 | 4840 | 451 | 8.67 |
| 74-1 | 32112 | 4912 | 10693 | 15491 | 1016 | 8.68 |
| 60-3 | 30289 | 3780 | 11318 | 14113 | 1078 | 8.70 |
| 27-4 | 25235 | 1847 | 3407 | 19656 | 325 | 8.71 |
| 44-1 | 43430 | 6116 | 9972 | 26390 | 952 | 8.71 |
| 29-1 | 35628 | 4549 | 8838 | 21376 | 865 | 8.91 |
| 94-4 | 30266 | 3665 | 8100 | 17699 | 802 | 9.01 |
| 78-1 | 51335 | 5713 | 16098 | 27903 | 1621 | 9.15 |
| 101-1 | 20345 | 2598 | 6083 | 11045 | 619 | 9.24 |
| 77-3 | 23898 | 3604 | 9377 | 9949 | 968 | 9.36 |
| 101-2 | 15154 | 1832 | 4985 | 7818 | 519 | 9.43 |
| 86-2 | 33326 | 4214 | 8618 | 19596 | 898 | 9.44 |
| 102-4 | 40205 | 5313 | 11335 | 22374 | 1183 | 9.45 |
| 88-5 | 36017 | 4692 | 10601 | 19615 | 1109 | 9.47 |
| 58-3 | 25434 | 3409 | 9532 | 11485 | 1008 | 9.56 |
| 80-2 | 37070 | 5176 | 10352 | 20429 | 1113 | 9.71 |
| 25-1 | 29650 | 3614 | 9120 | 15922 | 994 | 9.83 |
| 85-3 | 22474 | 3477 | 6246 | 12065 | 686 | 9.90 |
| 67-4 | 15119 | 1804 | 4943 | 7829 | 543 | 9.90 |
| 69-4 | 11656 | 2247 | 3356 | 5684 | 369 | 9.91 |
| 107-1 | 31351 | 4252 | 8498 | 17663 | 938 | 9.94 |
| 89-1 | 18111 | 2321 | 5465 | 9720 | 605 | 9.97 |
| 108-3 | 37056 | 5465 | 10951 | 19427 | 1213 | 9.97 |
| 21-2 | 39699 | 5759 | 10999 | 21716 | 1225 | 10.02 |
| 49-1 | 42433 | 5713 | 10111 | 25476 | 1133 | 10.08 |
| 23-2 | 60653 | 7408 | 5719 | 46879 | 647 | 10.16 |
| 64-1 | 26864 | 5165 | 7703 | 13124 | 872 | 10.17 |
| 93-2 | 40290 | 4922 | 11233 | 22842 | 1293 | 10.32 |
| 99-1 | 30302 | 3233 | 9384 | 16604 | 1081 | 10.33 |
| 77-1 | 50945 | 7105 | 14000 | 28221 | 1619 | 10.37 |
| 91-3 | 20630 | 2953 | 6190 | 10771 | 716 | 10.37 |
| 46-1 | 15132 | 1900 | 6024 | 6511 | 697 | 10.37 |
| 76-4 | 34745 | 3820 | 10744 | 18930 | 1251 | 10.43 |
| 28-3 | 36869 | 4817 | 4293 | 27256 | 503 | 10.49 |
| 98-5 | 25315 | 3166 | 6557 | 14820 | 772 | 10.53 |
| 90-4 | 31812 | 5378 | 6140 | 19570 | 724 | 10.55 |
| 62-4 | 50909 | 5994 | 15974 | 27056 | 1885 | 10.55 |
| 18-4 | 35939 | 5700 | 9212 | 19938 | 1089 | 10.57 |
| 46-4 | 31377 | 5394 | 5039 | 20348 | 596 | 10.58 |
| 71-4 | 46123 | 6356 | 13701 | 24438 | 1628 | 10.62 |
| 69-2 | 17385 | 3013 | 5259 | 8488 | 625 | 10.62 |
| 86-1 | 26044 | 5398 | 5066 | 14976 | 604 | 10.65 |
| 83-1 | 49161 | 8268 | 9658 | 30080 | 1155 | 10.68 |
| 26-1 | 30193 | 4803 | 7133 | 17388 | 869 | 10.86 |
| 21-3 | 39806 | 6082 | 10464 | 21983 | 1277 | 10.88 |
| 105-2 | 20242 | 2495 | 6341 | 10632 | 774 | 10.88 |
| 8-3 | 45392 | 6522 | 7044 | 30959 | 867 | 10.96 |
| 9-3 | 39577 | 4985 | 5514 | 28394 | 684 | 11.04 |
| 7-1 | 85964 | 11284 | 8711 | 64870 | 1099 | 11.20 |
| 53-3 | 26917 | 3774 | 6762 | 15525 | 856 | 11.24 |
| 81-3 | 46358 | 5206 | 15905 | 23227 | 2020 | 11.27 |
| 104-4 | 31914 | 3079 | 10156 | 17388 | 1291 | 11.28 |
| 58-2 | 33234 | 3540 | 10622 | 17721 | 1351 | 11.28 |
| 91-1 | 16648 | 2403 | 5065 | 8535 | 645 | 11.30 |
| 104-1 | 50252 | 7518 | 13719 | 27261 | 1754 | 11.34 |
| 3 | 101724 | 13993 | 5246 | 81810 | 675 | 11.40 |
| 86-4 | 35874 | 5314 | 6873 | 22802 | 885 | 11.41 |
| 19-4 | 39931 | 6531 | 9748 | 22394 | 1258 | 11.43 |
| 13-1 | 39844 | 5296 | 3917 | 30125 | 506 | 11.44 |
| 92-3 | 22066 | 3572 | 4231 | 13707 | 556 | 11.61 |
| 100-3 | 35453 | 4420 | 10542 | 19091 | 1400 | 11.72 |
| 8-4 | 30048 | 3253 | 3391 | 22953 | 451 | 11.74 |
| 96-3 | 10468 | 1809 | 2409 | 5927 | 323 | 11.82 |
| 14-4 | 29924 | 3258 | 4548 | 21507 | 611 | 11.84 |
| 27-2 | 43718 | 5638 | 4941 | 32473 | 666 | 11.88 |
| 75-5 | 12998 | 2153 | 4300 | 5964 | 581 | 11.90 |
| 78-4 | 15247 | 2708 | 3971 | 8029 | 539 | 11.95 |
| 51-3 | 32926 | 4960 | 6545 | 20528 | 893 | 12.01 |
| 65-5 | 18864 | 2976 | 4394 | 10893 | 601 | 12.03 |
| 106-4 | 35979 | 6101 | 7325 | 21546 | 1007 | 12.09 |
| 13-3 | 29164 | 2841 | 5079 | 20540 | 704 | 12.17 |
| 66-4 | 16251 | 2803 | 3853 | 9055 | 540 | 12.29 |
| 66-5 | 35949 | 5198 | 9144 | 20309 | 1298 | 12.43 |
| 89-5 | 26827 | 4696 | 4236 | 17292 | 603 | 12.46 |
| 39-1 | 48905 | 3406 | 8623 | 35641 | 1235 | 12.53 |
| 99-4 | 41036 | 5771 | 9250 | 24683 | 1332 | 12.59 |
| 83-3 | 50855 | 6184 | 15394 | 27048 | 2229 | 12.65 |
| 105-3 | 13379 | 2135 | 3655 | 7056 | 533 | 12.73 |
| 38-2 | 22504 | 4012 | 7060 | 10400 | 1032 | 12.75 |
| 101-3 | 31156 | 4442 | 9022 | 16372 | 1320 | 12.76 |
| 60-1 | 32487 | 4483 | 7024 | 19940 | 1040 | 12.90 |
| 88-3 | 40654 | 5744 | 10728 | 22583 | 1599 | 12.97 |
| 14-2 | 32556 | 6634 | 3675 | 21694 | 553 | 13.08 |
| 66-2 | 10589 | 1850 | 2733 | 5594 | 412 | 13.10 |
| 50-1 | 34109 | 5274 | 5413 | 22600 | 822 | 13.18 |
| 36-3 | 27551 | 5877 | 2394 | 18910 | 370 | 13.39 |
| 95-4 | 30539 | 5188 | 5735 | 18727 | 889 | 13.42 |
| 104-3 | 30458 | 4788 | 7442 | 17069 | 1159 | 13.48 |
| 65-4 | 40646 | 7216 | 8226 | 23915 | 1289 | 13.55 |
| 87-2 | 21166 | 3214 | 4350 | 12919 | 683 | 13.57 |
| 43-2 | 37179 | 5155 | 10093 | 20314 | 1617 | 13.81 |
| 82-3 | 50868 | 7535 | 14495 | 26500 | 2338 | 13.89 |
| 52-3 | 44679 | 7889 | 4895 | 31103 | 792 | 13.93 |
| 92-4 | 16707 | 3212 | 2488 | 10602 | 405 | 14.00 |
| 66-1 | 15266 | 2396 | 1960 | 10590 | 320 | 14.04 |
| 84-3 | 13702 | 2166 | 3494 | 7465 | 577 | 14.17 |
| 21-5 | 50548 | 3571 | 2340 | 44246 | 391 | 14.32 |
| 100-2 | 20339 | 3641 | 4008 | 12019 | 671 | 14.34 |
| 78-2 | 12701 | 2828 | 2362 | 7113 | 398 | 14.42 |
| 103-1 | 41690 | 7223 | 6686 | 26631 | 1150 | 14.68 |
| 86-3 | 20976 | 3700 | 4948 | 11448 | 880 | 15.10 |
| 55-1 | 26534 | 3785 | 6098 | 15558 | 1093 | 15.20 |
| 89-3 | 37066 | 4517 | 9594 | 21134 | 1821 | 15.95 |
| 38-1 | 84452 | 11458 | 8697 | 62566 | 1731 | 16.60 |
| 5 | 109248 | 8362 | 1307 | 99315 | 264 | 16.80 |
