# Supplementary material for: Juxtaposition of heterozygous and homozygous regions causes reciprocal crossover remodelling via interference during Arabidopsis meiosis
Source: eLife. 2015 Mar 27;4:e03708. doi: 10.7554/eLife.03708 (PMC4407271; doi:10.7554/eLife.03708)
Supplement: Figure 5—source data 1. — DOI: http://dx.doi.org/10.7554/eLife.03708.025 [file elife03708s010.docx]

**Figure 5 – Source Data 1. Three colour *I3bc* FTL flow cytometry count data.** For the formula used for cM calculation please see Materials and Methods.

| *I3bc* genotype | Replicate | BYR | byr | bYr | ByR | BYr | byR | bYR | Byr | Total |
| --- | --- | --- | --- | --- | --- | --- | --- | --- | --- | --- |
| HOM-HOM | 1 | 10,520 | 10,696 | 67 | 45 | 671 | 661 | 2,510 | 2,403 | 27,573 |
| HOM-HOM | 2 | 26,204 | 27,147 | 128 | 101 | 1,626 | 1,643 | 6,198 | 6,106 | 69,153 |
| HOM-HOM | 3 | 23,042 | 23,433 | 128 | 96 | 1,504 | 1,452 | 5,624 | 5,462 | 60,741 |
| HOM-HOM | 4 | 46,914 | 49,005 | 216 | 223 | 3,198 | 3,208 | 11,611 | 11,418 | 125,793 |
| HOM-HOM | 5 | 10,808 | 11,088 | 56 | 43 | 653 | 646 | 2,176 | 2,171 | 27,641 |
| HOM-HOM | 6 | 12,592 | 13,689 | 52 | 54 | 794 | 824 | 2,642 | 2,617 | 33,264 |
| HOM-HOM | 7 | 30,537 | 33,136 | 151 | 130 | 1,840 | 1,899 | 6,343 | 6,376 | 80,412 |
| Total | Total | 160,617 | 168,194 | 798 | 692 | 10,286 | 10,333 | 37,104 | 36,553 | 424,577 |
| HET-HOM | 1 | 23,996 | 23,778 | 114 | 98 | 1,141 | 1,158 | 7,326 | 6,967 | 64,578 |
| HET-HOM | 2 | 15,623 | 16,143 | 52 | 62 | 686 | 655 | 4,572 | 4,454 | 42,247 |
| HET-HOM | 3 | 21,850 | 21,633 | 116 | 82 | 1,043 | 1,038 | 6,785 | 6,650 | 59,197 |
| HET-HOM | 4 | 30,685 | 31,209 | 134 | 124 | 1,525 | 1,484 | 9,244 | 9,031 | 83,436 |
| HET-HOM | 5 | 27,606 | 28,152 | 123 | 120 | 1,305 | 1,260 | 7,877 | 7,763 | 74,206 |
| HET-HOM | 6 | 24,493 | 24,724 | 111 | 87 | 1,183 | 1,184 | 7,203 | 6,997 | 65,982 |
| HET-HOM | 7 | 24,431 | 24,410 | 109 | 101 | 1,074 | 1,101 | 7,067 | 6,914 | 65,207 |
| Total | Total | 168,684 | 170,049 | 759 | 674 | 7,957 | 7,880 | 50,074 | 48,776 | 454,853 |
