# Supplementary material for: Juxtaposition of heterozygous and homozygous regions causes reciprocal crossover remodelling via interference during Arabidopsis meiosis
Source: eLife. 2015 Mar 27;4:e03708. doi: 10.7554/eLife.03708 (PMC4407271; doi:10.7554/eLife.03708)
Supplement: Figure 5—source data 2. — DOI: http://dx.doi.org/10.7554/eLife.03708.026 [file elife03708s011.docx]

**Figure 5 – Source Data 2. Three colour *I3bc* FTL flow cytometry count data –measurement of crossover interference.** For the formula used for cM calculation please see Materials and Methods.

| Genotype | Replicate | *I3b* cM | *I3c* cM | Expected DCOs | Observed DCOs | Interference |
| --- | --- | --- | --- | --- | --- | --- |
| HOM-HOM | 1 | 18.22 | 5.24 | 263 | 112 | 0.574 |
| HOM-HOM | 2 | 18.12 | 5.06 | 634 | 229 | 0.639 |
| HOM-HOM | 3 | 18.62 | 5.24 | 592 | 224 | 0.622 |
| HOM-HOM | 4 | 18.66 | 5.44 | 1,277 | 439 | 0.656 |
| HOM-HOM | 5 | 16.08 | 5.06 | 225 | 99 | 0.560 |
| HOM-HOM | 6 | 16.13 | 5.18 | 278 | 106 | 0.619 |
| HOM-HOM | 7 | 16.17 | 5.00 | 650 | 281 | 0.568 |
| HOM-HOM | Total | 17.70 | 5.21 | 3,913 | 1,490 | 0.619 |
| HET-HOM | 1 | 22.46 | 3.89 | 564 | 212 | 0.624 |
| HET-HOM | 2 | 21.63 | 3.44 | 315 | 114 | 0.638 |
| HET-HOM | 3 | 23.03 | 3.85 | 525 | 198 | 0.623 |
| HET-HOM | 4 | 22.21 | 3.92 | 726 | 258 | 0.644 |
| HET-HOM | 5 | 21.40 | 3.78 | 601 | 243 | 0.596 |
| HET-HOM | 6 | 21.82 | 3.89 | 560 | 198 | 0.646 |
| HET-HOM | 7 | 21.76 | 3.66 | 519 | 210 | 0.595 |
| HET-HOM | Total | 22.05 | 3.80 | 3808 | 1,433 | 0.624 |
