# Supplementary material for: Juxtaposition of heterozygous and homozygous regions causes reciprocal crossover remodelling via interference during Arabidopsis meiosis
Source: eLife. 2015 Mar 27;4:e03708. doi: 10.7554/eLife.03708 (PMC4407271; doi:10.7554/eLife.03708)
Supplement: Figure 6—source data 1. — DOI: http://dx.doi.org/10.7554/eLife.03708.030 [file elife03708s012.docx]

**Figure 6 – Source Data 1. *420* fluorescent seed count data from wild type, *fancm* and *fancm zip4* individuals with varying heterozygosity.** For the formula used for cM calculation please see Materials and Methods. The genotype column indicates whether the individual was wild type, *fancm* or *fancm zip4*. The polymorphism column lists whether the regions within and outside *420* were homozygous (HOM) or heterozygous (HET).

| Genotype | Polymorphism | Red alone | Green alone | Neither | Red and Green | Total  Seed | cM | Red:non-Red | Green:non-Green | Red:Green |
| --- | --- | --- | --- | --- | --- | --- | --- | --- | --- | --- |
| wild type | HOM-HOM | 285 | 256 | 2000 | 494 | 3035 | 19.78 | 3.05 | 2.90 | 1.11 |
| wild type | HOM-HOM | 257 | 293 | 1962 | 556 | 3068 | 19.91 | 2.61 | 2.77 | 0.88 |
| wild type | HOM-HOM | 261 | 256 | 1942 | 519 | 2978 | 19.20 | 2.84 | 2.82 | 1.02 |
| wild type | HOM-HOM | 260 | 279 | 2017 | 508 | 3064 | 19.49 | 2.89 | 2.99 | 0.93 |
| wild type | HOM-HOM | 236 | 249 | 1893 | 517 | 2895 | 18.46 | 2.78 | 2.84 | 0.95 |
| wild type | HET-HOM | 375 | 303 | 1714 | 415 | 2807 | 28.10 | 2.91 | 2.55 | 1.24 |
| wild type | HET-HOM | 345 | 306 | 1683 | 352 | 2686 | 28.22 | 3.08 | 2.85 | 1.13 |
| wild type | HET-HOM | 334 | 348 | 1676 | 339 | 2697 | 29.70 | 2.93 | 3.01 | 0.96 |
| wild type | HET-HOM | 303 | 327 | 1526 | 360 | 2516 | 29.35 | 2.66 | 2.79 | 0.93 |
| wild type | HET-HOM | 306 | 335 | 1736 | 404 | 2781 | 26.58 | 2.76 | 2.92 | 0.91 |
| wild type | HET-HOM | 307 | 309 | 1727 | 376 | 2719 | 26.05 | 2.97 | 2.98 | 0.99 |
| wild type | HET-HOM | 313 | 289 | 1794 | 367 | 2763 | 24.88 | 3.21 | 3.06 | 1.08 |
| wild type | HET-HOM | 328 | 312 | 1760 | 372 | 2772 | 26.64 | 3.05 | 2.96 | 1.05 |
| wild type | HET-HOM | 329 | 333 | 1789 | 367 | 2818 | 27.19 | 3.03 | 3.05 | 0.99 |
| wild type | HET-HOM | 303 | 309 | 1618 | 356 | 2586 | 27.43 | 2.89 | 2.92 | 0.98 |
| wild type | HET-HOM | 370 | 333 | 1808 | 402 | 2913 | 28.07 | 2.96 | 2.77 | 1.11 |
| wild type | HET-HOM | 336 | 308 | 1796 | 387 | 2827 | 26.22 | 3.07 | 2.91 | 1.09 |
| wild type | HET-HOM | 340 | 312 | 1809 | 365 | 2826 | 26.61 | 3.17 | 3.01 | 1.09 |
| wild type | HET-HOM | 282 | 347 | 1700 | 382 | 2711 | 26.79 | 2.72 | 3.08 | 0.81 |
| wild type | HET-HOM | 314 | 300 | 1868 | 419 | 2901 | 24.06 | 3.03 | 2.96 | 1.05 |
| wild type | HET-HOM | 373 | 305 | 1827 | 424 | 2929 | 26.72 | 3.02 | 2.68 | 1.22 |
| wild type | HOM-HET | 148 | 157 | 1611 | 471 | 2387 | 13.72 | 2.80 | 2.86 | 0.94 |
| wild type | HOM-HET | 143 | 149 | 1678 | 446 | 2416 | 12.92 | 3.06 | 3.10 | 0.96 |
| wild type | HOM-HET | 144 | 147 | 1735 | 473 | 2499 | 12.42 | 3.03 | 3.05 | 0.98 |
| wild type | HOM-HET | 157 | 162 | 1738 | 474 | 2531 | 13.52 | 2.98 | 3.01 | 0.97 |
| wild type | HOM-HET | 177 | 189 | 1693 | 437 | 2496 | 15.93 | 2.99 | 3.07 | 0.94 |
| wild type | HOM-HET | 109 | 125 | 1151 | 327 | 1712 | 14.76 | 2.79 | 2.93 | 0.87 |
| wild type | HOM-HET | 160 | 152 | 1653 | 451 | 2416 | 13.88 | 3.01 | 2.95 | 1.05 |
| wild type | HOM-HET | 165 | 201 | 1770 | 484 | 2620 | 15.11 | 2.82 | 3.04 | 0.82 |
| wild type | HOM-HET | 215 | 217 | 1822 | 464 | 2718 | 17.41 | 2.99 | 3.00 | 0.99 |
| wild type | HOM-HET | 201 | 204 | 1758 | 433 | 2596 | 17.06 | 3.08 | 3.09 | 0.99 |
| wild type | HOM-HET | 190 | 209 | 1715 | 467 | 2581 | 16.88 | 2.82 | 2.93 | 0.91 |
| wild type | HET-HET | 234 | 215 | 1726 | 449 | 2624 | 18.90 | 2.95 | 2.84 | 1.09 |
| wild type | HET-HET | 318 | 229 | 1772 | 405 | 2724 | 22.64 | 3.30 | 2.77 | 1.39 |
| wild type | HET-HET | 238 | 236 | 1812 | 489 | 2775 | 18.86 | 2.83 | 2.82 | 1.01 |
| wild type | HET-HET | 210 | 200 | 1366 | 351 | 2127 | 21.61 | 2.86 | 2.79 | 1.05 |
| wild type | HET-HET | 246 | 255 | 1750 | 464 | 2715 | 20.57 | 2.78 | 2.82 | 0.96 |
| wild type | HET-HET | 265 | 274 | 1776 | 508 | 2823 | 21.38 | 2.61 | 2.65 | 0.97 |
| wild type | HET-HET | 224 | 225 | 1663 | 442 | 2554 | 19.48 | 2.83 | 2.83 | 1.00 |
| wild type | HET-HET | 261 | 282 | 1737 | 457 | 2737 | 22.33 | 2.70 | 2.81 | 0.93 |
| wild type | HET-HET | 201 | 195 | 1529 | 380 | 2305 | 18.98 | 3.01 | 2.97 | 1.03 |
| wild type | HET-HET | 221 | 216 | 1722 | 397 | 2556 | 18.88 | 3.17 | 3.14 | 1.02 |
| wild type | HET-HET | 278 | 263 | 1757 | 409 | 2707 | 22.52 | 3.03 | 2.94 | 1.06 |
| wild type | HET-HET | 268 | 279 | 1679 | 420 | 2646 | 23.41 | 2.79 | 2.85 | 0.96 |
| wild type | HET-HET | 241 | 283 | 1741 | 417 | 2682 | 21.95 | 2.83 | 3.08 | 0.85 |
| wild type | HET-HET | 271 | 265 | 1911 | 461 | 2908 | 20.54 | 3.01 | 2.97 | 1.02 |
| wild type | HET-HET | 267 | 278 | 1814 | 420 | 2779 | 22.04 | 2.98 | 3.05 | 0.96 |
| wild type | HET-HET | 261 | 250 | 1744 | 485 | 2740 | 20.82 | 2.73 | 2.67 | 1.04 |
| wild type | HET-HET | 253 | 261 | 1927 | 479 | 2920 | 19.50 | 2.95 | 2.99 | 0.97 |
| wild type | HET-HET | 252 | 253 | 1598 | 448 | 2551 | 22.28 | 2.64 | 2.64 | 1.00 |
| *fancm* | HOM-HOM | 211 | 170 | 1723 | 482 | 2586 | 16.02 | 2.97 | 2.73 | 1.24 |
| *fancm* | HOM-HOM | 188 | 162 | 1734 | 464 | 2548 | 14.84 | 3.07 | 2.91 | 1.16 |
| *fancm* | HOM-HOM | 205 | 206 | 1771 | 449 | 2631 | 17.08 | 3.02 | 3.02 | 1.00 |
| *fancm* | HOM-HOM | 199 | 207 | 1770 | 472 | 2648 | 16.73 | 2.90 | 2.95 | 0.96 |
| *fancm* | HOM-HOM | 208 | 193 | 1711 | 483 | 2595 | 16.88 | 2.84 | 2.76 | 1.08 |
| *fancm* | HOM-HOM | 377 | 375 | 1521 | 279 | 2552 | 35.92 | 2.90 | 2.89 | 1.01 |
| *fancm* | HOM-HOM | 410 | 350 | 1487 | 271 | 2518 | 37.04 | 3.05 | 2.70 | 1.17 |
| *fancm* | HOM-HOM | 262 | 273 | 1082 | 189 | 1806 | 36.16 | 2.91 | 3.00 | 0.96 |
| *fancm* | HOM-HOM | 359 | 375 | 1422 | 237 | 2393 | 37.83 | 2.91 | 3.02 | 0.96 |
| *fancm* | HOM-HOM | 391 | 341 | 1476 | 267 | 2475 | 36.09 | 3.07 | 2.76 | 1.15 |
| *fancm* | HOM-HOM | 349 | 354 | 1459 | 276 | 2438 | 34.94 | 2.87 | 2.90 | 0.99 |
| *fancm* | HET-HOM | 374 | 324 | 1622 | 331 | 2651 | 31.20 | 3.05 | 2.76 | 1.15 |
| *fancm* | HET-HOM | 343 | 325 | 1628 | 333 | 2629 | 29.87 | 3.00 | 2.89 | 1.06 |
| *fancm* | HET-HOM | 355 | 372 | 1658 | 345 | 2730 | 31.63 | 2.81 | 2.90 | 0.95 |
| *fancm* | HET-HOM | 340 | 323 | 1664 | 338 | 2665 | 29.12 | 3.03 | 2.93 | 1.05 |
| *fancm* | HET-HOM | 330 | 329 | 1608 | 344 | 2611 | 29.63 | 2.88 | 2.87 | 1.00 |
| *fancm* | HET-HOM | 364 | 328 | 1621 | 335 | 2648 | 30.91 | 2.99 | 2.79 | 1.11 |
| *fancm* | HET-HOM | 357 | 342 | 1717 | 308 | 2724 | 30.23 | 3.19 | 3.10 | 1.04 |
| *fancm* | HET-HOM | 313 | 293 | 1406 | 299 | 2311 | 31.04 | 2.90 | 2.78 | 1.07 |
| *fancm* | HET-HOM | 336 | 324 | 1755 | 371 | 2786 | 27.46 | 3.01 | 2.94 | 1.04 |
| *fancm* | HET-HOM | 347 | 343 | 1704 | 308 | 2702 | 30.05 | 3.15 | 3.13 | 1.01 |
| *fancm* | HET-HOM | 385 | 343 | 1614 | 337 | 2679 | 32.43 | 2.94 | 2.71 | 1.12 |
| *fancm* | HET-HOM | 375 | 412 | 1693 | 354 | 2834 | 33.32 | 2.70 | 2.89 | 0.91 |
| *fancm* | HET-HOM | 313 | 293 | 1406 | 299 | 2311 | 31.04 | 2.90 | 2.78 | 1.07 |
| *fancm* | HET-HOM | 336 | 324 | 1755 | 371 | 2786 | 27.46 | 3.01 | 2.94 | 1.04 |
| *fancm* | HOM-HET | 378 | 349 | 1656 | 301 | 2684 | 32.30 | 3.13 | 2.95 | 1.08 |
| *fancm* | HOM-HET | 367 | 397 | 1627 | 382 | 2773 | 32.99 | 2.56 | 2.70 | 0.92 |
| *fancm* | HOM-HET | 381 | 393 | 1689 | 289 | 2752 | 33.86 | 3.04 | 3.11 | 0.97 |
| *fancm* | HOM-HET | 418 | 423 | 1718 | 276 | 2835 | 36.23 | 3.06 | 3.09 | 0.99 |
| *fancm* | HOM-HET | 376 | 381 | 1692 | 320 | 2769 | 32.68 | 2.95 | 2.98 | 0.99 |
| *fancm* | HOM-HET | 366 | 414 | 1717 | 314 | 2811 | 33.29 | 2.86 | 3.13 | 0.88 |
| *fancm* | HOM-HET | 378 | 414 | 1783 | 300 | 2875 | 32.99 | 3.03 | 3.24 | 0.91 |
| *fancm* | HOM-HET | 399 | 384 | 1703 | 259 | 2745 | 34.46 | 3.27 | 3.17 | 1.04 |
| *fancm* | HOM-HET | 385 | 432 | 1735 | 286 | 2838 | 34.87 | 2.95 | 3.23 | 0.89 |
| *fancm* | HOM-HET | 364 | 354 | 1648 | 305 | 2671 | 32.00 | 3.05 | 2.99 | 1.03 |
| *fancm* | HET-HET | 303 | 256 | 1682 | 379 | 2620 | 24.28 | 3.13 | 2.84 | 1.18 |
| *fancm* | HET-HET | 338 | 310 | 1741 | 386 | 2775 | 27.00 | 2.99 | 2.83 | 1.09 |
| *fancm* | HET-HET | 288 | 297 | 1594 | 402 | 2581 | 26.06 | 2.69 | 2.74 | 0.97 |
| *fancm* | HET-HET | 322 | 268 | 1683 | 372 | 2645 | 25.58 | 3.13 | 2.81 | 1.20 |
| *fancm* | HET-HET | 277 | 263 | 1767 | 413 | 2720 | 22.35 | 3.02 | 2.94 | 1.05 |
| *fancm* | HET-HET | 281 | 243 | 1618 | 371 | 2513 | 23.65 | 3.09 | 2.85 | 1.16 |
| *fancm* | HET-HET | 249 | 261 | 1733 | 404 | 2647 | 21.60 | 2.98 | 3.05 | 0.95 |
| *fancm* | HET-HET | 220 | 229 | 1486 | 395 | 2330 | 21.60 | 2.73 | 2.79 | 0.96 |
| *fancm* | HET-HET | 261 | 226 | 1594 | 418 | 2499 | 21.88 | 2.88 | 2.68 | 1.15 |
| *fancm* | HET-HET | 263 | 279 | 1827 | 461 | 2830 | 21.45 | 2.82 | 2.91 | 0.94 |
| *fancm* | HET-HET | 303 | 287 | 1777 | 444 | 2811 | 23.83 | 2.85 | 2.76 | 1.06 |
| *fancm* | HET-HET | 262 | 257 | 1723 | 385 | 2627 | 22.23 | 3.09 | 3.06 | 1.02 |
| *fancm* | HET-HET | 290 | 298 | 1739 | 388 | 2715 | 24.71 | 2.96 | 3.00 | 0.97 |
| *fancm* | HET-HET | 318 | 316 | 1824 | 469 | 2927 | 24.71 | 2.73 | 2.72 | 1.01 |
| *fancm* | HET-HET | 284 | 307 | 1752 | 438 | 2781 | 24.17 | 2.73 | 2.85 | 0.93 |
| *fancm* | HET-HET | 289 | 303 | 1719 | 457 | 2768 | 24.35 | 2.64 | 2.71 | 0.95 |
| *fancm* | HET-HET | 306 | 250 | 1564 | 368 | 2488 | 25.63 | 3.03 | 2.69 | 1.22 |
| *fancm zip4* | HOM-HOM | 345 | 380 | 1588 | 278 | 2591 | 33.64 | 2.94 | 3.16 | 0.91 |
| *fancm zip4* | HOM-HOM | 381 | 402 | 1689 | 334 | 2806 | 33.52 | 2.81 | 2.92 | 0.95 |
| *fancm zip4* | HOM-HOM | 357 | 353 | 1583 | 271 | 2564 | 33.20 | 3.11 | 3.08 | 1.01 |
| *fancm zip4* | HOM-HOM | 362 | 376 | 1558 | 270 | 2566 | 34.82 | 2.97 | 3.06 | 0.96 |
| *fancm zip4* | HOM-HOM | 445 | 395 | 1619 | 311 | 2770 | 37.27 | 2.92 | 2.66 | 1.13 |
| *fancm zip4* | HOM-HOM | 362 | 362 | 1562 | 291 | 2577 | 33.81 | 2.95 | 2.95 | 1.00 |
| *fancm zip4* | HET-HOM | 173 | 138 | 1698 | 507 | 2516 | 13.24 | 2.90 | 2.70 | 1.25 |
| *fancm zip4* | HET-HOM | 127 | 155 | 1857 | 528 | 2667 | 11.20 | 2.90 | 3.07 | 0.82 |
| *fancm zip4* | HET-HOM | 149 | 127 | 1851 | 543 | 2670 | 10.93 | 2.99 | 2.86 | 1.17 |
| *fancm zip4* | HET-HOM | 134 | 126 | 1643 | 502 | 2405 | 11.47 | 2.83 | 2.78 | 1.06 |
| *fancm zip4* | HET-HOM | 125 | 157 | 1712 | 517 | 2511 | 11.94 | 2.73 | 2.91 | 0.80 |
| *fancm zip4* | HET-HOM | 123 | 147 | 1800 | 533 | 2603 | 10.97 | 2.83 | 2.97 | 0.84 |
| *fancm zip4* | HET-HOM | 124 | 143 | 1708 | 512 | 2487 | 11.38 | 2.80 | 2.91 | 0.87 |
| *fancm zip4* | HET-HOM | 124 | 147 | 1806 | 526 | 2603 | 11.02 | 2.87 | 3.00 | 0.84 |
| *fancm zip4* | HET-HOM | 126 | 143 | 1870 | 549 | 2688 | 10.57 | 2.88 | 2.98 | 0.88 |
| *fancm zip4* | HET-HOM | 153 | 132 | 1770 | 532 | 2587 | 11.70 | 2.90 | 2.78 | 1.16 |
| *fancm zip4* | HET-HOM | 114 | 113 | 1877 | 595 | 2699 | 8.80 | 2.81 | 2.81 | 1.01 |
| *fancm zip4* | HET-HOM | 164 | 150 | 1923 | 545 | 2782 | 12.01 | 3.00 | 2.92 | 1.09 |
| *fancm zip4* | HET-HOM | 130 | 135 | 1940 | 535 | 2740 | 10.19 | 3.09 | 3.12 | 0.96 |
| *fancm zip4* | HET-HOM | 160 | 181 | 1846 | 587 | 2774 | 13.16 | 2.61 | 2.71 | 0.88 |
| *fancm zip4* | HET-HOM | 155 | 166 | 1882 | 549 | 2752 | 12.44 | 2.85 | 2.91 | 0.93 |
| *fancm zip4* | HET-HOM | 153 | 141 | 1780 | 547 | 2621 | 11.93 | 2.81 | 2.74 | 1.09 |
| *fancm zip4* | HOM-HET | 329 | 327 | 1352 | 203 | 2211 | 36.23 | 3.17 | 3.16 | 1.01 |
| *fancm zip4* | HOM-HET | 181 | 190 | 758 | 111 | 1240 | 36.63 | 3.12 | 3.25 | 0.95 |
| *fancm zip4* | HOM-HET | 362 | 339 | 1461 | 228 | 2390 | 35.70 | 3.22 | 3.05 | 1.07 |
| *fancm zip4* | HOM-HET | 391 | 357 | 1408 | 229 | 2385 | 38.95 | 3.07 | 2.85 | 1.10 |
| *fancm zip4* | HOM-HET | 371 | 395 | 1513 | 219 | 2498 | 37.81 | 3.07 | 3.23 | 0.94 |
| *fancm zip4* | HOM-HET | 364 | 399 | 1520 | 215 | 2498 | 37.62 | 3.07 | 3.31 | 0.91 |
| *fancm zip4* | HOM-HET | 390 | 357 | 1408 | 230 | 2385 | 38.88 | 3.06 | 2.85 | 1.09 |
| *fancm zip4* | HOM-HET | 189 | 206 | 765 | 152 | 1312 | 36.92 | 2.66 | 2.85 | 0.92 |
| *fancm zip4* | HOM-HET | 283 | 278 | 1081 | 203 | 1845 | 37.40 | 2.84 | 2.80 | 1.02 |
| *fancm zip4* | HOM-HET | 287 | 295 | 1122 | 206 | 1910 | 37.50 | 2.81 | 2.87 | 0.97 |
| *fancm zip4* | HOM-HET | 242 | 283 | 1013 | 207 | 1745 | 36.89 | 2.56 | 2.89 | 0.86 |
| *fancm zip4* | HOM-HET | 165 | 179 | 717 | 167 | 1228 | 33.69 | 2.55 | 2.70 | 0.92 |
| *fancm zip4* | HET-HET | 127 | 129 | 1475 | 449 | 2180 | 12.53 | 2.77 | 2.78 | 0.98 |
| *fancm zip4* | HET-HET | 191 | 169 | 1666 | 452 | 2478 | 15.77 | 2.99 | 2.85 | 1.13 |
| *fancm zip4* | HET-HET | 160 | 168 | 1689 | 413 | 2430 | 14.56 | 3.18 | 3.24 | 0.95 |
| *fancm zip4* | HET-HET | 136 | 143 | 1520 | 378 | 2177 | 13.76 | 3.18 | 3.24 | 0.95 |
| *fancm zip4* | HET-HET | 165 | 134 | 1725 | 414 | 2438 | 13.13 | 3.45 | 3.21 | 1.23 |
| *fancm zip4* | HET-HET | 165 | 156 | 1653 | 437 | 2411 | 14.34 | 3.07 | 3.00 | 1.06 |
| *fancm zip4* | HET-HET | 100 | 99 | 1235 | 335 | 1769 | 11.97 | 3.08 | 3.07 | 1.01 |
| *fancm zip4* | HET-HET | 152 | 140 | 1610 | 439 | 2341 | 13.37 | 3.04 | 2.96 | 1.09 |
| *fancm zip4* | HET-HET | 156 | 134 | 1567 | 393 | 2250 | 13.85 | 3.27 | 3.10 | 1.16 |
| *fancm zip4* | HET-HET | 157 | 139 | 1532 | 430 | 2258 | 14.10 | 2.97 | 2.85 | 1.13 |
| *fancm zip4* | HET-HET | 161 | 165 | 1706 | 472 | 2504 | 14.00 | 2.93 | 2.96 | 0.98 |
| *fancm zip4* | HET-HET | 164 | 150 | 1687 | 441 | 2442 | 13.81 | 3.13 | 3.04 | 1.09 |
| *fancm zip4* | HET-HET | 142 | 159 | 1600 | 494 | 2395 | 13.48 | 2.67 | 2.77 | 0.89 |
| *fancm zip4* | HET-HET | 153 | 141 | 1708 | 464 | 2466 | 12.73 | 3.08 | 3.00 | 1.09 |
| *fancm zip4* | HET-HET | 180 | 148 | 1616 | 472 | 2416 | 14.65 | 2.90 | 2.71 | 1.22 |
