# Supplementary material for: Juxtaposition of heterozygous and homozygous regions causes reciprocal crossover remodelling via interference during Arabidopsis meiosis
Source: eLife. 2015 Mar 27;4:e03708. doi: 10.7554/eLife.03708 (PMC4407271; doi:10.7554/eLife.03708)
Supplement: Figure 6—source data 2. — DOI: http://dx.doi.org/10.7554/eLife.03708.031 [file elife03708s013.docx]

**Figure 6 – Source Data 2. *CEN3* flow cytometry count data from wild type, *fancm* and *fancm zip4* individuals with varying heterozygosity.** cM were calculated as 100 x R5/(R3+R5). The genotype column indicates whether the individual was wild type, *fancm* or *fancm zip4*. The polymorphism column lists whether the regions within and outside *420* were homozygous (HOM) or heterozygous (HET).

| Genotype | Polymorphism | Red alone (Gate R2) | Red and Green (Gate R3) | Neither (Gate R4) | Green alone (Gate R5) | cM |
| --- | --- | --- | --- | --- | --- | --- |
| wild type | HOM-HOM | 1093 | 5570 | 7883 | 775 | 12.21 |
| wild type | HOM-HOM | 646 | 3389 | 4790 | 461 | 11.97 |
| wild type | HOM-HOM | 718 | 4039 | 5031 | 495 | 10.92 |
| wild type | HET-HOM | 551 | 2558 | 3656 | 256 | 9.10 |
| wild type | HET-HOM | 964 | 5663 | 6706 | 531 | 8.57 |
| wild type | HET-HOM | 544 | 2541 | 3847 | 251 | 8.99 |
| wild type | HET-HOM | 1082 | 3000 | 5991 | 265 | 8.12 |
| wild type | HET-HOM | 1048 | 3076 | 5914 | 264 | 7.90 |
| wild type | HET-HOM | 1150 | 3433 | 7125 | 354 | 9.35 |
| wild type | HET-HOM | 1034 | 2967 | 6710 | 311 | 9.49 |
| wild type | HET-HOM | 1213 | 4991 | 8414 | 504 | 9.17 |
| wild type | HET-HOM | 1279 | 6437 | 9821 | 593 | 8.44 |
| wild type | HET-HOM | 1017 | 4484 | 7086 | 500 | 10.03 |
| wild type | HOM-HET | 334 | 1979 | 2572 | 255 | 11.41 |
| wild type | HOM-HET | 285 | 1077 | 1878 | 138 | 11.36 |
| wild type | HOM-HET | 745 | 3644 | 5168 | 456 | 11.12 |
| wild type | HOM-HET | 616 | 2066 | 4234 | 297 | 12.57 |
| wild type | HOM-HET | 394 | 1585 | 2444 | 232 | 12.77 |
| wild type | HET-HET | 1136 | 4054 | 7446 | 502 | 11.02 |
| wild type | HET-HET | 1410 | 5070 | 8538 | 659 | 11.50 |
| wild type | HET-HET | 1412 | 4973 | 7525 | 514 | 9.37 |
| wild type | HET-HET | 909 | 4267 | 7013 | 454 | 9.62 |
| wild type | HET-HET | 807 | 4688 | 6501 | 496 | 9.57 |
| wild type | HET-HET | 594 | 2692 | 4337 | 248 | 8.44 |
| wild type | HET-HET | 746 | 4596 | 5454 | 492 | 9.67 |
| wild type | HET-HET | 545 | 3223 | 4330 | 388 | 10.74 |
| wild type | HET-HET | 708 | 3971 | 5205 | 481 | 10.80 |
| *fancm* | HOM-HOM | 751 | 3208 | 4260 | 551 | 14.66 |
| *fancm* | HOM-HOM | 877 | 3778 | 4826 | 633 | 14.35 |
| *fancm* | HOM-HOM | 1536 | 4332 | 8541 | 785 | 15.34 |
| *fancm* | HOM-HOM | 1634 | 4399 | 9063 | 788 | 15.19 |
| *fancm* | HET-HOM | 746 | 2534 | 4021 | 475 | 15.79 |
| *fancm* | HET-HOM | 541 | 1717 | 2946 | 324 | 15.87 |
| *fancm* | HET-HOM | 1220 | 3173 | 7077 | 554 | 14.86 |
| *fancm* | HET-HOM | 1388 | 3496 | 7470 | 672 | 16.12 |
| *fancm* | HET-HOM | 1075 | 2809 | 6332 | 480 | 14.59 |
| *fancm* | HET-HOM | 2020 | 5980 | 10780 | 1096 | 15.49 |
| *fancm* | HET-HOM | 666 | 2019 | 3277 | 305 | 13.12 |
| *fancm* | HET-HOM | 519 | 1231 | 2842 | 238 | 16.20 |
| *fancm* | HET-HOM | 950 | 1620 | 3744 | 333 | 17.05 |
| *fancm* | HOM-HET | 406 | 1989 | 2776 | 217 | 9.84 |
| *fancm* | HOM-HET | 502 | 2526 | 3560 | 265 | 9.49 |
| *fancm* | HOM-HET | 414 | 2229 | 3254 | 230 | 9.35 |
| *fancm* | HOM-HET | 609 | 2241 | 3247 | 259 | 10.36 |
| *fancm* | HOM-HET | 891 | 3844 | 5145 | 412 | 9.68 |
| *fancm* | HOM-HET | 2059 | 10124 | 13563 | 1148 | 10.18 |
| *fancm* | HOM-HET | 1912 | 10159 | 13534 | 1126 | 9.98 |
| *fancm* | HOM-HET | 2805 | 10124 | 17280 | 1242 | 10.93 |
| *fancm* | HET-HET | 948 | 4276 | 7046 | 457 | 9.66 |
| *fancm* | HET-HET | 580 | 3547 | 5308 | 342 | 8.79 |
| *fancm* | HET-HET | 900 | 4165 | 6676 | 508 | 10.87 |
| *fancm* | HET-HET | 566 | 3506 | 4587 | 385 | 9.89 |
| *fancm* | HET-HET | 614 | 4000 | 5412 | 438 | 9.87 |
| *fancm* | HET-HET | 664 | 4351 | 5579 | 432 | 9.03 |
| *fancm* | HET-HET | 629 | 3160 | 4473 | 325 | 9.33 |
| *fancm* | HET-HET | 630 | 3932 | 4991 | 486 | 11.00 |
| *fancm* | HET-HET | 688 | 3776 | 5209 | 383 | 9.21 |
| *fancm zip4* | HOM-HOM | 807 | 5421 | 6745 | 412 | 7.06 |
| *fancm zip4* | HOM-HOM | 535 | 4571 | 5156 | 342 | 6.96 |
| *fancm zip4* | HOM-HOM | 451 | 3120 | 4099 | 229 | 6.84 |
| *fancm zip4* | HOM-HOM | 665 | 4808 | 6199 | 375 | 7.24 |
| *fancm zip4* | HOM-HOM | 486 | 3253 | 4519 | 289 | 8.16 |
| *fancm zip4* | HET-HOM | 361 | 1635 | 2777 | 128 | 7.26 |
| *fancm zip4* | HET-HOM | 367 | 1481 | 2847 | 120 | 7.50 |
| *fancm zip4* | HET-HOM | 568 | 2147 | 4855 | 187 | 8.01 |
| *fancm zip4* | HET-HOM | 595 | 1935 | 4286 | 163 | 7.77 |
| *fancm zip4* | HET-HOM | 981 | 3089 | 7105 | 265 | 7.90 |
| *fancm zip4* | HET-HOM | 898 | 3454 | 7185 | 298 | 7.94 |
| *fancm zip4* | HET-HOM | 907 | 3470 | 7302 | 320 | 8.44 |
| *fancm zip4* | HET-HOM | 1092 | 4657 | 7875 | 394 | 7.80 |
| *fancm zip4* | HET-HOM | 987 | 4188 | 7139 | 374 | 8.20 |
| *fancm zip4* | HET-HOM | 1016 | 4513 | 7636 | 429 | 8.68 |
| *fancm zip4* | HOM-HET | 902 | 10155 | 14182 | 86 | 0.84 |
| *fancm zip4* | HOM-HET | 834 | 5773 | 10702 | 37 | 0.64 |
| *fancm zip4* | HOM-HET | 803 | 7046 | 9904 | 51 | 0.72 |
| *fancm zip4* | HOM-HET | 380 | 1404 | 2904 | 16 | 1.13 |
| *fancm zip4* | HOM-HET | 521 | 1505 | 3211 | 15 | 0.99 |
| *fancm zip4* | HET-HET | 685 | 3060 | 4972 | 49 | 1.58 |
| *fancm zip4* | HET-HET | 500 | 2091 | 3498 | 26 | 1.23 |
| *fancm zip4* | HET-HET | 647 | 2970 | 5185 | 35 | 1.16 |
| *fancm zip4* | HET-HET | 423 | 3423 | 4882 | 23 | 0.67 |
| *fancm zip4* | HET-HET | 458 | 3223 | 4962 | 25 | 0.77 |
| *fancm zip4* | HET-HET | 439 | 3606 | 5599 | 21 | 0.58 |
| *fancm zip4* | HET-HET | 442 | 3597 | 5758 | 41 | 1.13 |
| *fancm zip4* | HET-HET | 372 | 2530 | 4427 | 31 | 1.21 |
| *fancm zip4* | HET-HET | 262 | 1946 | 3733 | 22 | 1.12 |
