# Supplementary material for: Juxtaposition of heterozygous and homozygous regions causes reciprocal crossover remodelling via interference during Arabidopsis meiosis
Source: eLife. 2015 Mar 27;4:e03708. doi: 10.7554/eLife.03708 (PMC4407271; doi:10.7554/eLife.03708)
Supplement: Figure 7—source data 1. — DOI: http://dx.doi.org/10.7554/eLife.03708.034 [file elife03708s014.docx]

**Figure 7 – Source Data 1. Chiasmata count data.** Chiasmata were counted from metaphase-I spreads labeled with *45S* rDNA FISH probes. The cold and hot *420* lines correspond to recombinant lines with significantly elevated recombination relative to Col/Col homozygous lines (cM) (Fig. 6).

| Chiasmata | Col/Col | Ct/Ct | Col/Ct F_1_ | HOM-HET *420* | HET-HOM *420* |
| --- | --- | --- | --- | --- | --- |
| 6 | 0 | 0 | 2 | 1 | 0 |
| 7 | 6 | 0 | 4 | 4 | 3 |
| 8 | 7 | 12 | 7 | 15 | 14 |
| 9 | 16 | 12 | 14 | 20 | 13 |
| 10 | 6 | 13 | 3 | 13 | 6 |
| 11 | 0 | 0 | 0 | 2 | 0 |
