# Supplementary material for: Juxtaposition of heterozygous and homozygous regions causes reciprocal crossover remodelling via interference during Arabidopsis meiosis
Source: eLife. 2015 Mar 27;4:e03708. doi: 10.7554/eLife.03708 (PMC4407271; doi:10.7554/eLife.03708)
Supplement: Figure 8—source data 1. — DOI: http://dx.doi.org/10.7554/eLife.03708.036 [file elife03708s015.docx]

**Figure 8 – Source Data 1. *I3bc* fluorescent seed count data from wild type, *fancm* and *fancm zip4* individuals with varying heterozygosity.** Counts are listed for the 8 pollen fluorescent classes (Fig. 5 – Figure Supplement 1).

| Heterozygosity | Genotype | BYR | byr | bYr | ByR | BYr | byR | bYR | Byr | Total |
| --- | --- | --- | --- | --- | --- | --- | --- | --- | --- | --- |
| HOM-HOM | wild type | 7127 | 7447 | 26 | 21 | 397 | 474 | 1476 | 1476 | 18444 |
| HOM-HOM | wild type | 9115 | 9513 | 38 | 30 | 482 | 467 | 1765 | 1817 | 23227 |
| HOM-HOM | wild type | 9491 | 9993 | 36 | 32 | 538 | 613 | 1796 | 1899 | 24398 |
| HOM-HOM | Total | 25733 | 26953 | 100 | 83 | 1417 | 1554 | 5037 | 5192 | 66069 |
| HOM-HOM | *fancm* | 9493 | 9886 | 604 | 624 | 1835 | 1693 | 3285 | 4301 | 31721 |
| HOM-HOM | *fancm* | 9041 | 9926 | 557 | 565 | 1639 | 1545 | 3330 | 4391 | 30994 |
| HOM-HOM | *fancm* | 7917 | 8708 | 466 | 421 | 1465 | 1363 | 2858 | 3608 | 26806 |
| HOM-HOM | *fancm* | 3754 | 3799 | 256 | 200 | 635 | 633 | 1570 | 1771 | 12618 |
| HOM-HOM | Total | 30205 | 32319 | 1883 | 1810 | 5574 | 5234 | 11043 | 14071 | 102139 |
| HOM-HOM | *fancm/zip4* | 11368 | 11832 | 831 | 821 | 1666 | 1567 | 5101 | 5356 | 38542 |
| HOM-HOM | *fancm/zip4* | 6311 | 5944 | 295 | 297 | 833 | 796 | 2175 | 1898 | 18549 |
| HOM-HOM | *fancm/zip4* | 9745 | 11066 | 507 | 480 | 1476 | 1443 | 2858 | 3720 | 31295 |
| HOM-HOM | Total | 27424 | 28842 | 1633 | 1598 | 3975 | 3806 | 10134 | 10974 | 88386 |
| HET-HET | wild type | 36244 | 42935 | 63 | 42 | 2441 | 2250 | 6832 | 8408 | 99215 |
| HET-HET | wild type | 31277 | 36438 | 68 | 47 | 2065 | 2002 | 6114 | 7356 | 85367 |
| HET-HET | wild type | 37662 | 43063 | 67 | 65 | 2380 | 2258 | 7047 | 8251 | 100793 |
| HET-HET | wild type | 16392 | 18730 | 34 | 40 | 968 | 887 | 3340 | 3839 | 44230 |
| HET-HET | wild type | 24783 | 27451 | 51 | 54 | 1730 | 1572 | 5201 | 5799 | 66641 |
| HET-HET | wild type | 15663 | 17969 | 42 | 35 | 1047 | 914 | 3348 | 3650 | 42668 |
| HET-HET | Total | 162021 | 186586 | 325 | 283 | 10631 | 9883 | 31882 | 37303 | 438914 |
| HET-HET | *fancm* | 13456 | 15845 | 99 | 76 | 1042 | 912 | 3399 | 3949 | 38778 |
| HET-HET | *fancm* | 9676 | 12458 | 84 | 66 | 874 | 737 | 2651 | 3228 | 29774 |
| HET-HET | *fancm* | 7434 | 10211 | 58 | 45 | 761 | 622 | 1973 | 2529 | 23633 |
| HET-HET | *fancm* | 17795 | 24726 | 149 | 86 | 1675 | 1513 | 3993 | 5374 | 55311 |
| HET-HET | *fancm* | 19830 | 26142 | 188 | 104 | 1848 | 1682 | 4803 | 6179 | 60776 |
| HET-HET | *fancm* | 18130 | 23569 | 148 | 97 | 1576 | 1407 | 4196 | 5598 | 54721 |
| HET-HET | Total | 86321 | 112951 | 726 | 474 | 7776 | 6873 | 21015 | 26857 | 262993 |
| HET-HET | *fancm/zip4* | 14298 | 18225 | 43 | 27 | 507 | 575 | 1539 | 1789 | 37003 |
| HET-HET | *fancm/zip4* | 13697 | 17825 | 38 | 23 | 415 | 502 | 1318 | 1530 | 35348 |
| HET-HET | *fancm/zip4* | 22895 | 28211 | 58 | 38 | 696 | 870 | 2124 | 2471 | 57363 |
| HET-HET | *fancm/zip4* | 14662 | 18145 | 41 | 33 | 475 | 539 | 1445 | 1552 | 36892 |
| HET-HET | *fancm/zip4* | 8651 | 11454 | 16 | 11 | 303 | 345 | 826 | 932 | 22538 |
| HET-HET | *fancm/zip4* | 5013 | 7083 | 16 | 7 | 201 | 229 | 483 | 634 | 13666 |
| HET-HET | Total | 79216 | 100943 | 212 | 139 | 2597 | 3060 | 7735 | 8908 | 202810 |
| HET-HOM | wild type | 30141 | 32663 | 88 | 62 | 2516 | 2602 | 6376 | 7017 | 81465 |
| HET-HOM | wild type | 16994 | 18172 | 46 | 26 | 1476 | 1322 | 3961 | 4042 | 46039 |
| HET-HOM | wild type | 47993 | 51441 | 130 | 99 | 4169 | 4057 | 10679 | 11736 | 130304 |
| HET-HOM | wild type | 27808 | 31248 | 160 | 115 | 2478 | 2405 | 6844 | 7668 | 78726 |
| HET-HOM | wild type | 23765 | 25610 | 120 | 96 | 2166 | 1899 | 5000 | 6242 | 64898 |
| HET-HOM | wild type | 30567 | 35395 | 98 | 84 | 2592 | 2486 | 7084 | 7622 | 85928 |
| HET-HOM | wild type | 25524 | 28521 | 74 | 85 | 2337 | 2236 | 5930 | 6145 | 70852 |
| HET-HOM | wild type | 32107 | 36593 | 96 | 87 | 2879 | 2652 | 7298 | 8136 | 89848 |
| HET-HOM | Total | 234899 | 259643 | 812 | 654 | 20613 | 19659 | 53172 | 58608 | 648060 |
| HET-HOM | *fancm* | 28731 | 29789 | 308 | 222 | 2983 | 2889 | 8094 | 8904 | 81920 |
| HET-HOM | *fancm* | 28232 | 30067 | 272 | 209 | 2862 | 2775 | 7746 | 8957 | 81120 |
| HET-HOM | *fancm* | 26843 | 28043 | 281 | 200 | 2821 | 2631 | 7566 | 8276 | 76661 |
| HET-HOM | *fancm* | 19858 | 21710 | 185 | 156 | 2104 | 1876 | 5229 | 6539 | 57657 |
| HET-HOM | *fancm* | 21266 | 23822 | 195 | 132 | 2043 | 2004 | 5585 | 6446 | 61493 |
| HET-HOM | *fancm* | 26014 | 29370 | 270 | 211 | 2693 | 2559 | 7340 | 8286 | 76743 |
| HET-HOM | *fancm* | 21684 | 25214 | 206 | 170 | 2309 | 2035 | 6136 | 6732 | 64486 |
| HET-HOM | *fancm* | 15398 | 19600 | 130 | 100 | 1650 | 1430 | 4118 | 4611 | 47037 |
| HET-HOM | *fancm* | 14369 | 17764 | 161 | 110 | 1557 | 1493 | 4060 | 4517 | 44031 |
| HET-HOM | Total | 202395 | 225379 | 2008 | 1510 | 21022 | 19692 | 55874 | 63268 | 591148 |
| HET-HOM | *fancm/zip4* | 35745 | 37429 | 78 | 59 | 914 | 901 | 2862 | 3255 | 81243 |
| HET-HOM | *fancm/zip4* | 33691 | 35615 | 73 | 61 | 897 | 770 | 2453 | 3074 | 76634 |
| HET-HOM | *fancm/zip4* | 31910 | 34207 | 58 | 44 | 792 | 786 | 2427 | 2776 | 73000 |
| HET-HOM | *fancm/zip4* | 26514 | 28088 | 42 | 50 | 686 | 635 | 2086 | 2338 | 60439 |
| HET-HOM | *fancm/zip4* | 21514 | 23073 | 38 | 29 | 483 | 488 | 1503 | 1735 | 48863 |
| HET-HOM | *fancm/zip4* | 14696 | 16310 | 30 | 16 | 332 | 353 | 963 | 1236 | 33936 |
| HET-HOM | *fancm/zip4* | 26247 | 29615 | 35 | 21 | 644 | 648 | 1847 | 2036 | 61093 |
| HET-HOM | *fancm/zip4* | 30860 | 36272 | 57 | 40 | 787 | 715 | 2014 | 2377 | 73122 |
| HET-HOM | *fancm/zip4* | 31394 | 38761 | 61 | 37 | 788 | 709 | 2149 | 2690 | 76589 |
| HET-HOM | Total | 252571 | 279370 | 472 | 357 | 6323 | 6005 | 18304 | 21517 | 584919 |
| HOM-HET | wild type | 20136 | 22356 | 44 | 42 | 1130 | 1106 | 3180 | 3253 | 51247 |
| HOM-HET | wild type | 19475 | 21615 | 61 | 46 | 1058 | 1136 | 3251 | 3090 | 49732 |
| HOM-HET | wild type | 20332 | 23196 | 54 | 51 | 1175 | 1259 | 3308 | 3297 | 52672 |
| HOM-HET | wild type | 15258 | 18022 | 28 | 20 | 566 | 527 | 1603 | 1992 | 38016 |
| HOM-HET | wild type | 21303 | 24956 | 56 | 49 | 912 | 782 | 2548 | 3061 | 53667 |
| HOM-HET | wild type | 19340 | 22529 | 49 | 33 | 682 | 680 | 2097 | 2449 | 47859 |
| HOM-HET | Total | 115844 | 132674 | 292 | 241 | 5523 | 5490 | 15987 | 17142 | 293193 |
| HOM-HET | *fancm* | 12906 | 14600 | 664 | 555 | 1811 | 1773 | 4246 | 4565 | 41120 |
| HOM-HET | *fancm* | 9518 | 11178 | 450 | 402 | 1303 | 1254 | 3090 | 3379 | 30574 |
| HOM-HET | *fancm* | 7114 | 8210 | 337 | 326 | 1025 | 974 | 2557 | 2625 | 23168 |
| HOM-HET | *fancm* | 16010 | 18007 | 754 | 594 | 2375 | 2568 | 5670 | 5828 | 51806 |
| HOM-HET | *fancm* | 20211 | 22218 | 989 | 819 | 2992 | 3006 | 7364 | 7080 | 64679 |
| HOM-HET | *fancm* | 18661 | 20741 | 847 | 701 | 2819 | 2894 | 6918 | 6581 | 60162 |
| HOM-HET | Total | 84420 | 94954 | 4041 | 3397 | 12325 | 12469 | 29845 | 30058 | 271509 |
| HOM-HET | *fancm/zip4* | 21832 | 23954 | 828 | 621 | 2715 | 3024 | 6427 | 6108 | 65509 |
| HOM-HET | *fancm/zip4* | 15882 | 16706 | 681 | 470 | 2158 | 2274 | 4996 | 4477 | 47644 |
| HOM-HET | *fancm/zip4* | 20680 | 20991 | 831 | 673 | 2789 | 2854 | 6675 | 6028 | 61521 |
| HOM-HET | *fancm zip4* | 21967 | 24131 | 1087 | 1103 | 3311 | 3534 | 7515 | 7708 | 70356 |
| HOM-HET | *fancm zip4* | 12516 | 14132 | 658 | 645 | 1946 | 1932 | 4416 | 4595 | 40840 |
| HOM-HET | *fancm zip4* | 13352 | 15094 | 723 | 681 | 1971 | 2232 | 4727 | 4858 | 43638 |
| HOM-HET | Total | 106229 | 115008 | 4808 | 4193 | 14890 | 15850 | 34756 | 33774 | 329508 |
