# Supplementary material for: Juxtaposition of heterozygous and homozygous regions causes reciprocal crossover remodelling via interference during Arabidopsis meiosis
Source: eLife. 2015 Mar 27;4:e03708. doi: 10.7554/eLife.03708 (PMC4407271; doi:10.7554/eLife.03708)
Supplement: Figure 8—source data 2. — DOI: http://dx.doi.org/10.7554/eLife.03708.037 [file elife03708s016.docx]

**Figure 8 – Source Data 2. Calculation of *I3bc* interference from wild type, *fancm* and *fancm zip4* individuals with varying heterozygosity.** For formulae used to calculate expected and observed DCOs and interference see Materials and Methods section.

| Heterozygosity | | Genotype | *I3b* cM | *I3c* cM | Expected DCOs | Observed DCOs | Interference |
| --- | --- | --- | --- | --- | --- | --- | --- |
| HOM-HOM | wild type | | 16.26 | 4.98 | 149 | 47 | 0.685 |
| HOM-HOM | wild type | | 15.71 | 4.38 | 160 | 68 | 0.575 |
| HOM-HOM | wild type | | 15.42 | 5.00 | 188 | 68 | 0.638 |
| HOM-HOM | Total | | 15.76 | 4.77 | 497 | 183 | 0.632 |
| HOM-HOM | *fancm* | | 27.79 | 14.99 | 1322 | 1228 | 0.071 |
| HOM-HOM | *fancm* | | 28.53 | 13.89 | 1229 | 1122 | 0.087 |
| HOM-HOM | *fancm* | | 27.43 | 13.86 | 1019 | 887 | 0.130 |
| HOM-HOM | *fancm* | | 30.09 | 13.66 | 519 | 456 | 0.121 |
| HOM-HOM | Total | | 28.20 | 14.20 | 4090 | 3693 | 0.097 |
| HOM-HOM | *fancm/zip4* | | 31.42 | 12.67 | 1535 | 1652 | -0.076 |
| HOM-HOM | *fancm/zip4* | | 25.15 | 11.97 | 559 | 592 | -0.060 |
| HOM-HOM | *fancm/zip4* | | 24.17 | 12.48 | 944 | 987 | -0.045 |
| HOM-HOM | Total | | 27.54 | 12.46 | 3032 | 3231 | -0.065 |
| HET-HET | wild type | | 15.47 | 4.83 | 742 | 105 | 0.858 |
| HET-HET | wild type | | 15.91 | 4.90 | 666 | 115 | 0.827 |
| HET-HET | wild type | | 15.31 | 4.73 | 730 | 132 | 0.819 |
| HET-HET | wild type | | 16.40 | 4.36 | 316 | 74 | 0.766 |
| HET-HET | wild type | | 16.66 | 5.11 | 568 | 105 | 0.815 |
| HET-HET | wild type | | 16.58 | 4.78 | 338 | 77 | 0.772 |
| HET-HET | Total | | 15.90 | 4.81 | 3359 | 608 | 0.819 |
| HET-HET | *fancm* | | 19.40 | 5.49 | 413 | 175 | 0.576 |
| HET-HET | *fancm* | | 20.25 | 5.91 | 357 | 150 | 0.579 |
| HET-HET | *fancm* | | 19.49 | 6.29 | 290 | 103 | 0.644 |
| HET-HET | *fancm* | | 17.36 | 6.19 | 594 | 235 | 0.605 |
| HET-HET | *fancm* | | 18.55 | 6.29 | 709 | 292 | 0.588 |
| HET-HET | *fancm* | | 18.35 | 5.90 | 592 | 245 | 0.586 |
| HET-HET | Total | | 18.66 | 6.03 | 2957 | 1200 | 0.594 |
| HET-HET | *fancm/zip4* | | 9.18 | 3.11 | 106 | 70 | 0.338 |
| HET-HET | *fancm/zip4* | | 8.23 | 2.77 | 80 | 61 | 0.242 |
| HET-HET | *fancm/zip4* | | 8.18 | 2.90 | 136 | 96 | 0.294 |
| HET-HET | *fancm/zip4* | | 8.32 | 2.95 | 91 | 74 | 0.183 |
| HET-HET | *fancm/zip4* | | 7.92 | 2.99 | 53 | 27 | 0.495 |
| HET-HET | *fancm/zip4* | | 8.34 | 3.31 | 38 | 23 | 0.391 |
| HET-HET | Total | | 8.38 | 2.96 | 503 | 351 | 0.303 |
| HET-HOM | wild type | | 16.62 | 6.47 | 876 | 150 | 0.829 |
| HET-HOM | wild type | | 17.54 | 6.23 | 503 | 72 | 0.857 |
| HET-HOM | wild type | | 17.38 | 6.49 | 1469 | 229 | 0.844 |
| HET-HOM | wild type | | 18.78 | 6.55 | 969 | 275 | 0.716 |
| HET-HOM | wild type | | 17.66 | 6.60 | 756 | 216 | 0.714 |
| HET-HOM | wild type | | 17.33 | 6.12 | 911 | 182 | 0.800 |
| HET-HOM | wild type | | 17.27 | 6.68 | 817 | 159 | 0.805 |
| HET-HOM | wild type | | 17.38 | 6.36 | 993 | 183 | 0.816 |
| HET-HOM | Total | | 17.47 | 6.44 | 7294 | 1466 | 0.799 |
| HET-HOM | *fancm* | | 21.40 | 7.81 | 1370 | 530 | 0.613 |
| HET-HOM | *fancm* | | 21.18 | 7.54 | 1296 | 481 | 0.629 |
| HET-HOM | *fancm* | | 21.29 | 7.74 | 1263 | 481 | 0.619 |
| HET-HOM | *fancm* | | 21.00 | 7.49 | 907 | 341 | 0.624 |
| HET-HOM | *fancm* | | 20.10 | 7.11 | 879 | 327 | 0.628 |
| HET-HOM | *fancm* | | 20.99 | 7.47 | 1203 | 481 | 0.600 |
| HET-HOM | *fancm* | | 20.54 | 7.32 | 969 | 376 | 0.612 |
| HET-HOM | *fancm* | | 19.05 | 7.04 | 630 | 230 | 0.635 |
| HET-HOM | *fancm* | | 20.09 | 7.54 | 667 | 271 | 0.594 |
| HET-HOM | Total | | 20.75 | 7.48 | 9178 | 3518 | 0.617 |
| HET-HOM | *fancm/zip4* | | 7.70 | 2.40 | 150 | 137 | 0.088 |
| HET-HOM | *fancm/zip4* | | 7.39 | 2.35 | 133 | 134 | -0.007 |
| HET-HOM | *fancm/zip4* | | 7.27 | 2.30 | 122 | 102 | 0.165 |
| HET-HOM | *fancm/zip4* | | 7.47 | 2.34 | 106 | 92 | 0.129 |
| HET-HOM | *fancm/zip4* | | 6.76 | 2.12 | 70 | 67 | 0.046 |
| HET-HOM | *fancm/zip4* | | 6.62 | 2.15 | 48 | 46 | 0.049 |
| HET-HOM | *fancm/zip4* | | 6.45 | 2.21 | 87 | 56 | 0.356 |
| HET-HOM | *fancm/zip4* | | 6.14 | 2.19 | 98 | 97 | 0.012 |
| HET-HOM | *fancm/zip4* | | 6.45 | 2.08 | 103 | 98 | 0.047 |
| HET-HOM | Total | | 6.95 | 2.25 | 914 | 829 | 0.093 |
| HOM-HET | wild type | | 12.72 | 4.53 | 295 | 86 | 0.709 |
| HOM-HET | wild type | | 12.97 | 4.63 | 298 | 107 | 0.641 |
| HOM-HET | wild type | | 12.74 | 4.82 | 323 | 105 | 0.675 |
| HOM-HET | wild type | | 9.58 | 3.00 | 109 | 48 | 0.561 |
| HOM-HET | wild type | | 10.65 | 3.35 | 192 | 105 | 0.452 |
| HOM-HET | wild type | | 9.67 | 3.02 | 140 | 82 | 0.413 |
| HOM-HET | Total | | 11.48 | 3.94 | 1326 | 533 | 0.598 |
| HOM-HET | *fancm* | | 24.39 | 11.68 | 1172 | 1219 | -0.041 |
| HOM-HET | *fancm* | | 23.95 | 11.15 | 816 | 852 | -0.044 |
| HOM-HET | *fancm* | | 25.23 | 11.49 | 672 | 663 | 0.013 |
| HOM-HET | *fancm* | | 24.80 | 12.14 | 1560 | 1348 | 0.136 |
| HOM-HET | *fancm* | | 25.13 | 12.07 | 1961 | 1808 | 0.078 |
| HOM-HET | *fancm* | | 25.01 | 12.07 | 1816 | 1548 | 0.148 |
| HOM-HET | Total | | 24.80 | 11.87 | 7994 | 7438 | 0.070 |
| HOM-HET | *fancm/zip4* | | 21.35 | 10.97 | 1534 | 1449 | 0.056 |
| HOM-HET | *fancm/zip4* | | 22.30 | 11.72 | 1245 | 1151 | 0.075 |
| HOM-HET | *fancm/zip4* | | 23.09 | 11.62 | 1650 | 1504 | 0.089 |
| HOM-HET | *fancm zip4* | | 24.75 | 12.84 | 2236 | 2190 | 0.021 |
| HOM-HET | *fancm zip4* | | 25.25 | 12.69 | 1308 | 1303 | 0.004 |
| HOM-HET | *fancm zip4* | | 25.18 | 12.85 | 1412 | 1404 | 0.006 |
| HOM-HET | Total | | 23.53 | 12.06 | 9351 | 9001 | 0.037 |
