# Supplementary material for: Juxtaposition of heterozygous and homozygous regions causes reciprocal crossover remodelling via interference during Arabidopsis meiosis
Source: eLife. 2015 Mar 27;4:e03708. doi: 10.7554/eLife.03708 (PMC4407271; doi:10.7554/eLife.03708)
Supplement: Figure 8—source data 3. — DOI: http://dx.doi.org/10.7554/eLife.03708.038 [file elife03708s017.docx]

**Figure 8 – Source Data 3. *I3bc* fluorescent seed count data from wild type and *zip4* individuals with varying heterozygosity.** Counts are listed for the 8 pollen fluorescent classes (Fig. 5 - Supplementary Figure 1).

| Heterozygosity | Genotype | BYR | byr | bYr | ByR | BYr | byR | bYR | Byr | Total |
| --- | --- | --- | --- | --- | --- | --- | --- | --- | --- | --- |
| HOM-HOM | Wild type | 27994 | 28764 | 89 | 88 | 1329 | 1327 | 5146 | 5237 | 69204 |
| HOM-HOM | Wild type | 41269 | 42663 | 166 | 169 | 2193 | 2126 | 8294 | 8505 | 103991 |
| HOM-HOM | Wild type | 35985 | 36713 | 140 | 131 | 1782 | 1745 | 6913 | 6888 | 89569 |
| HOM-HOM | Total | 105248 | 108140 | 395 | 388 | 5304 | 5198 | 20353 | 20630 | 262764 |
| HOM-HOM | *zip4* | 2723 | 3388 | 0 | 4 | 55 | 55 | 131 | 124 | 5815 |
| HOM-HOM | *zip4* | 2260 | 3856 | 4 | 1 | 34 | 82 | 86 | 106 | 4833 |
| HOM-HOM | *zip4* | 4150 | 6421 | 4 | 5 | 87 | 123 | 185 | 163 | 8867 |
| HOM-HOM | *zip4* | 2013 | 2707 | 3 | 1 | 33 | 53 | 78 | 79 | 4273 |
| HOM-HOM | *zip4* | 5322 | 7064 | 7 | 7 | 108 | 111 | 232 | 229 | 11338 |
| HOM-HOM | Total | 16468 | 23436 | 18 | 18 | 317 | 424 | 712 | 701 | 35126 |
| HET-HET | Wild type | 8777 | 10905 | 17 | 21 | 598 | 563 | 1908 | 2010 | 22671 |
| HET-HET | Wild type | 9414 | 11067 | 16 | 17 | 591 | 561 | 1904 | 2124 | 24041 |
| HET-HET | Wild type | 10307 | 14073 | 35 | 31 | 770 | 788 | 2381 | 2473 | 27092 |
| HET-HET | Wild type | 18031 | 22638 | 51 | 53 | 1341 | 1308 | 4189 | 4265 | 47269 |
| HET-HET | Wild type | 26010 | 31107 | 43 | 32 | 1342 | 1247 | 4336 | 4426 | 63446 |
| HET-HET | Wild type | 22913 | 27513 | 37 | 37 | 1228 | 1162 | 3734 | 4069 | 56093 |
| HET-HET | Wild type | 11598 | 16614 | 14 | 21 | 602 | 600 | 1999 | 2055 | 28487 |
| HET-HET | Total | 107050 | 133917 | 213 | 212 | 6472 | 6229 | 20451 | 21422 | 269099 |
| HET-HET | *zip4* | 2481 | 3488 | 1 | 1 | 33 | 49 | 71 | 76 | 5193 |
| HET-HET | *zip4* | 2431 | 4425 | 0 | 3 | 34 | 53 | 90 | 108 | 5150 |
| HET-HET | *zip4* | 2020 | 3665 | 0 | 5 | 30 | 70 | 63 | 69 | 4277 |
| HET-HET | *zip4* | 4721 | 9402 | 1 | 4 | 60 | 75 | 173 | 173 | 9928 |
| HET-HET | *zip4* | 5133 | 7358 | 2 | 3 | 66 | 81 | 139 | 163 | 10720 |
| HET-HET | *zip4* | 2815 | 4135 | 1 | 3 | 43 | 46 | 90 | 101 | 5914 |
| HET-HET | Total | 19601 | 32473 | 5 | 19 | 266 | 374 | 626 | 690 | 41182 |
| HOM-HET | Wild type | 6427 | 7406 | 13 | 15 | 204 | 197 | 610 | 598 | 14491 |
| HOM-HET | Wild type | 12892 | 16738 | 23 | 23 | 451 | 429 | 1342 | 1408 | 29460 |
| HOM-HET | Wild type | 19073 | 23000 | 43 | 32 | 645 | 648 | 2008 | 2062 | 43584 |
| HOM-HET | Total | 535126 | 643076 | 1341 | 1344 | 26018 | 25724 | 88244 | 90954 | 1303877 |
| HOM-HET | zip4 | 1130 | 1416 | 0 | 2 | 11 | 39 | 23 | 46 | 2381 |
| HOM-HET | zip4 | 974 | 1483 | 0 | 2 | 18 | 30 | 35 | 56 | 2089 |
| HOM-HET | zip4 | 3215 | 3215 | 2 | 1 | 70 | 106 | 174 | 173 | 6956 |
| HOM-HET | zip4 | 3342 | 5531 | 2 | 3 | 69 | 73 | 152 | 145 | 7128 |
| HOM-HET | Total | 8661 | 11645 | 4 | 8 | 168 | 248 | 384 | 420 | 18554 |
| HET-HOM | Wild type | 10101 | 12587 | 32 | 25 | 801 | 772 | 2044 | 2393 | 26269 |
| HET-HOM | Wild type | 9700 | 12373 | 28 | 19 | 809 | 771 | 2029 | 2173 | 25229 |
| HET-HOM | Wild type | 10275 | 12551 | 29 | 21 | 778 | 809 | 2126 | 2334 | 26647 |
| HET-HOM | Wild type | 9536 | 11044 | 19 | 15 | 708 | 724 | 2072 | 2089 | 24699 |
| HET-HOM | Wild type | 8700 | 11421 | 24 | 20 | 648 | 737 | 1854 | 1932 | 22615 |
| HET-HOM | Wild type | 42310 | 47228 | 120 | 137 | 3514 | 3417 | 9397 | 10183 | 111388 |
| HET-HOM | Wild type | 20552 | 21684 | 70 | 72 | 1984 | 1820 | 4675 | 5210 | 54935 |
| HET-HOM | Wild type | 27303 | 26291 | 84 | 123 | 2493 | 2404 | 6279 | 6754 | 72743 |
| HET-HOM | Wild type | 17040 | 16685 | 50 | 50 | 1592 | 1522 | 3952 | 4155 | 45401 |
| HET-HOM | Total | 155517 | 171864 | 456 | 482 | 13327 | 12976 | 34428 | 37223 | 409926 |
| HET-HOM | *zip4* | 3094 | 4330 | 1 | 1 | 38 | 53 | 86 | 85 | 6452 |
| HET-HOM | *zip4* | 5084 | 7153 | 4 | 4 | 48 | 76 | 133 | 154 | 10587 |
| HET-HOM | *zip4* | 3085 | 4554 | 1 | 0 | 45 | 68 | 98 | 136 | 6518 |
| HET-HOM | *zip4* | 3701 | 4860 | 5 | 2 | 57 | 49 | 108 | 117 | 7740 |
| HET-HOM | *zip4* | 3464 | 4148 | 3 | 2 | 44 | 46 | 97 | 121 | 7241 |
| HET-HOM | *zip4* | 6312 | 7176 | 5 | 5 | 77 | 76 | 168 | 166 | 13121 |
| HET-HOM | *zip4* | 5590 | 8802 | 1 | 5 | 72 | 69 | 121 | 122 | 11570 |
| HET-HOM | Total | 30330 | 41023 | 20 | 19 | 381 | 437 | 811 | 901 | 63229 |
