# Supplementary material for: Juxtaposition of heterozygous and homozygous regions causes reciprocal crossover remodelling via interference during Arabidopsis meiosis
Source: eLife. 2015 Mar 27;4:e03708. doi: 10.7554/eLife.03708 (PMC4407271; doi:10.7554/eLife.03708)
Supplement: Figure 8—source data 4. — DOI: http://dx.doi.org/10.7554/eLife.03708.039 [file elife03708s018.docx]

**Figure 8 – Source Data 4. Calculation of *I3bc* interference in wild type and *zip4*.** For formulae used to calculate expected and observed DCOs and interference see Materials and Methods section.

| Heterozygosity | Genotype | *I3b* cM | *I3c* cM | Expected DCOs | Observed DCOs | Interference |
| --- | --- | --- | --- | --- | --- | --- |
| HOM-HOM | Wild type | 15.26 | 4.09 | 432 | 177 | 0.59 |
| HOM-HOM | Wild type | 16.48 | 4.48 | 767 | 335 | 0.56 |
| HOM-HOM | Wild type | 15.71 | 4.24 | 597 | 271 | 0.55 |
| HOM-HOM | Wild type | 15.89 | 4.29 | 1794 | 783 | 0.56 |
| HOM-HOM | zip4 | 4.45 | 1.96 | 5 | 4 | 0.21 |
| HOM-HOM | zip4 | 4.08 | 2.50 | 5 | 5 | -0.01 |
| HOM-HOM | zip4 | 4.03 | 2.47 | 9 | 9 | -0.02 |
| HOM-HOM | zip4 | 3.77 | 2.11 | 3 | 4 | -0.18 |
| HOM-HOM | zip4 | 4.19 | 2.06 | 10 | 14 | -0.43 |
| HOM-HOM | zip4 | 4.13 | 2.21 | 32 | 36 | -0.12 |
| HET-HET | Wild type | 17.45 | 5.29 | 209 | 38 | 0.82 |
| HET-HET | Wild type | 16.89 | 4.93 | 200 | 33 | 0.84 |
| HET-HET | Wild type | 18.16 | 5.99 | 295 | 66 | 0.78 |
| HET-HET | Wild type | 18.10 | 5.82 | 498 | 104 | 0.79 |
| HET-HET | Wild type | 13.93 | 4.20 | 371 | 75 | 0.80 |
| HET-HET | Wild type | 14.04 | 4.39 | 346 | 74 | 0.79 |
| HET-HET | Wild type | 14.35 | 4.34 | 178 | 35 | 0.80 |
| HET-HET | Wild type | 15.72 | 4.88 | 2063 | 425 | 0.79 |
| HET-HET | zip4 | 2.87 | 1.62 | 2 | 2 | 0.17 |
| HET-HET | zip4 | 3.90 | 1.75 | 4 | 3 | 0.15 |
| HET-HET | zip4 | 3.20 | 2.45 | 3 | 5 | -0.49 |
| HET-HET | zip4 | 3.54 | 1.41 | 5 | 5 | -0.01 |
| HET-HET | zip4 | 2.86 | 1.42 | 4 | 5 | -0.15 |
| HET-HET | zip4 | 3.30 | 1.57 | 3 | 4 | -0.30 |
| HET-HET | zip4 | 3.25 | 1.61 | 22 | 24 | -0.11 |
| HET-HOM | Wild type | 17.11 | 6.21 | 279 | 57 | 0.80 |
| HET-HOM | Wild type | 16.84 | 6.45 | 274 | 47 | 0.83 |
| HET-HOM | Wild type | 16.92 | 6.14 | 277 | 50 | 0.82 |
| HET-HOM | Wild type | 16.98 | 5.94 | 249 | 34 | 0.86 |
| HET-HOM | Wild type | 16.94 | 6.32 | 242 | 44 | 0.82 |
| HET-HOM | Wild type | 17.81 | 6.45 | 1280 | 257 | 0.80 |
| HET-HOM | Wild type | 18.25 | 7.18 | 720 | 142 | 0.80 |
| HET-HOM | Wild type | 18.20 | 7.02 | 929 | 207 | 0.78 |
| HET-HOM | Wild type | 18.08 | 7.08 | 581 | 100 | 0.83 |
| HET-HOM | Wild type | 17.71 | 6.65 | 4824 | 938 | 0.81 |
| HET-HOM | zip4 | 2.68 | 1.44 | 2 | 2 | 0.20 |
| HET-HOM | zip4 | 2.79 | 1.25 | 4 | 8 | -1.18 |
| HET-HOM | zip4 | 3.61 | 1.75 | 4 | 1 | 0.76 |
| HET-HOM | zip4 | 3.00 | 1.46 | 3 | 7 | -1.07 |
| HET-HOM | zip4 | 3.08 | 1.31 | 3 | 5 | -0.71 |
| HET-HOM | zip4 | 2.62 | 1.24 | 4 | 10 | -1.34 |
| HET-HOM | zip4 | 2.15 | 1.27 | 3 | 6 | -0.90 |
| HET-HOM | zip4 | 2.77 | 1.36 | 24 | 39 | -0.64 |
| HOM-HET | Wild type | 8.53 | 2.96 | 37 | 28 | 0.23 |
| HOM-HET | Wild type | 9.49 | 3.14 | 88 | 46 | 0.48 |
| HOM-HET | Wild type | 9.51 | 3.14 | 130 | 75 | 0.42 |
| HOM-HET | Wild type | 7.39 | 2.63 | 250 | 173 | 0.31 |
| HOM-HET | zip4 | 2.98 | 2.18 | 2 | 2 | -0.29 |
| HOM-HET | zip4 | 4.45 | 2.39 | 2 | 2 | 0.10 |
| HOM-HET | zip4 | 5.03 | 2.57 | 9 | 3 | 0.67 |
| HOM-HET | zip4 | 4.24 | 2.06 | 6 | 5 | 0.20 |
| HOM-HET | zip4 | 4.40 | 2.31 | 19 | 12 | 0.36 |
