# Supplementary material for: Juxtaposition of heterozygous and homozygous regions causes reciprocal crossover remodelling via interference during Arabidopsis meiosis
Source: eLife. 2015 Mar 27;4:e03708. doi: 10.7554/eLife.03708 (PMC4407271; doi:10.7554/eLife.03708)
Supplement: Supplementary file 1. — Oligonucleotides used to genotype Col-0/Ct-1 polymorphisms. DOI: http://dx.doi.org/10.7554/eLife.03708.043 [file elife03708s019.docx]

**Supplementary File 1. Oligonucleotides used to genotype Col-0/Ct-1 polymorphisms.**

| Primer name | Chr | Col-0 size (bp) | Ct-1 size  (bp) | Indel size (bp) | Sequence (5’-3’) | T_m_ | TAIR10 coordinate | Adjacent gene |
| --- | --- | --- | --- | --- | --- | --- | --- | --- |
| 1-309-F | 1 | 271 | 224 | 47 | CCAACAATTAGCCCGGATGA | 63.0 | 309362 | At1g01900 |
| 1-309-R |  |  |  |  | TATTTTACCCGCAGGCCTCA | 62.7 |  |  |
| 1-1771-F | 1 | 453 | 353 | 100 | CAATCAAGCGAGGAGCAACA | 62.4 | 1771076 | At1g05860 |
| 1-1771-R |  |  |  |  | TTCCGAGTACGCATTGCTCA | 62.8 |  |  |
| 1-3830-F | 1 | 130 | 98 | 32 | CAACAATGGTGATATTTGTTTTGC | 60.5 | 3830600 | At1g11370 |
| 1-3830-R |  |  |  |  | TCAACAATGGGAAATGATCAAA | 60.2 |  |  |
| 1-7294-F | 1 | 199 | 162 | 37 | TTCAAAACTGGAGCGTCGTC | 61.4 | 7294957 | At1g20930 |
| 1-7294-R |  |  |  |  | GGCCCATCTTGTGTGTTTTG | 61.3 |  |  |
| 1-10655-F | 1 | 230 | 167 | 63 | TTGTGGTCCCTGGCTAATCA | 61.4 | 10655852 | At1g30270 |
| 1-10655-R |  |  |  |  | CAGTGACGAATTCCAAAACGA | 61.0 |  |  |
| 1-14122-F | 1 | 239 | 189 | 50 | GCTAGCAGTCGAGTATTCTGTCGAG | 63.0 | 14122817 | At1g37100 |
| 1-14122-R |  |  |  |  | CGTGTCCCACCATCATCAC | 60.8 |  |  |
| 1-16908-F | 1 | 110 | 82 | 28 | GCACAGAAAGACAAACCCAAAG | 61.0 | 16907783 | At1g44770 |
| 1-16908-R |  |  |  |  | CGACCAGCAAGGTTGTTCTTAG | 61.2 |  |  |
| 1-20154-F | 1 | 620 | 358 | 262 | TCCCAACTGGTAATGATATTTATTTTC | 59.8 | 20154053 | At1g54000 |
| 1-20154-R |  |  |  |  | CCGAATCAAAATCGGAATCTT | 60.3 |  |  |
| 1-23477-F | 1 | 183 | 129 | 54 | TGCTTTTCCTTTTTAATCTTTTTCTCA | 61.8 | 23477122 | At1g63295 |
| 1-23477-R |  |  |  |  | TGATGATTTGTTTTAATCCGCTCA | 62.7 |  |  |
| 1-27077-F | 1 | 365 | 278 | 87 | ATCGGAATGCGGAAGACACT | 62.0 | 27077150 | At1g71930 |
| 1-27077-R |  |  |  |  | CCACCCAGCCTTCCTCCTAT | 62.6 |  |  |
| 1-30413-F | 1 | 135 | 104 | 31 | CCAGCCACAGCTTCTTTCTGA | 62.9 | 30412519 | At1g80950 |
| 1-30413-R |  |  |  |  | TTGATTGAATAATGGTTCTTGTGATGA | 62.7 |  |  |
| 2-132-F | 2 | 229 | 162 | 67 | TCCAATGGGCCACAAATTAAC | 61.8 | 132648 | At2g01250 |
| 2-132-R |  |  |  |  | TTTGTGCTTTGATTACTGCAAGTG | 61.5 |  |  |
| 2-2346-F | 2 | 347 | 261 | 86 | GGCAAATTTGGTTGGCTCTC | 61.9 | 2346993 | At2g06020 |
| 2-2346-R |  |  |  |  | TGTTTTGTGCTATTTGTGTCAACC | 61.2 |  |  |
| 2-4748-F | 2 | 291 | 237 | 54 | TCGTCAAAACCGGAAAACCT | 61.8 | 4748219 | At2g11810 |
| 2-4748-R |  |  |  |  | TCCAATCTTACACATTCCATTTGC | 62.2 |  |  |
| 2-6789-F | 2 | 112 | 82 | 30 | GCGTTTTGTATCATCAAAGGTTCC | 62.9 | 6789815 | At2g15560 |
| 2-6789-R |  |  |  |  | CGCAATTTCTCGAACTTCCTTT | 62.9 |  |  |
| 2-9168-F | 2 | 218 | 152 | 66 | ACCATGCCGCAAATGACATA | 62.2 | 9168713 | At2g21410 |
| 2-9168-R |  |  |  |  | TGTTGAACATTTATTGAAGCATCTTA | 59.2 |  |  |
| 2-11443-F | 2 | 200 | 141 | 59 | GGTTCCGTCAACTTCGAAAA | 60.1 | 11443153 | At2g26830 |
| 2-11443-R |  |  |  |  | CAGTCATTAGAAATCGATCCCACA | 62.4 |  |  |
| 2-13642-F | 2 | 200 | 153 | 47 | AAAGAGCCAACTTGTATCGTCTTC | 60.2 | 13642164 | At2g32200 |
| 2-13642-R |  |  |  |  | CCCTGGATGATGCAAAGAAT | 59.9 |  |  |
| 2-15964-F | 2 | 171 | 120 | 51 | TGCAGCACTGTGTTTTAATTTTAGTC | 60.9 | 15964454 | At2g38120 |
| 2-15964-R |  |  |  |  | TTTGAGTTTGTTGACCCTGAGAA | 61.0 |  |  |
| 2-19311-F | 2 | 140 | 101 | 39 | TTTCTGCCAATGATTTAAAGTAACG | 60.7 | 19311521 | At2g47000 |
| 2-19311-R |  |  |  |  | CAGCGCTGATGCAAAGGTAA | 62.4 |  |  |
| 3-5-F | 3 | 344 | 279 | 65 | ccacaagtcacggaagaaca | 59.7 | 4509 | At3g01010 |
| 3-5-R |  |  |  |  | taagcaagagcaggcatgtg | 60.2 |  |  |
| 3-259-F | 3 | 255 | 204 | 51 | TGAGGCAATCCGGTTTTGAT | 62.7 | 259868 | At3g01710 |
| 3-259-R |  |  |  |  | GAGACCAAAATCAACAGACCAAAC | 61.1 |  |  |
| 3-458-F | 3 | 128 | 104 | 24 | ccccataaaagcagtgagattt | 59.5 | 458229 | At3g02280 |
| 3-458-R |  |  |  |  | gatgactttgtattttgaactttttga | 59.1 |  |  |
| 3-845-F | 3 | 314 | 271 | 43 | tgttgaaattggtctgtgtcaag | 60.1 | 845398 | At3g03530 |
| 3-845-R |  |  |  |  | ccgtaacgtgtgttcttgtttt | 59.1 |  |  |
| 3-1031-F | 3 | 419 | 345 | 74 | atgccttggtttcaatttgg | 59.8 | 1031481 | At3g03980 |
| 3-1031-R |  |  |  |  | tacccgctccttgacagttt | 59.7 |  |  |
| 3-1291-F | 3 | 237 | 183 | 54 | ccatgcatgattacgatgga | 60.3 | 1291982 | At3g04732 |
| 3-1291-R |  |  |  |  | ccacacgagggtcctaacat | 59.8 |  |  |
| 3-1500-F | 3 | 371 | 295 | 76 | tagccgccgtctacataacc | 60.1 | 1500250 | At3g05270 |
| 3-1500-R |  |  |  |  | cgtcgtctctctcagaaatcg | 60.1 |  |  |
| 3-1800-F | 3 | 146 | 104 | 42 | tggtagataatgtgaaaccaatcaa | 59.7 | 1800332 | At3g05990 |
| 3-1800-R |  |  |  |  | ttgatctcgatccataggtgtg | 60.0 |  |  |
| 3-2206-F | 3 | 564 | 421 | 143 | tgcagttttcgagcacattc | 60.0 | 2206781 | At3g06990 |
| 3-2206-R |  |  |  |  | ccatcccacacatctttccttac | 61.8 |  |  |
| 3-2718-F | 3 | 223 | 185 | 38 | ACAACTGGGCGACTCACCTT | 62.1 | 2718687 | At3g08940 |
| 3-2718-R |  |  |  |  | CGTAAACACAAACTGCGAGGT | 60.2 |  |  |
| 3-3047-F | 3 | 300 | 258 | 42 | tggttaccacggacagatttt | 42.9 | 3047036 | At3g09920 |
| 3-3047-R |  |  |  |  | cggcaactagcaagagacg | 57.9 |  |  |
| 3-3520-F | 3 | 229 | 173 | 56 | ctcggcttcgcatctagttc | 60.1 | 3520343 | At3g11240 |
| 3-3520-R |  |  |  |  | caatccgcttgaaagcaagt | 60.4 |  |  |
| 3-4049-F | 3 | 275 | 236 | 39 | gcaaataggaatcagaagttgga | 59.6 | 4049064 | At3g12740 |
| 3-4049-R |  |  |  |  | tttaaaaaggcctccgcttt | 60.2 |  |  |
| 3-4497-F | 3 | 591 | 405 | 186 | aaagagaaaaccatcacccagt | 59.0 | 4497605 | At3g13730 |
| 3-4497-R |  |  |  |  | cgatagcacacatgccaact | 59.7 |  |  |
| 3-4998-F | 3 | 307 | 256 | 51 | cctccgtgtagtgtaagtgaaca | 59.2 | 4997938 | At3g14855 |
| 3-4998-R |  |  |  |  | tctatgtgtttggcatggaga | 59.1 |  |  |
| 3-5352-F | 3 | 533 | 311 | 222 | GGTTCCAATCCACATATCTCTCC | 60.9 | 5352423 | At3g15820 |
| 3-5352-R |  |  |  |  | CACAATCTATCACTCCCGACCA | 62.2 |  |  |
| 3-5704-F | 3 | 276 | 216 | 60 | tctttggatgcatcatggac | 59.5 | 5704514 | At3g16760 |
| 3-5704-R |  |  |  |  | gacgcgatcccaagaactgt | 62.5 |  |  |
| 3-6375-F | 3 | 189 | 141 | 48 | catgggggaacgaaactgaa | 63.1 | 6375806 | At3g18535 |
| 3-6375-R |  |  |  |  | aaaccaactgcttggctgct | 62.3 |  |  |
| 3-6978-F | 3 | 454 | 242 | 212 | tgcccataaggcttcttcca | 62.9 | 6978064 | At3g20015 |
| 3-6978-R |  |  |  |  | gcgtgaagacattggggagt | 62.4 |  |  |
| 3-7674-F | 3 | 498 | 290 | 208 | cggtgttctgtcgctggaag | 64.2 | 7674863 | At3g21780 |
| 3-7674-R |  |  |  |  | ggacggtggatcgtcagaaa | 63.4 |  |  |
| 3-8495-F | 3 | 177 | 132 | 45 | AACGAAAAAGGGGGAATATGAA | 60.8 | 8495131 | At3g23633 |
| 3-8495-R |  |  |  |  | GGGCTTTAAAAAGCAAAAGCA | 60.7 |  |  |
| 3-9404-F | 3 | 384 | 297 | 87 | aacggtccaggttcctcctc | 62.7 | 9404279 | At3g25760 |
| 3-9404-R |  |  |  |  | ttggttttaaggctctggaatca | 62.1 |  |  |
| 3-10695-F | 3 | 161 | 122 | 39 | gagggatgcaaggaggatca | 62.5 | 10695968 | At3g28540 |
| 3-10695-R |  |  |  |  | ttcatcacatcaacgctccaa | 62.6 |  |  |
| 3-11649-F | 3 | 228 | 188 | 40 | TTTAGCCAAACATGCCCAAAT | 61.5 | 11649496 | At3g29770 |
| 3-11649-R |  |  |  |  | CCAAGCGCCAAAACTACCTC | 62.0 |  |  |
| 3-12356-F | 3 | 455 | 315 | 140 | ctacgcccggtgtatttgga | 63.0 | 12356948 | At3g30730 |
| 3-12356-R |  |  |  |  | gcttgtgaggctatgtggctta | 61.6 |  |  |
| 3-2101-F | 3 | 574 | 438 | 136 | tgaatcgattgtacaaatgttaggtt | 60.1 | 2101842 | At3g36659 |
| 3-2101-R |  |  |  |  | gagatgtgagatagaacgaaaacg | 59.3 |  |  |
| 3-15949-F | 3 | 465 | 382 | 83 | CCACCCTCCAGGGAAGAAGT | 62.7 | 15949551 | At3g44250 |
| 3-15949-R |  |  |  |  | GGCAGCGACTGGCTTGTTTA | 63.6 |  |  |
| 3-17088-F | 3 | 560 | 360 | 200 | gctcttgaggttttagggttgtt | 60.0 | 17088210 | At3g46430 |
| 3-17088-R |  |  |  |  | tgcgttcgcatgattcaaaa | 63.6 |  |  |
| 3-19165-F | 3 | 284 | 234 | 50 | TACGTCGCCCTCGAAGAAAT | 62.0 | 19165521 | At3g51660 |
| 3-19165-R |  |  |  |  | GCGCTACATACGCACCACAT | 62.0 |  |  |
| 3-21008-F | 3 | 211 | 172 | 39 | ccgacgttgtgtttctatttcc | 60.8 | 21008135 | At3g56710 |
| 3-21008-R |  |  |  |  | tgagggaacaaggacctaacca | 63.0 |  |  |
| 3-22076-F | 3 | 231 | 171 | 60 | tcggaacttacttgacatattctacc | 59.1 | 22076576 | At3g59765 |
| 3-22076-R |  |  |  |  | tcggggttgttcttagtcgag | 61.5 |  |  |
| 3-23040-F | 3 | 228 | 180 | 48 | TGCTACGACACGCAAACACA | 62.5 | 23040094 | At3g62260 |
| 3-23040-R |  |  |  |  | CGACTTCTCCTGTGGTAAGTCTTG | 61.5 |  |  |
| 4-230-F | 4 | 267 | 209 | 58 | GCGTTCACCTTTAGCATTCCA | 62.4 | 230388 | At4g00520 |
| 4-230-R |  |  |  |  | GCAGCTACACTCATGCCCTCT | 61.9 |  |  |
| 4-2450-F | 4 | 242 | 184 | 58 | GCGATGATGTGCTTAGGTTGG | 62.8 | 2450565 | At4g04840 |
| 4-2450-R |  |  |  |  | GGATTCAATCACATTTCTTTTCAA | 59.4 |  |  |
| 4-4852-F | 4 | 146 | 108 | 38 | TGGGCCAACGACTCTGTTTA | 61.6 | 4852373 | At4g08028 |
| 4-4852-R |  |  |  |  | TCGTTGTCGAACAACACACC | 60.6 |  |  |
| 4-6927-F | 4 | 168 | 129 | 39 | TGAAAGGAGCATACCGTTGAGA | 62.0 | 6927139 | At4g11385 |
| 4-6927-R |  |  |  |  | GCATCATGACATGCTTGTGG | 61.1 |  |  |
| 4-9652-F | 4 | 234 | 172 | 62 | GTTGCCCACTTGTGTGGTCT | 61.0 | 9652287 | At4g17200 |
| 4-9652-R |  |  |  |  | TCTTGTTTGGATGTGAAATTGGA | 61.6 |  |  |
| 4-12981-F | 4 | 180 | 128 | 52 | GCTGAGGTACAATATCTCGAGCTTAC | 61.0 | 12981959 | At4g25400 |
| 4-12981-R |  |  |  |  | GACAAGATCGAAAACATTAACAAAGT | 59.1 |  |  |
| 4-15692-F | 4 | 145 | 113 | 32 | GAACGCAATAAACAGAGAGACCA | 60.7 | 15692444 | At4g32520 |
| 4-15692-R |  |  |  |  | TTTGCGATAAAACAAGGATTTTT | 59.1 |  |  |
| 4-18526-F | 4 | 478 | 319 | 159 | GACGAACAAGGCAACCCATT | 62.2 | 18526361 | At4g39950 |
| 4-18526-R |  |  |  |  | CCGGTTTGTTCACCATCTCC | 62.6 |  |  |
| 5-53-F | 5 | 133 | 93 | 40 | TCTGCATGGGAAATCTCTGG | 61.1 | 53020 | At5g01150 |
| 5-53-R |  |  |  |  | GGAAATTATAGAAAGACGGAAGTGC | 60.7 |  |  |
| 5-3750-F | 5 | 137 | 97 | 40 | ATGGTGGACCTGGGGGTAAC | 63.1 | 3750331 | At5g11660 |
| 5-3750-R |  |  |  |  | GCATGTAGGAAACACAAATCCTGA | 62.4 |  |  |
| 5-7064-F | 5 | 267 | 220 | 47 | ACTGGCCTCGCCTTTCACTA | 62.2 | 7064379 | At5g20840 |
| 5-7064-R |  |  |  |  | AATCACAACTGTGCCCTCGTT | 61.9 |  |  |
| 5-10406-F | 5 | 350 | 272 | 78 | TGTATAATTAGAGCCGTTCGTCGT | 61.2 | 10406321 | At5g28468 |
| 5-10406-R |  |  |  |  | TTTTGAAACTATCCAAATTACCCAAA | 61.0 |  |  |
| 5-13155-F | 5 | 164 | 126 | 38 | GCGGACAATGAACTGATGGA | 62.0 | 13155137 | At5g34863 |
| 5-13155-R |  |  |  |  | TTCGCCTTAGAAATTCTGCCTA | 60.3 |  |  |
| 5-16428-F | 5 | 229 | 183 | 46 | TGTTGCCATGTTGATTTGATTG | 61.7 | 16428466 | At5g41030 |
| 5-16428-R |  |  |  |  | AGATTCAAGGTGGGGCGTGT | 64.1 |  |  |
| 5-19994-F | 5 | 169 | 109 | 60 | TCTAAACCGAACTAAACCGTGAA | 60.0 | 19994907 | At5g49320 |
| 5-19994-R |  |  |  |  | CAAACCAAAACCTACTTTTTCCAA | 60.9 |  |  |
| 5-23287-F | 5 | 204 | 151 | 53 | GAGATGTTGAGAAGCAGAGGAAA | 60.0 | 23287613 | At5g57500 |
| 5-23287-R |  |  |  |  | TGGCGTGAAATACTGAAGCAA | 61.7 |  |  |
| 5-26907-F | 5 | 270 | 200 | 70 | TGTGGATCTTTATGACGTGTGC | 61.0 | 26907352 | At5g67420 |
| 5-26907-R |  |  |  |  | ACCATCTACTTCCATTCAAATAACG | 59.7 |  |  |
